# Supplementary material for: Flexible inhibitory control of visually evoked defensive behavior by the ventral lateral geniculate nucleus
Source: Neuron. 2021 Dec 1;109(23):3810–3822.e9. doi: 10.1016/j.neuron.2021.09.003 (PMC8648186; doi:10.1016/j.neuron.2021.09.003)
Supplement: Document S1. Figures S1–S10 [file mmc1.pdf]

Neuron, Volume *109*

Supplemental information

Flexible inhibitory control of visually evoked defensive behavior by the ventral lateral geniculate nucleus

Alex Fratzl, Alice M. Koltchev, Nicole Vissers, Yu Lin Tan, Andre Marques-Smith, A. Vanessa Stempel, Tiago Branco, and Sonja B. Hofer

Supplemental Figure S1

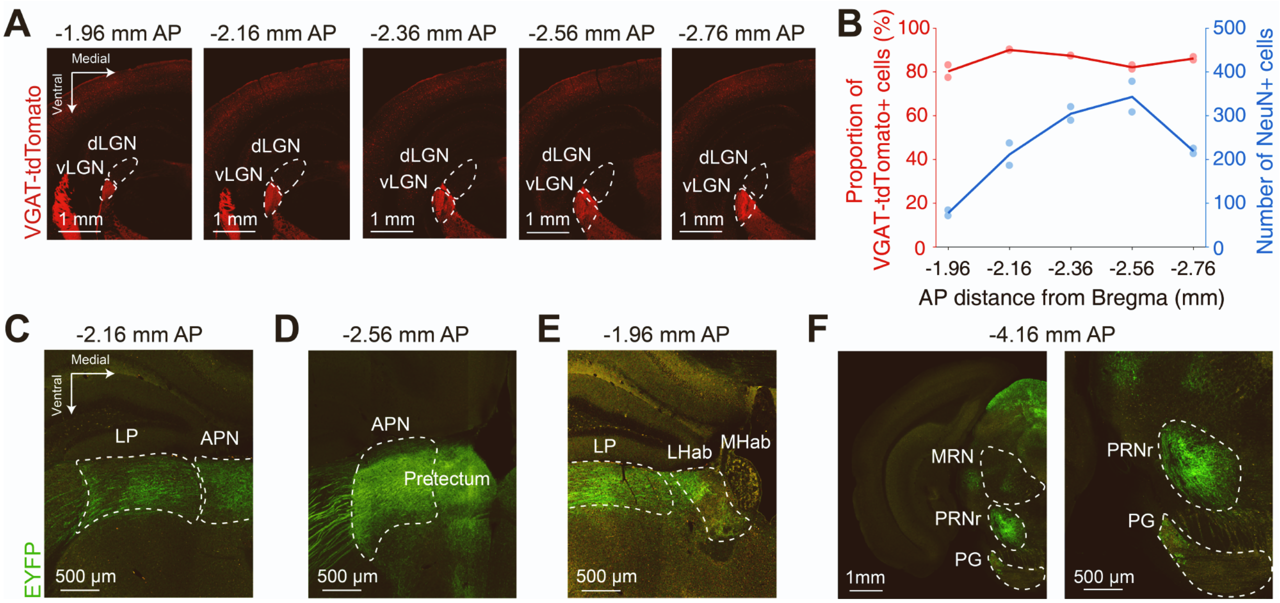

Supplemental Figure S1. vLGN VGAT expression and projection targets. Related to Figure 1.

(A) Images of tdTomato expression in VGAT-positive neurons at different positions along the anterior-posterior (AP) axis in coronal slices. **(B)** VGAT-tdTomato+ cells (red) as a proportion of all NeuN+ cells in the vLGN, and the total amount of NeuN+ cells (blue) in the vLGN at various positions along the AP axis (n = 2 mice, 20 sections). **(C-F)** EYFP-labelled GABAergic vLGN axons in different target areas in coronal slices. APN: anterior pretectal nucleus, LP: lateral posterior nucleus of the thalamus, LHab: lateral habenula, MHab: medial habenula, MRN: midbrain reticular nucleus, PG: pontine grey, PRNr: pontine reticular nucleus. In all panels, distance is in mm from Bregma.

Supplemental Figure S2

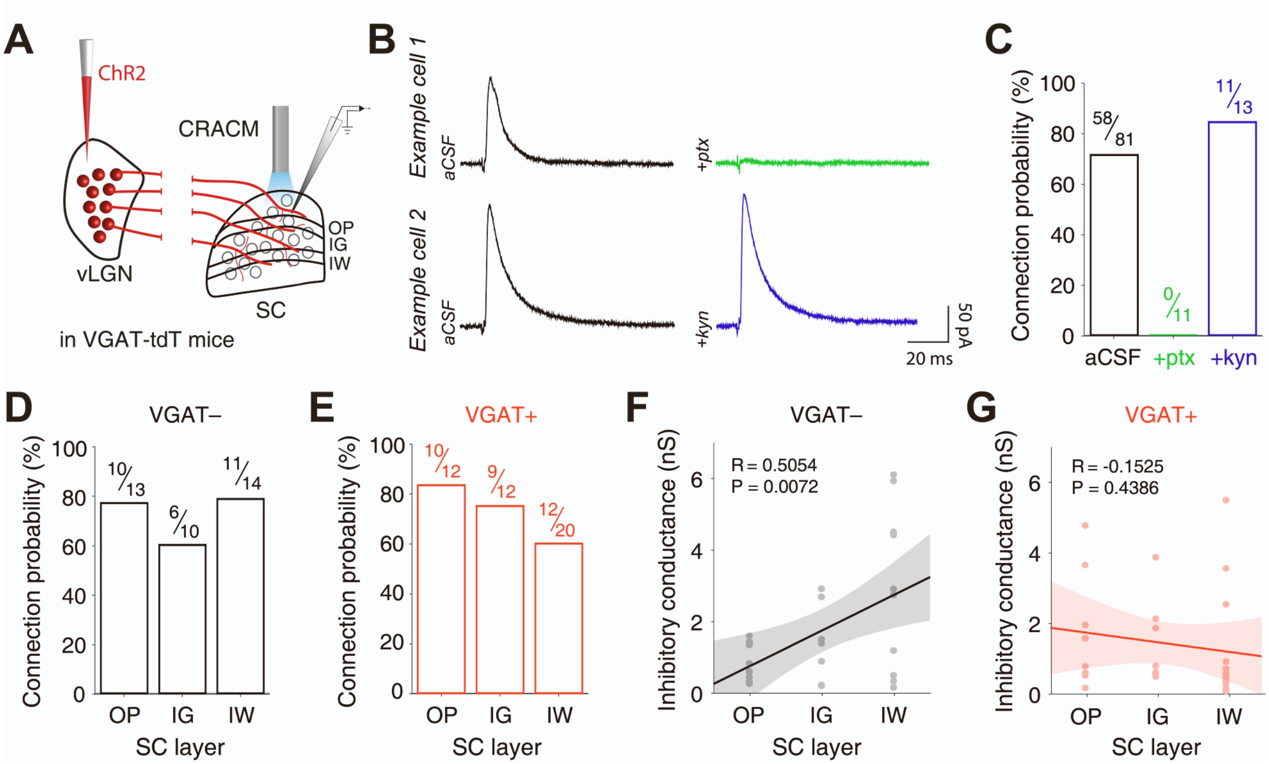

Supplemental Figure S2. Synaptic connectivity of vLGN axons in SC. Related to Figure 1.

(A) Schematic of the experimental approach: VGAT-tdTomato mice were injected in vLGN with ChR2. After two weeks, ChR2-assisted circuit mapping (CRACM) was performed in acute slices, with whole-cell recordings in different mSC layers (OP: optic layer, IG: intermediate grey, and IW: intermediate white). **(B)** Example single-cell recordings performed under control conditions (black), and after bath-application of picrotoxin (ptx, green), or after bath-application of kynurenic acid (kyn, blue) in acute brain slices. **(C)** Connection probability between vLGN axons and cells in the mSC, established in control conditions (n = 81 cells), in bath-applied picrotoxin (n = 11 cells) or in bath-applied kynurenic acid (n = 13 cells) in acute brain slices. **(D,E)** Connection probability between vLGN axons and VGAT-negative **(D)** and VGAT-positive **(E)** neurons in the mSC, split by anatomical SC layers. **(F,G)** Inhibitory synaptic conductance (nS) in VGAT-negative **(F)** and VGAT-positive **(G)** neurons within OP, IG, and IW layers of the mSC. R: Pearson correlation coefficient, P: p-value for slope different from 0 derived from a linear regression model. Shading shows 95% confidence interval of the linear fit.

Supplemental Figure S3

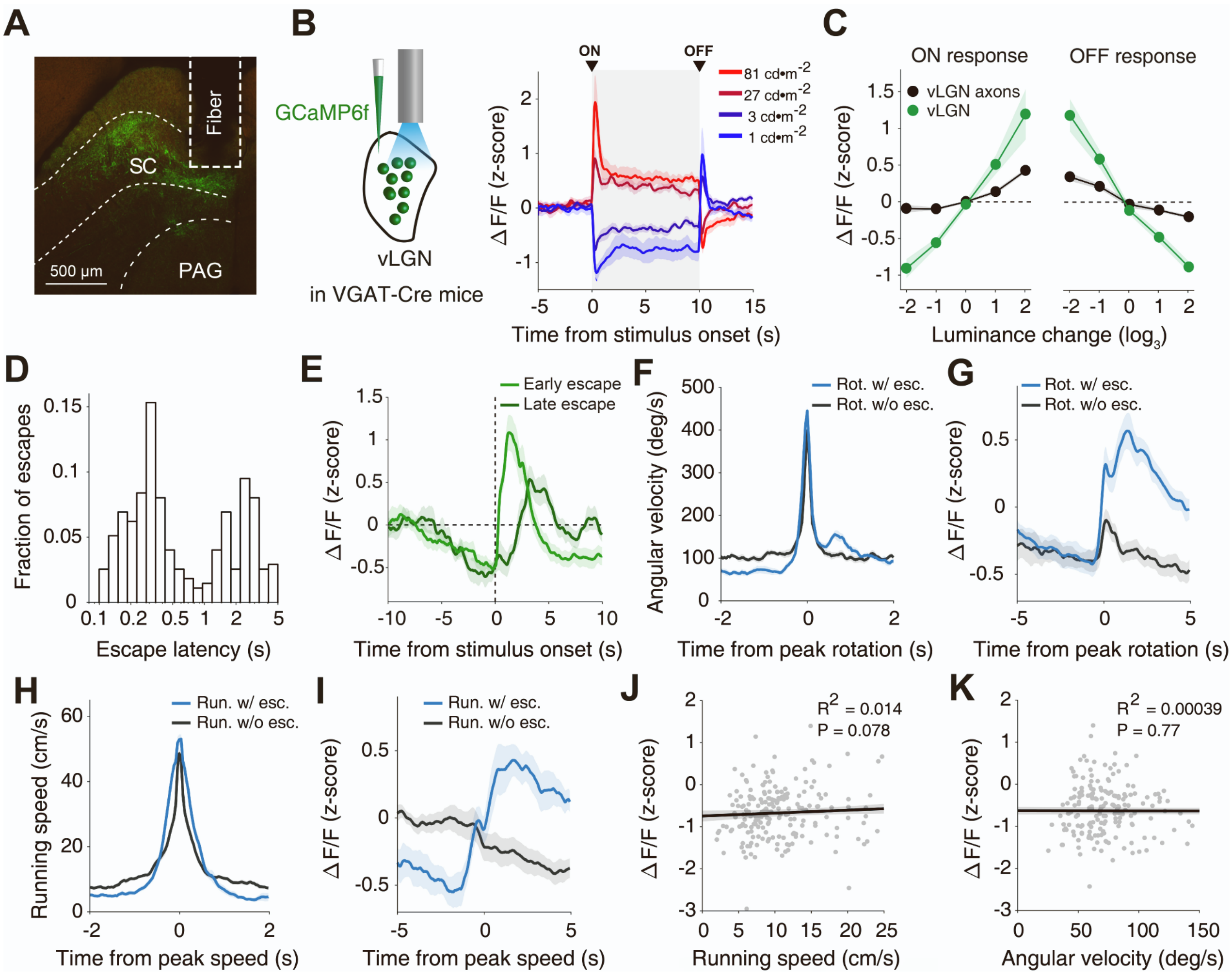

Supplemental Figure S3. Sensory and motor-related neuronal activity in vLGN and vLGN axons in mSC. Related to Figure 1.

(A) Fiber location and expression of GCaMP6f in GABAergic vLGN axons in mSC after injection of AAV-flex-GCaMP6f in VGAT-Cre mice. **(B)** Left: experimental paradigm for fiber photometry recordings of calcium signals in vLGN cell bodies after expression of GCaMP6f in vLGN GABAergic neurons. Right: mean vLGN calcium activity measured with optic fiber over vLGN in response to increases (27 cd×m^{-2}, magenta; 81 cd×m^{-2}, red) and decreases (3 cd×m^{-2}, indigo; 1 cd×m^{-2}, blue) in luminance from baseline levels (9 cd×m^{-2}). Stimulus duration is indicated by grey shading. Error bar shading represents SEM across mice; n = 6 mice. **(C)** Mean change in calcium activity due to the onset (ON response, left, difference in activity in the first second after stimulus onset compared to baseline) and the offset of the change in luminance (OFF response, right, difference in activity in the first second after stimulus offset and the last second of the stimulus) in vLGN (green, n = 6 mice) and in vLGN axons in mSC (black, n = 6 mice). Luminance change values are the base 3 logarithm of the ratio between stimulus and baseline luminance. Shading shows SEM across mice. **(D)** Distribution of time between stimulus onset and escape onset (escape latency) for escapes from looming stimuli; n = 274 trials, 9 mice. **(E)** Mean calcium activity of vLGN axons in mSC during threat-

evoked escapes aligned to stimulus onset, either with escape latency less than 0.5 seconds (early escape, light green, n = 9 mice), or more than 0.5 seconds (late escape, dark green, n = 7 mice). Shading shows SEM across mice. **(F)** Mean angular velocity in fast body rotations during escapes (blue) and in body rotations not part of an escape but with matched velocity (black) aligned to peak angular velocity. Shading shows SEM across mice; n = 9 mice. **(G)** Mean calcium activity of vLGN axons in mSC during the fast body rotations depicted in H that are part (blue) or not part (black) of an escape aligned to peak angular velocity. Shading shows SEM across mice; n = 9 mice. **(H)** Mean running speed during escape (blue) and during escape-unrelated bouts of fast running (black) aligned to peak running speed. Shading shows SEM across mice; n = 9 mice. **(I)** Mean calcium activity of vLGN axons in mSC during bouts of fast running speeds depicted in J, part (blue) or not part (black) of an escape aligned to peak running speed. Shading shows SEM across mice; n = 9 mice. **(J)** Relationship between the mean calcium activity and mean running speed of mice in the last two seconds of threat zone approach in experienced animals; n = 227 trials, 9 mice. R^2: coefficient of determination, P: p-value for slope different from 0 derived from a linear regression model. Shading around the line shows 95% confidence interval of the linear fit. **(K)** Relationship between mean calcium activity and mean angular velocity of mice in the last two seconds of threat zone approach in experienced animals; n = 227 trials, 9 mice. R^2: coefficient of determination, P: p-value for slope different from 0 derived from a linear regression model. Shading around the line shows 95% confidence interval of the linear fit.

Supplemental Figure S4

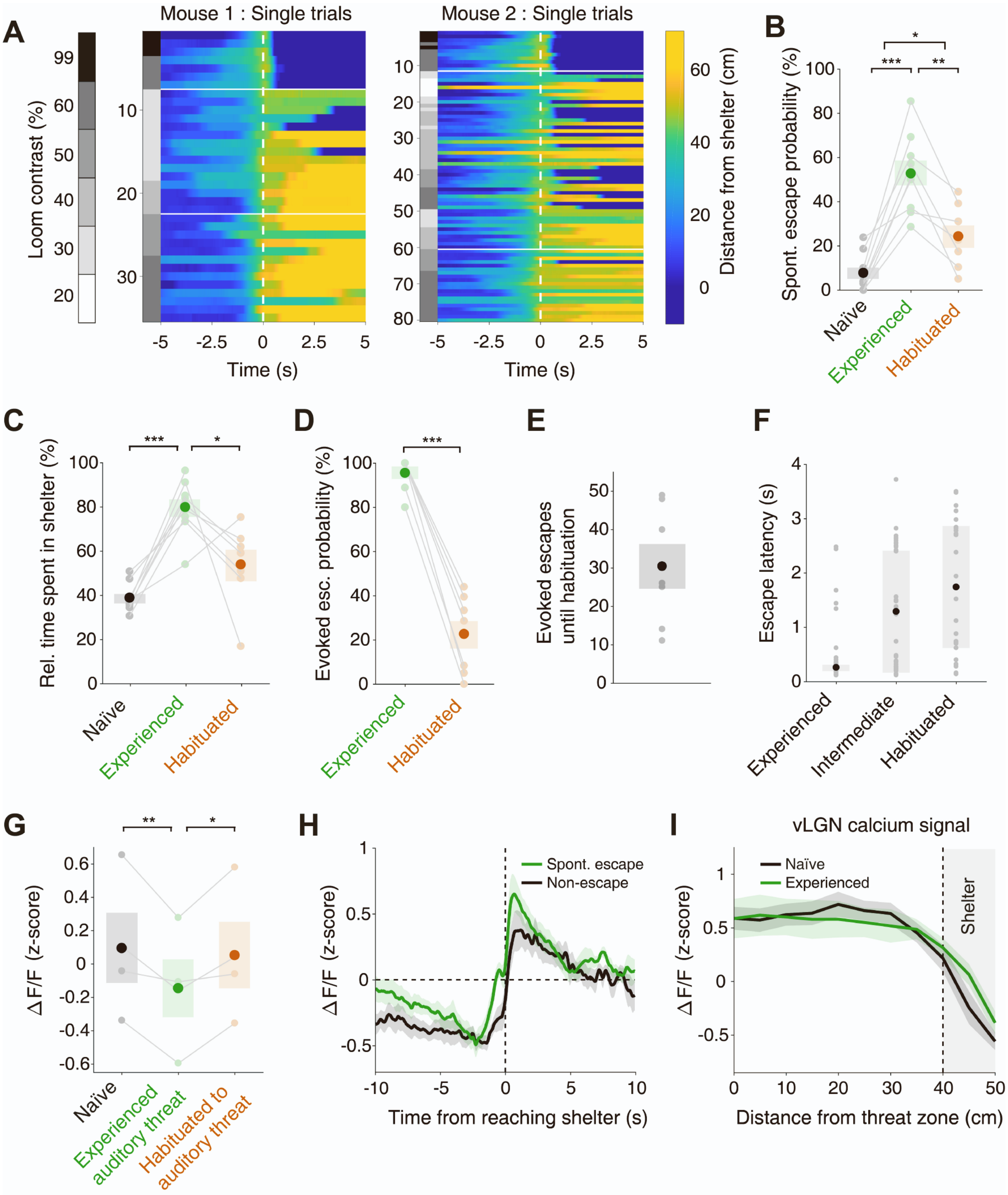

Supplemental Figure S4. Behavior and calcium signals during repeated exposure to threat stimuli. Related to Figure 1.

(A) Consecutive single trials of looming stimulus presentations of two example animals during photometry experiments, showing the mice's distance from the shelter (shelter position -10 to 0 cm) over time, aligned to stimulus onset (white dashed vertical line). Trials are shown in rows in chronological order from top to bottom. Stimulus contrast is indicated on the left of each trial. Top horizontal white line indicates the start of the habituation protocol; bottom horizontal white line indicates the timepoint after which the animal was considered habituated (when three consecutive trials of 50 - 60% contrast looming stimuli did not trigger an escape). **(B)** Mean spontaneous escape probability out of all returns to the shelter not triggered by a looming stimulus, in the period before presentation of the first looming stimulus (naïve; black, n = 9 mice), after presentation of the first looming stimulus (experienced; green, n = 9 mice), and after habituation (orange, n = 7 mice). Pale dots represent data from single animals. Error bars represent SEM across mice. Naïve - experienced: $p = 2.47\times10^{-6}$, experienced - habituated: $p = 1.13\times10^{-3}$, naïve - habituated: $p = 0.0269$. (Tukey's honest significance test preceded by repeated measures one-way analysis of variance, $p = 3.58\times10^{-6}$). **(C)** Mean relative time spent in the shelter in naïve (black, n = 9 mice), experienced (green, n = 9 mice), and habituated mice (orange, n = 7 mice). Pale dots represent data from single animals. Error bars represent SEM across mice. Naïve - experienced: $p = 8.63\times10^{-5}$, experienced - habituated: $p = 0.0145$, naïve - habituated: $p = 0.106$. (Tukey's honest significance test preceded by repeated measures one-way analysis of variance, $p = 1.22\times10^{-4}$). **(D)** Mean escape probability in response to intermediate-contrast looming stimuli (50% - 60%) in experienced (green) and habituated (orange) mice; n = 7 mice. Pale dots represent data from single animals. Error bars represent SEM across mice. $p = 7.05\times10^{-6}$, dependent t-test for paired samples. **(E)** Mean number of looming-stimulus evoked escapes before animals were considered habituated; n = 7 mice. Pale dots represent data from single animals. Error bars represent SEM across mice. **(F)** Median escape latency to intermediate-contrast (50% - 60%) looming stimuli in experienced animals (after the first looming stimulus, before the start of the habituation protocol, n = 47 escapes), during the habituation protocol (intermediate, n = 40 escapes) and after habituation (habituated, n = 30 escapes). Pale dots represent data from single escapes. Error bars represent IQR across escapes. **(G)** Calcium signals of vLGN axons in mSC in mice presented with auditory threat stimuli (loud 15 kHz tones, see Methods). Mean calcium activity in mice approaching the threat-zone before presentation of the first auditory threat stimulus (black, naïve), after presentation of the first auditory threat stimulus (green, experienced) and after habituation (orange). Pale dots represent data from single animals; n = 4 mice. Error bars represent the SEM across mice. Naïve – experienced: $p = 6.76\times10^{-3}$, experienced – habituated: $p = 0.017$, naïve – habituated: $p = 0.683$. (Tukey's honest significance test preceded by repeated measures one-way analysis of variance, $p = 6.07\times10^{-3}$). **(H)** Mean calcium activity in vLGN axons aligned to the moment at which mice reach the shelter following a spontaneous escape (green; initiated with a turn towards the shelter and reaching the shelter within 2.5 s) and after a slow, non-escape return to the shelter (black, speed < 40 $cm\times s^{-1}$ in the 2 s before reaching shelter). **(I)** Mean calcium activity in vLGN (measured with a fiber over vLGN) binned by distance during threat zone approach in naïve mice (black, n = 7 mice) and after presentation of the first looming stimulus (green, experienced, n = 7 mice). Shading shows SEM across mice. Dashed line represents the shelter location.

Supplemental Figure S5

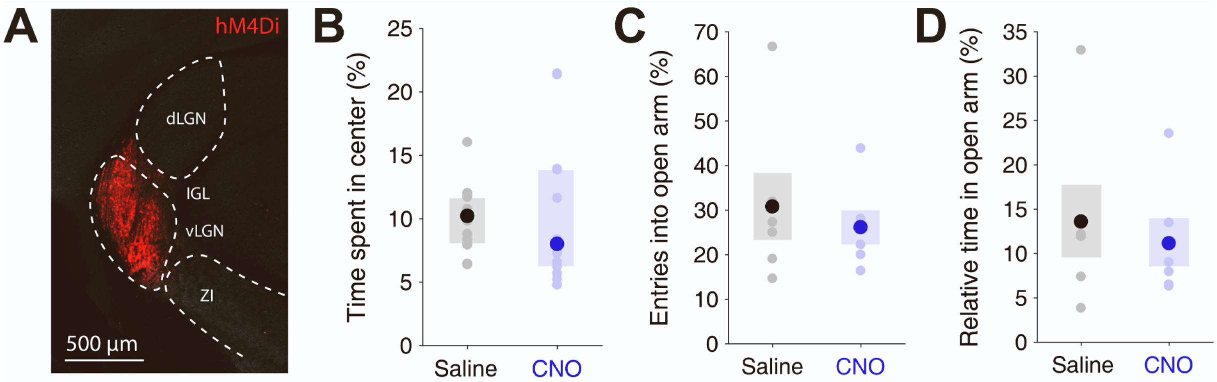

Supplemental Figure S5. hM4Di construct expression and control experiments testing effects of CNO. Related to Figure 2.

(A) Expression of hM4Di in vLGN GABAergic neurons in VGAT-Cre mice. **(B)** Median relative time spent in the center during 5 minutes in the open field test in systemic CNO- (blue, n = 14 mice) and saline-injected (black, n = 14 mice) wild-type control animals. Pale dots represent data from single animals. Error bars represent IQR across mice. p = 0.370, Wilcoxon rank-sum test. **(C)** Mean entries into open arms as a percentage of total arm entries during 15 minutes on the elevated plus maze in systemic CNO- (blue, n = 6 mice) and saline-injected (black, n = 6 mice) wild-type control animals. Pale dots represent data from single animals. Error bars represent SEM across mice. p = 0.599, independent two-sample t-test. **(D)** Mean relative time spent in open arms during 15 minutes on the elevated plus maze in CNO- (blue, n = 6 mice) and saline-injected (black, n = 6 mice) wild-type control animals. Pale dots represent data from single animals. Error bars represent SEM across mice. p = 0.632, independent two-sample t-test.

Supplemental Figure S6

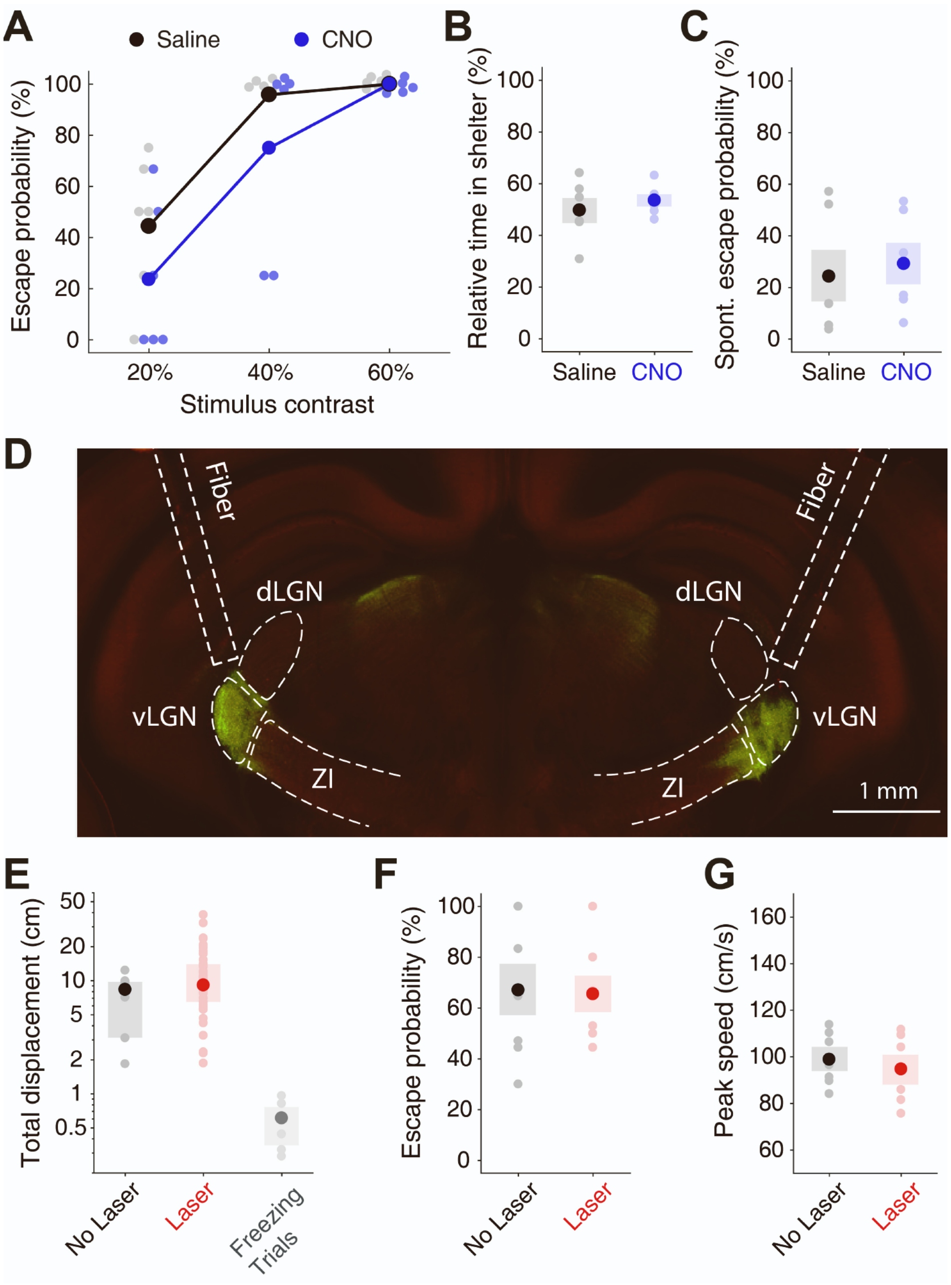

Supplemental Figure S6. Control experiments testing effects of CNO and laser. Related to Figure 3.

(A) Mean escape probability in response to looming stimuli of different contrasts for CNO- (blue, n = 6 mice) or saline-injected (black, n = 6 mice) wild-type control animals. Pale dots represent data from single animals. **(B)** Mean relative time spent in the shelter after exposure to the first looming stimulus for CNO- (blue, n = 6 mice) or saline-injected (black, n = 6 mice) wild-type control animals. Pale dots represent data from single animals. Error bars represent SEM across mice. p = 0.345, independent two-sample t-test. **(C)** Mean spontaneous escape probability after exposure to the first looming stimulus for CNO- (blue, n = 6 mice) or saline-injected (black, n = 6 mice) wild-type control animals.

Pale dots represent data from single animals. Error bars represent SEM across mice. $p = 0.639$, independent two-sample t-test. **(D)** Example image of bilateral ChR2 expression in vLGN GABAergic neurons after injection of AAV-flex-ChR2 in vGAT-Cre mice with indicated position of optic fibers. The fiber track on the left hemisphere is more evident in a more anterior slice. **(E)** Median change in the mouse's position during presentation of high-contrast looming stimuli in non-escape trials without optogenetic stimulation (black, n = 6 out of 47 trials), in non-escape trials with laser stimulation of ChR2-expressing vLGN neurons (red, n = 47 out of 47 trials), and in the few trials out of all optogenetic experiments in which freezing was observed in response to low- and intermediate-contrast looming stimuli (grey, n = 7 out of 266 trials). Pale dots represent data from single trials. Error bars represent IQR across trials. **(F)** Mean escape probability in response to looming stimuli without (black) and with laser stimulation (red) in all control mice with either expression of GFP in vLGN GABAergic neurons (n = 3 mice) or without fluorescent construct expression in vLGN (n = 4 mice). Pale dots represent data from single animals. Error bars represent SEM across mice. $p = 0.806$, dependent t-test for paired samples. **(G)** Mean peak running speed during escapes to looming stimuli without (black) and with laser stimulation (red) in control mice without expression of ChR2 in vLGN neurons; n = 7 mice. Pale dots represent data from single animals. Error bars represent SEM across mice. $p = 0.430$, dependent t-test for paired samples.

Supplemental Figure S7

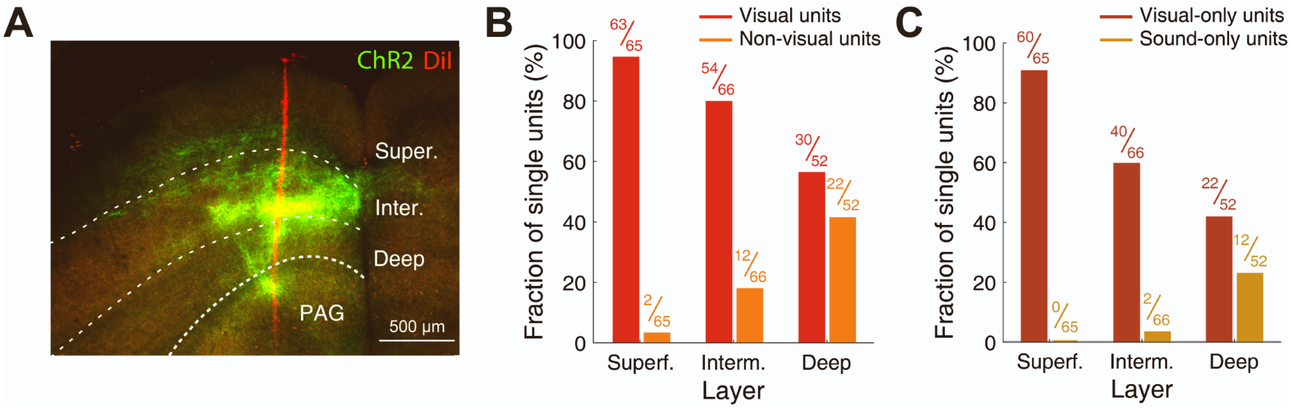

Supplemental Figure S7. In vivo response properties of mSC neurons in different layers. Related to Figure 4.

(A) Example image showing ChR2-labelled vLGN axons (green) in mSC and the placement of the Neuropixels probe (red DiI track) for extracellular electrophysiological recordings. mSC was divided into anatomical layers for analysis as indicated by dashed white lines; Super.: superficial layers (zonal, superficial grey and optic layers), Inter.: intermediate layers (intermediated grey and intermediate white layers); Deep: deep layers (deep grey and deep white layers). **(B)** Layer-wise distribution of single units responding to either looming or grating stimuli (visual, red), and of single units not responding to any visual stimulus (non-visual, orange). Single units from 11 recordings in 6 animals. **(C)** Layer-wise distribution of single units responding to either looming or grating stimuli, but not to sounds (visual-only, dark red), and of single units not responding to any visual stimulus but showing a significant response to sounds (sound-only, dark orange). Single units from 11 recordings in 6 animals.

Supplemental Figure S8

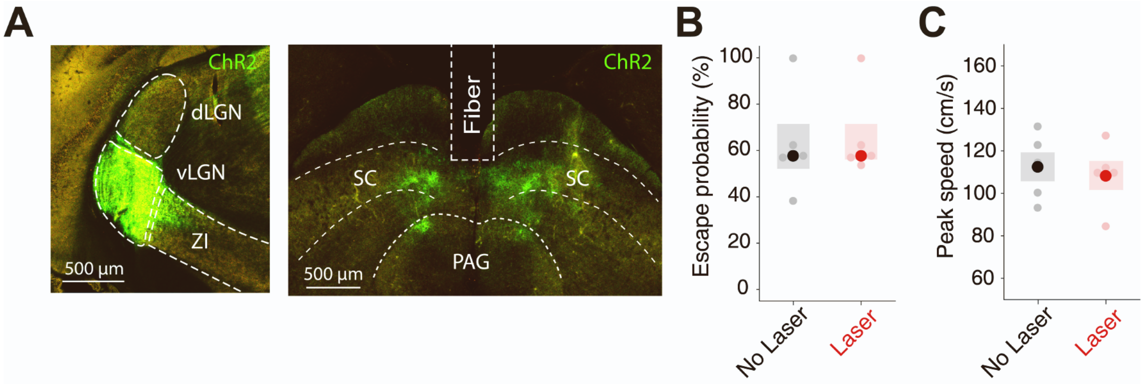

Supplemental Figure S8. ChR2 expression and control experiments to test for laser effect. Related to Figure 5.

(A) Example images of ChR2 expression in vLGN GABAergic neurons (left) and ChR2-labelled vLGN axons in SC with indicated optic fiber position (right). ChR2 was expressed bilaterally, one fiber was placed over SC at the midline. **(B)** Median escape probability in response to high-contrast looming stimuli without (black) and with laser stimulation in mSC (red) in control mice with either expression of GFP in vLGN neurons or without fluorescent construct expression in vLGN; n = 5 mice. Pale dots represent data from single animals. Error bars represent IQR across mice. p = 1.00, Wilcoxon signed-rank test. **(C)** Mean peak running speed during escapes to high-contrast looming stimuli with (black) and without laser stimulation in mSC (red) in control mice without expression of ChR2 in vLGN neurons, similar to B; n = 5 mice. Pale dots represent data from single animals. Error bars represent SEM across mice. p = 0.353, dependent t-test for paired samples.

Supplemental Figure S9

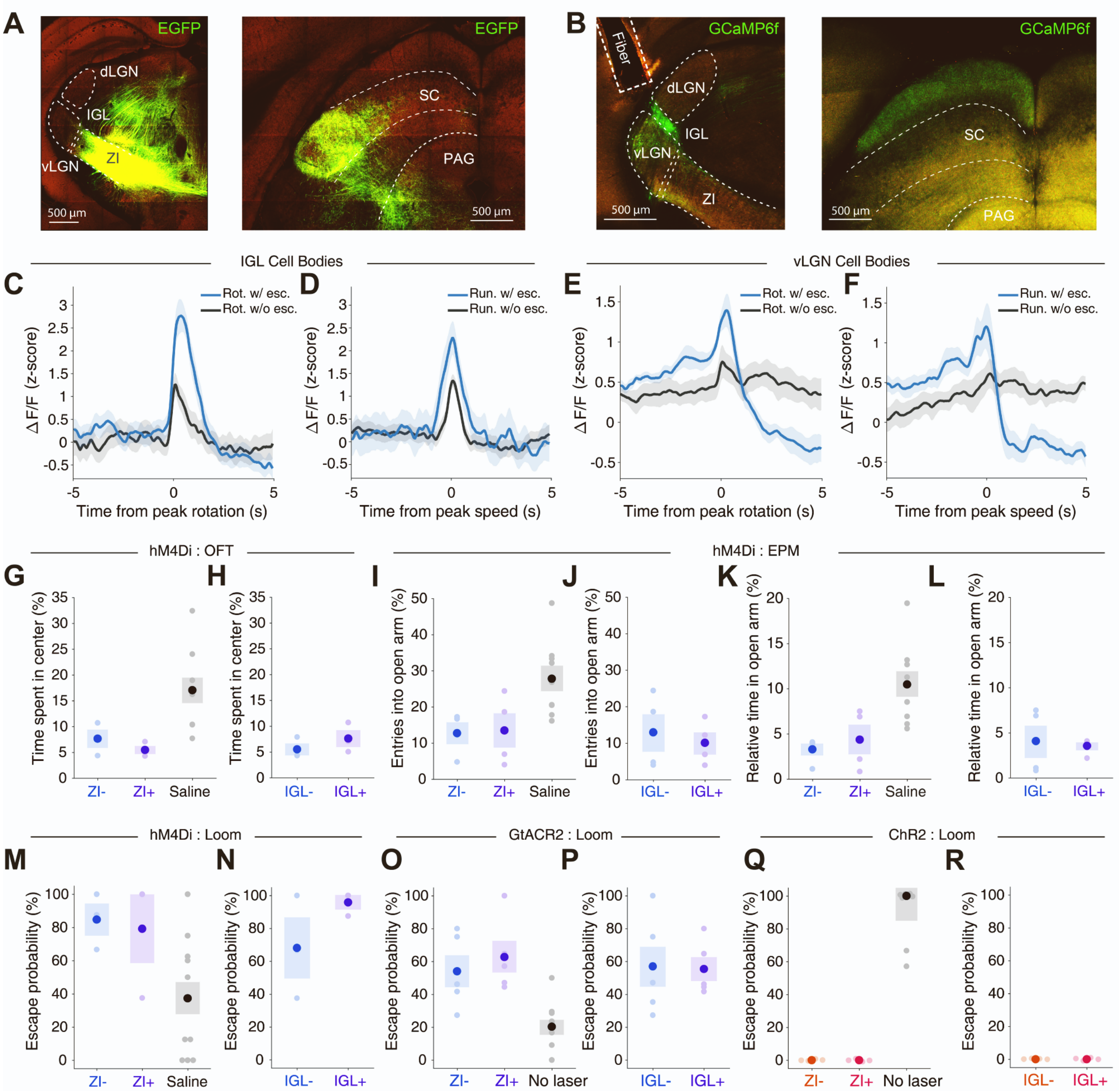

Supplemental Figure S9. Control analyses testing the involvement of IGL and ZI. Related to STAR Methods.

(A) Example images of EGFP expression in zona incerta (ZI) neurons in a WT animal from the Allen Mouse Brain Connectivity Atlas (left, Experiment 113095845) and corresponding ZI axons in lateral SC (right). **(B)** Example images of GCaMP6f expression in GABAergic intergeniculate leaflet (IGL) neurons with track indicating fiber position for photometry recordings (left) and corresponding GABAergic IGL axons in superficial layers in SC (right). **(C,D)** Mean calcium activity in IGL GABAergic neurons (GCaMP expression restricted to IGL, as depicted in **B**; fiber over IGL) during fast body rotations, aligned to peak angular velocity **(C)**, and during bouts of fast running speeds,

aligned to peak running speed **(D)**, in escape (blue) and non-escape (black) trials. Shading shows 95% confidence interval of the mean across trials; n = 1 mouse. **(E,F)** Mean calcium activity in vLGN GABAergic neurons (fiber over vLGN) during fast body rotations, aligned to peak angular velocity **(E)**, and bouts of fast running speeds, aligned to peak running speed **(F)**, in escape (blue) and non-escape (black) trials. Shading shows SEM across mice; n = 7 mice. **(G)** Mean relative time spent in the center during 5 minutes in the open field test of animals injected with CNO with the least hM4Di expression in Zona Incerta (ZI-, light blue, n = 3 mice), in animals injected with CNO with the most hM4Di expression in ZI (ZI+, purple, n = 3 mice, data divided in two halves here and in the following figure panels), and in saline-injected animals (black, n = 9 mice). Pale dots represent data from single animals. Error bars represent SEM across mice. ZI- vs ZI+ : p = 0.338, independent two-sample t-test. **(H)** Same as **G** but in animals injected with CNO with the least hM4Di expression in the intergeniculate leaflet (IGL-, light blue, n = 3 mice), and in animals injected with CNO with the most hM4Di expression in IGL (IGL+, purple, n = 3 mice). p = 0.363, independent two-sample t-test. **(I)** Mean entries into open arms as a percentage of total arm entries during 15 minutes on the elevated plus maze in animals injected with CNO with the least hM4Di expression in ZI (ZI-, light blue, n = 4 mice), in animals injected with CNO with the most hM4Di expression in ZI (ZI+, purple, n = 4 mice), and in saline-injected animals (black, n = 9 mice). Pale dots represent data from single animals. Error bars represent SEM across mice. ZI- - ZI+ : p = 0.898, independent two-sample t-test. **(J)** Same as **I** but in animals injected with CNO with the least hM4Di expression in IGL (IGL-, light blue, n = 4 mice), and in animals injected with CNO with the most hM4Di expression in IGL (IGL+, purple, n = 4 mice). p = 0.643, independent two-sample t-test. **(K)** Mean relative time spent in open arms during 15 minutes on the elevated plus maze in animals injected with CNO with the least hM4Di expression in ZI (ZI-, light blue, n = 4 mice), in animals injected with CNO with the most hM4Di expression in ZI (ZI+, purple, n = 4 mice), and in saline-injected animals (black, n = 9 mice). Pale dots represent data from single animals. Error bars represent SEM across mice. ZI- - ZI+ : p = 0.578, independent two-sample t-test. **(L)** Same as **K** but in animals injected with CNO with the least hM4Di expression in IGL (IGL-, light blue, n = 4 mice), and in animals injected with CNO with the most hM4Di expression in IGL (IGL+, purple, n = 4 mice). p = 0.786, independent two-sample t-test. **(M)** Mean escape probability in response to low contrast stimuli (20% and 40%) in animals injected with CNO with the least hM4Di expression in ZI (ZI-, light blue, n = 3 mice), in animals injected with CNO with the most hM4Di expression in ZI (ZI+, purple, n = 3 mice), and in saline-injected animals (black, n = 12 mice). Pale dots represent data from single animals. Error bars represent SEM across mice. ZI- - ZI+ : p = 0.866, independent two-sample t-test. **(N)** Same as **M,** but in animals injected with CNO with the least hM4Di expression in IGL (IGL-, light blue, n = 3 mice), and in animals injected with CNO with the most hM4Di expression in IGL (IGL+, purple, n = 3 mice). p = 0.331, independent two-sample t-test. **(O)** Mean escape probability in response to low-contrast looming stimuli (30% - 40%) in bilateral vLGN inhibition trials in animals with the least GtACR2 expression in ZI (ZI-, light blue, n = 5 mice), in animals with the most GtACR2 expression in ZI (ZI+, purple, n = 5 mice), and in control trials (black, n = 10 mice). Pale dots represent data from single animals. Error bars represent SEM across mice. ZI- - ZI+ : p = 0.56, independent two-sample t-test. **(P)** Same as **O**, but in animals with the least GtACR2 expression in IGL (IGL-, light blue, n = 5 mice) and in animals with the most GtACR2 expression in IGL (IGL+, purple, n = 5 mice). p = 0.912, independent two-sample t-test. **(Q)** Median escape probability in response to high-contrast looming stimuli (99%) in bilateral

vLGN stimulation trials in animals with the least ChR2 expression in ZI (ZI-, orange, n = 4 mice), in animals with the most ChR2 expression in ZI (ZI+, pink, n = 4 mice) and in control trials (black, n = 8 mice). Pale dots represent data from single animals. Error bars represent IQR across mice. ZI- - ZI+ : p = 1.00, Wilcoxon rank-sum test. **(R)** Same as **Q**, but in animals with the least ChR2 expression in IGL (IGL-, orange, n = 4 mice) and in animals with the most ChR2 expression in IGL (IGL+, pink, n = 4 mice). p = 1.00, Wilcoxon rank-sum test.

Supplemental Figure S10

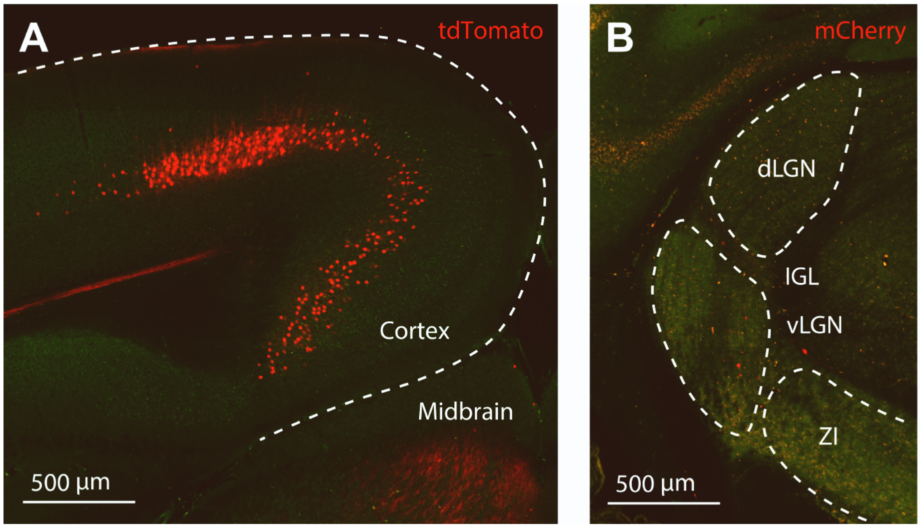

Supplemental Figure S10. Non-efficient retrograde transport of retroAAV in vLGN projections to mSC. Related to STAR Methods.

(A) Cre-dependent tdTomato expression in cortical cells after the injection of AAVretro-hSyn-Cre in the medial superior colliculus in a tdTomato-reporter mouse (Ai14D). **(B)** Very sparse Cre-dependent expression of mCherry in vLGN after the injection of AAVretro-hSyn-Cre in the medial superior colliculus and a large injection of AAV1-EF1a-FLEX-mCherry in the vLGN of a WT mouse (C57BL/6J).
