# Supplementary material for: Flexible inhibitory control of visually evoked defensive behavior by the ventral lateral geniculate nucleus
Source: Neuron. 2021 Dec 1;109(23):3810–3822.e9. doi: 10.1016/j.neuron.2021.09.003 (PMC8648186; doi:10.1016/j.neuron.2021.09.003)
Supplement: Document S2. Article plus supplemental information [file mmc6.pdf]

Neuron

Article

Flexible inhibitory control of visually evoked defensive behavior by the ventral lateral geniculate nucleus

Graphical abstract

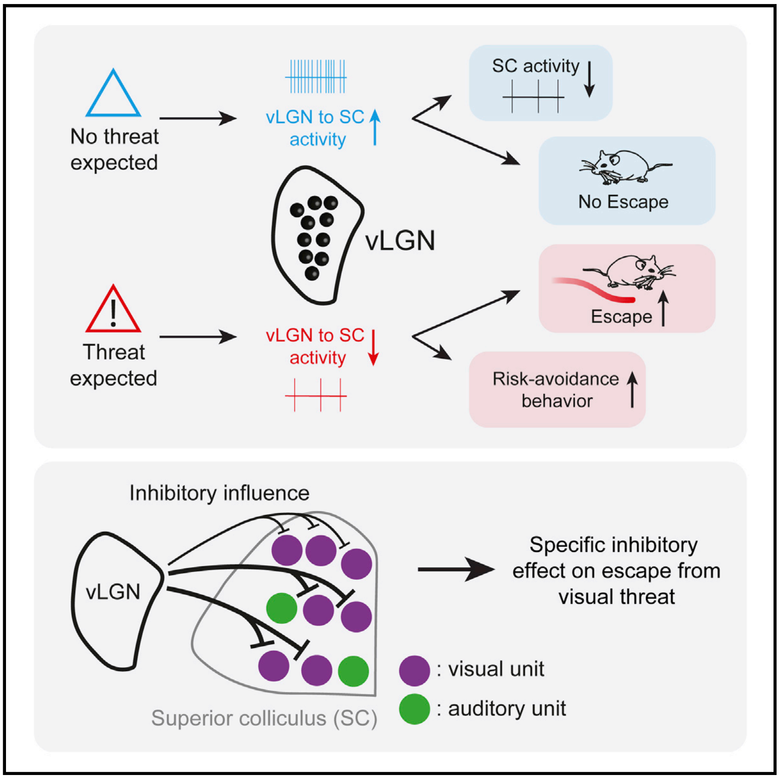

Highlights

- Activity of vLGN axons in the mSC reflects the previous experience of threat
- The vLGN bidirectionally controls escape from visual threat
- Activating the vLGN specifically reduces the activity of visual units in mSC
- Activating vLGN axons in the mSC specifically suppresses escape from visual threat

Authors

Alex Fratzl, Alice M. Koltchev, Nicole Vissers, ..., A. Vanessa Stempel, Tiago Branco, Sonja B. Hofer

Correspondence

s.hofer@ucl.ac.uk

In brief

Animals can act upon or ignore stimuli, depending on their prior knowledge. Here, Fratzl et al. identify the vLGN as critical for the control of defensive responses evoked by visual stimuli in mice. vLGN activity reflects previous experience of threat, and this nucleus, thus, regulates escape from visual threat depending on the animal's knowledge.

Fratzl et al., 2021, Neuron *109*, 3810–3822
December 1, 2021 © 2021 The Author(s). Published by Elsevier Inc.
https://doi.org/10.1016/j.neuron.2021.09.003

CellPress

CellPress
OPEN ACCESS

Neuron

Article

Flexible inhibitory control of visually evoked defensive behavior by the ventral lateral geniculate nucleus

Alex Fratzl,[1] Alice M. Koltchev,[1] Nicole Vissers,[1] Yu Lin Tan,[1] Andre Marques-Smith,[1] A. Vanessa Stempel,[1] Tiago Branco,[1] and Sonja B. Hofer[1,2,*]
[1]Sainsbury Wellcome Centre for Neural Circuits and Behaviour, University College London, London, UK
[2]Lead contact
*Correspondence: s.hofer@ucl.ac.uk
https://doi.org/10.1016/j.neuron.2021.09.003

SUMMARY

Animals can choose to act upon, or to ignore, sensory stimuli, depending on circumstance and prior knowledge. This flexibility is thought to depend on neural inhibition, through suppression of inappropriate and disinhibition of appropriate actions. Here, we identified the ventral lateral geniculate nucleus (vLGN), an inhibitory prethalamic area, as a critical node for control of visually evoked defensive responses in mice. The activity of vLGN projections to the medial superior colliculus (mSC) is modulated by previous experience of threatening stimuli, tracks the perceived threat level in the environment, and is low prior to escape from a visual threat. Optogenetic stimulation of the vLGN abolishes escape responses, and suppressing its activity lowers the threshold for escape and increases risk-avoidance behavior. The vLGN most strongly affects visual threat responses, potentially via modality-specific inhibition of mSC circuits. Thus, inhibitory vLGN circuits control defensive behavior, depending on an animal's prior experience and its anticipation of danger in the environment.

INTRODUCTION

Instinctive defensive behaviors are vital for survival because they enable fast reactions to environmental threats, such as escape from an approaching predator (Blanchard et al., 1990; Fotowat and Gabbiani, 2011; Yilmaz and Meister, 2013; Peek and Card, 2016; Branco and Redgrave, 2020). However, these instinctive responses are surprisingly flexible and can be adapted or suppressed depending on environmental demands, the animal's state, and prior knowledge (Evans et al., 2019). How such flexible control of behavior is implemented in the brain is not well understood. Neural inhibition is thought to have an important role in suppressing inappropriate actions and in enabling the selection and initiation of appropriate responses through disinhibition. Although previous work has largely focused on inhibitory pathways in the basal ganglia (Mink, 1996; Nelson and Kreitzer, 2014), recent evidence suggests that inhibitory structures in the prethalamus, such as the zona incerta (ZI), could assert more direct control over responses to environmental stimuli (Chou et al., 2018; Zhao et al., 2019; Wang et al., 2019; Venkataraman et al., 2019; Hormigo et al., 2020; Ahmadlou et al., 2021).

Vision is crucial for detecting approaching environmental threats. For instance, rapidly expanding dark overhead (''looming'') spots that mimic aerial predators are innately threatening and trigger fast, defensive responses in a large variety of animals, including rodents and primates (Schiff et al., 1962; Fotowat and Gabbiani, 2011; Yilmaz and Meister, 2013; Temizer et al., 2015; De Franceschi et al., 2016; Evans et al., 2018; Shang et al., 2018; Branco and Redgrave, 2020). Key neuronal circuits underlying such visually evoked defensive behavior have been localized to the midbrain (Wei et al., 2015; Dunn et al., 2016; Evans et al., 2018; Shang et al., 2018; Branco and Redgrave, 2020). However, it is unclear how these circuits are regulated to enable flexible control of defensive behavior.

The ventral lateral geniculate nucleus (vLGN)—or pregeniculate nucleus in primates—resides ventral to the better-known thalamic dorsal LGN in rodents. The vLGN is a prethalamic nucleus that contains a large fraction of GABAergic neurons and receives prominent projections from the retina and visual cortex (Harrington, 1997; Oh et al., 2014; Monavarfeshani et al., 2017; Nakagawa, 2019; Sabbagh et al., 2020). It has recently been shown to mediate effects of light therapy on depressive symptoms and spatial memory (Huang et al., 2019, 2021) but has also been suggested to have a role in widely differing visual functions, such as ocular-motor processing and the regulation of circadian rhythms (Magnin et al., 1974; Harrington, 1997; Livingston and Fedder, 2003), leaving its function unresolved. However, prominent vLGN projections to several brain regions that are crucial for the execution of instinctive defensive reactions, in particular, the superior colliculus (SC) and periaqueductal gray (PAG) in the midbrain (Oh et al., 2014; Evans et al., 2018; Branco and Redgrave, 2020; Lefler et al., 2020), point to a role of this nucleus in regulating visually evoked, defensive behavior.

This is an open access article under the CC BY license (http://creativecommons.org/licenses/by/4.0/).

CellPress
OPEN ACCESS

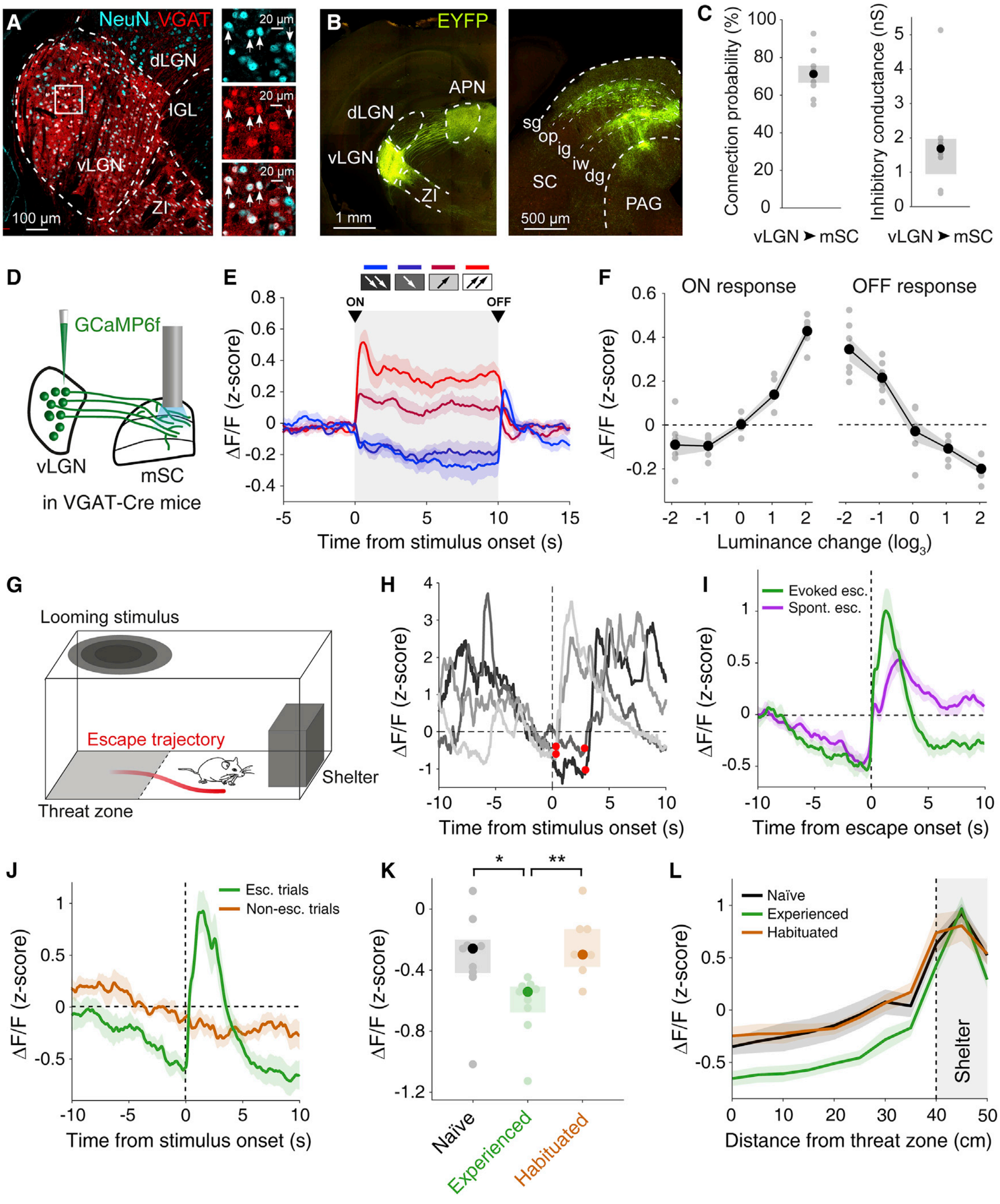

Figure 1. Activity of vLGN axons in mSC reflects previous experience of threat

(A) Example images of tdTomato expression in VGAT+ neurons in the vLGN (red), combined with NeuN staining (cyan). Images on the right are from the inset in the left image, showing only NeuN staining (top), tdTomato expression (middle), and both combined (bottom). Arrows indicate examples of NeuN+ neurons.

(B) Left, expression of EYFP in the vLGN after injection of AAV-flex-EYFP in VGAT-Cre mice. Right, GABAergic vLGN axons in the SC and PAG. APN, anterior pretectal nucleus; dLGN, dorsal lateral geniculate nucleus; IGL, intergeniculate leaflet; PAG, periaqueductal gray; SC, superior colliculus; vLGN, ventral lateral geniculate nucleus; ZI, zona incerta. SC layers: sg, superficial gray layer; op, optical layer; ig, intermediate gray layer; iw, intermediate white layer; dg, deep gray layer.

(C) Left, mean connection probability between GABAergic vLGN axons and cells in the medial SC (mSC), observed using ChR2-assisted circuit mapping *in vitro*. Error bars represent standard errors of the mean (SEM) across mice. Right, median inhibitory conductance in mSC cells in response to stimulation of GABAergic vLGN axons. Error bars represent the interquartile range (IQR) across mice. Grey dots represent data from single animals; n = 8 mice, 81 cells.

(legend continued on next page)

We, therefore, tested the influence of the vLGN over instinctive defensive reactions and found that it exerts strong, bidirectional control over escape responses to visual threat. The activity of GABAergic vLGN axons in the SC reflects the animal's experience and its anticipation of environmental threat. We show that high activity in vLGN GABAergic neurons prevents escape, whereas low activity increases risk-avoidance behavior and promotes escape responses. The vLGN has a stronger effect on responses to visual, than to auditory, threats, and we find that this modality-specific effect on behavior is likely supported by a prominent, modality-specific influence of the vLGN on medial SC (mSC) circuits. Our data indicate that the vLGN regulates defensive behavior depending on the animal's experience and assessment of the environment.

RESULTS

vLGN is an inhibitory prethalamic nucleus prominently innervating the mSC

The vLGN is a retinorecipient nucleus in the prethalamus that has been reported to contain a substantial fraction of GABAergic neurons (Harrington, 1997; Monavarfeshani et al., 2017; Langel et al., 2018; Nakagawa, 2019; Sabbagh et al., 2020). We quantified the proportion of GABAergic neurons within vLGN using VGAT::tdTomato mice and found that 85.1% ± 4.0% of vLGN neurons are GABAergic (Figures 1A, S1A, and S1B). To determine the projection targets of GABAergic vLGN neurons, we injected adeno-associated virus (AAV) encoding Cre-dependent EYFP into the vLGN of VGAT-Cre mice. $VGAT^{+}$ neurons in vLGN project to various regions, including the pretectum, the thalamic lateral posterior nucleus, and motor nuclei in the mid- and hindbrain (Figures S1C–S1F). Notably, the vLGN sends prominent GABAergic projections to all layers of mSC (Figure 1B). Whole-cell recordings of SC neurons in acute brain slices during optogenetic activation of GABAergic vLGN axons expressing channelrhodopsin-2 (ChR2) (Petreanu et al., 2007) showed that vLGN projections form inhibitory synaptic connections with a large proportion of excitatory and inhibitory neurons throughout the mSC (Figures 1C and S2). Given this dense connectivity, we set out to determine what information the vLGN conveys to mSC.

Activity of vLGN axons in mSC is low before escape from visual threat

To characterize the activity of vLGN inputs to mSC, we recorded GCaMP6f calcium signals of GABAergic vLGN axons in mSC using fiber photometry in freely-moving animals (Chen et al., 2013; Cui et al., 2013; Shang et al., 2018; Akam and Walton, 2019) (Figures 1D–1L and S3A). First, we measured visual responses of vLGN axons by presenting full-field light stimuli of different luminance levels on a screen above freely moving mice. vLGN axons in mSC showed, on average, a slight increase in activity with increasing levels of luminance (Figures 1E and 1F). The vLGN, thus, conveys visual information to mSC; however, these visually evoked signals were much weaker than the average responses of vLGN neurons to the same stimuli, as measured with a fiber above the vLGN (Figures S3B and S3C).

The mSC has been shown to be crucial for instinctive defensive reactions to visual threat (Wei et al., 2015; Evans et al., 2018; Shang et al., 2018; Branco and Redgrave, 2020). Therefore, we next characterized activity of vLGN axons in mSC when mice were exposed to innately aversive visual stimuli (Yilmaz and Meister, 2013; Branco and Redgrave, 2020). We placed mice in an arena with a shelter and, after a 30-min period of initial exploration, exposed them to overhead dark, expanding "looming" spots, presented only in a "threat" zone, located 40 cm from the shelter (Figure 1G) (Evans et al., 2018). Three consecutive high-contrast looming spots in a 3-s period reliably triggered a directed escape to the shelter (mean escape probability 97.8% ± 2.22%; Video S1; see also Figures 3 and 5). In individual escape trials, the activity of vLGN axons in mSC showed clear signals related to the threat-evoked escape (Figure 1H). Activity was low immediately before the looming stimulus and sharply increased after its onset (Figures 1H and 1I). Because decreases in luminance reduced activity of vLGN axons (Figures 1E and 1F), this sharp rise in activity was likely not evoked by the dark, looming stimulus but, instead, related to the mouse's escape. Indeed, upon separate analysis of trials in which mice initiated an escape

(D) Experimental paradigm for fiber photometry recordings of calcium signals from GABAergic vLGN axons in the mSC.

(E) Mean calcium activity of vLGN axons in the mSC in response to increases (27 cd $\times$ m^{-2}, magenta; 81 cd $\times$ m^{-2}, red) and decreases (3 cd $\times$ m^{-2}, indigo; 1 cd $\times$ m^{-2}, blue) in luminance from baseline levels (9 cd $\times$ m^{-2}). Stimulus duration is indicated by gray shading. Error-bar shading represents SEM across mice; n = 6 mice.

(F) Mean change in calcium activity due to the onset (ON response, left; see Method details) and the offset (OFF response, right) of the change in luminance. Luminance change values are the base-3 logarithm of the ratio between the stimulus and baseline luminance. Grey dots represent data points from individual mice. Shading shows SEM across mice; n = 6 mice.

(G) Schematic of the experimental approach. Red line denotes escape trajectory.

(H) Four representative single-trial calcium traces of vLGN axons in the mSC during threat-evoked escape aligned to stimulus onset. Red dots, escape onset.

(I) Mean calcium activity of vLGN axons in the mSC during threat-evoked (green) and spontaneous (purple) escapes aligned to escape onset. Shading shows SEM across mice; n = 9 mice.

(J) Mean calcium activity of vLGN axons recorded in the mSC in escape trials early in the recording session, before the start of the habituation protocol (green, n = 9 mice), and during non-escape trials in habituated animals (orange, n = 7 mice), aligned to stimulus onset. Shading shows SEM across mice.

(K) Median calcium activity of vLGN axons in mice approaching the threat zone before presentation of the first looming stimulus (black, naive, n = 9 mice), after presentation of the first looming stimulus (green, experienced, n = 9 mice), and after habituation (orange, n = 7 mice). Pale dots represent data from single animals. Error bars represent the IQR across mice. Naive-experienced: p = 0.0130, experienced-habituated: p = 8.31 $\times$ 10^{-3}, naive-habituated: p = 0.983, Dunn's multiple comparison test, preceded by Kruskal-Wallis one-way analysis of variance, p = 2.96 $\times$ 10^{-3}.

(L) Mean calcium activity of vLGN axons binned by distance during the 30 s before reaching the threat zone in naive mice (black, n = 9 mice), after presentation of the first looming stimulus (green, experienced, n = 9 mice), and after habituation (orange, n = 7 mice). Shading shows SEM across mice. Dashed line and gray shading indicate the location of the shelter.

within or later than 0.5 s after stimulus onset (Figure S3D), the increase in vLGN activity was shifted in time in late-escape trials and was tightly coupled to escape initiation, rather than stimulus onset (Figures 1I and S3E). Moreover, it was absent when mice did not escape in response to the looming stimulus (Figure 1J). The escape-related rise in activity was not caused by increased locomotion and could only to a small extent be explained by head-rotation movements (Figures S3F–S3I). A large part of the activity increase after escape onset can, however, be accounted for by signals related to entry into the shelter (Figure S4H; see also Figure 1L and below for detailed explanation).

Notably, vLGN axon activity consistently decreased in the few seconds before the appearance of the looming stimulus and reached a minimum at the time of escape initiation. This reduction in activity was apparent in single trials as well as in the average trace (Figures 1H and 1I). vLGN axon activity was similarly low before escapes not triggered by a looming stimulus, at times when animals fled back to the shelter spontaneously (Figure 1I). Escape-preceding decreases in activity could not be explained by changes in locomotion or head-rotation speed (Figures S3J and S3K). These results suggest that a decrease in vLGN activity may promote escape. To ascertain whether increased activity could, therefore, be indicative of a failure to escape, we compared trials in which mice escaped to the looming stimulus with trials in which they did not escape to the same stimulus (non-escape trials occurred toward the end of a recording session after repeated exposure to the looming stimulus; see below). vLGN axon activity just before stimulus onset was significantly greater in trials in which mice did not escape (Figure 1J; $p = 1.84 \times 10^{-3}$, paired t test). These data suggest that the elevated activity of vLGN projections in mSC may be related to the absence of escape responses. Moreover, the modulation of the vLGN axon activity before the visual threat stimulus, as well as before spontaneous escape bouts in the absence of threatening stimuli, suggests that vLGN axons convey a non-visual signal to the mSC that may be related to the behavioral state of the animal. To further explore that hypothesis, we investigated how escape responses and vLGN calcium signals change in different behavioral contexts.

Activity of vLGN axons in mSC is modulated by experience of threat

Previous experience of threatening stimuli can alter the general state of the animal, as well as its response to threats (Evans et al., 2019). After exposure to high-contrast looming stimuli, mice showed signs of increased anxiety when exploring the arena: they spent more time in the shelter and were more likely to abort approaches into the threat zone and to spontaneously flee back to the shelter (Figures S4B–S4C). Conversely, after repeated exposure to a series of low-contrast looming stimuli that have been shown to have low threat saliency and often do not trigger an escape (Figure S4A; Evans et al., 2018), mice showed signs of habituation: they were less likely to escape from subsequent looming stimuli, showed longer escape latencies, had fewer spontaneous escapes, and spent less time in the shelter (Figures S4A–S4F). The activity of vLGN axons in mSC reflected these changes in behavior. After mice had experienced a high-contrast looming stimulus in the threat zone, average vLGN axon activity during exploration of the arena was significantly reduced compared with that of naive animals that had not yet experienced a threatening stimulus (Figure 1K; p = 0.0130, Dunn's multiple comparison test). In contrast, when mice started to habituate to looming stimuli after repeated exposure to low-contrast looming stimuli, vLGN axon activity in the arena increased, on average, to levels comparable with those observed in naive animals (Figures 1K and 1L; experienced-habituated: $p = 8.31 \times 10^{-3}$, naive-habituated: p = 0.983, Dunn's multiple comparison test). vLGN signals in mSC were thus strongly modulated by the animal's previous experience of threat. We observed similar changes in vLGN axon activity after mice were exposed to auditory threat stimuli (Figure S4G), indicating that this experience-dependent modulation of vLGN signals to the mSC is independent of threat modality.

Moreover, vLGN axon activity was related to the level of danger anticipated by the animal: in all behavioral conditions, activity was by far greatest when mice were in the shelter (Figures 1L and S4H), a safe place to which animals regularly returned, even in the absence of imminent threat. Further, calcium activity was correlated with the mouse's distance to the shelter ($p = 7.24 \times 10^{-4}$, $R^2 = 0.736$, linear regression model) and was lowest in the threat zone in mice that were previously exposed to high-contrast looms in that area (Figure 1L). This experience- and location-dependent modulation of vLGN axon signals fully explains the low activity around the time the looming stimulus is encountered in the threat zone (Figures 1H and 1I) and the higher level of activity at the same time point in habituated animals that did not escape (Figure 1J). Interestingly, experience-dependent changes in activity were absent in the average GABAergic population within the vLGN itself, which seemed to mainly signal luminance changes in the environment (for instance between the shelter and the arena; Figure S4I). These results indicate that, specifically, the activity of vLGN projections to the mSC reflects an animal's level of anxiety given its experience. However, these experiments could not establish whether vLGN activity is causally related to such state-dependent modulation of behavior. Accordingly, we next manipulated activity of GABAergic vLGN neurons during paradigms traditionally used to assess anxiety-related behavior in mice: the open field test and the elevated plus maze.

vLGN suppression increases risk-avoidance behavior

Rodents show a strong aversion to open, exposed spaces. Therefore, the amount of time mice spend in the center of the open field, away from the walls, or in the open, unsheltered arms of the elevated plus maze, is used to measure their level of anxiety (Crawley, 1985; Pellow et al., 1985). We bilaterally expressed hM4Di in vLGN GABAergic neurons via AAV injections (Armbruster et al., 2007; Krashes et al., 2011) (Figures 2A and S5A). Neuronal activity and synaptic transmission of hM4Di-expressing neurons can be suppressed by the agonist clozapine N-oxide (CNO; Roth, 2016). After systemic injections of CNO ($5 mg \times kg^{-1}$ intraperitoneal [i.p.]), mice spent significantly less time in the center of the open-field arena than did animals injected with saline (Figures 2A and 2B; mean time in center, saline: 17.0% ± 2.54%, CNO: 6.55% ± 1.03%, $p = 6.75 \times 10^{-3}$, independent two-sample t test). Similarly, mice expressing hM4Di in

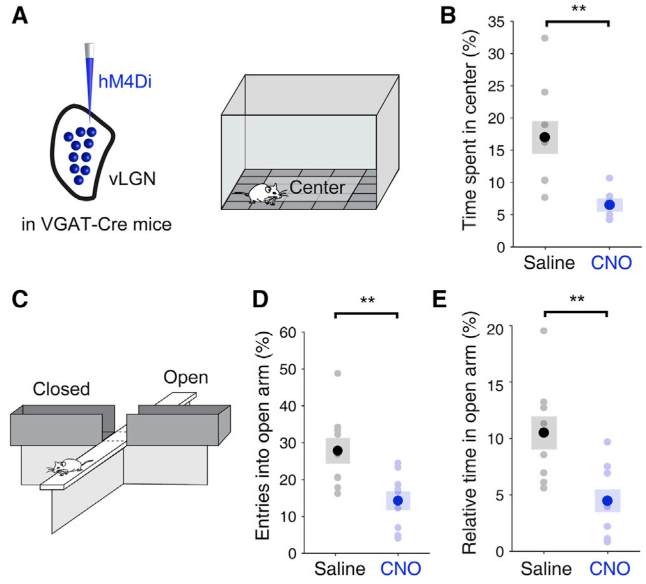

Figure 2. vLGN suppression increases risk-avoidance behavior

(A) Experimental approach: open field test after expression of hM4Di in GABAergic vLGN neurons.

(B) Mean relative time spent in the center during 5 min in the open field arena in systemic CNO- (blue, n = 6 mice) and saline-injected (black, n = 9 mice) animals. p = 6.75 × 10^{-3}, independent two-sample t test.

(C) Schematic of the elevated plus maze.

(D) Mean entries into open arms as a percentage of total arm entries during 15 min on the maze in systemic CNO- (blue, n = 9 mice) and saline-injected (black, n = 9 mice) animals. p = 6.29 × 10^{-3}, independent two-sample t test.

(E) Mean relative time spent in open arms during 15 min on the elevated plus maze. p = 3.71 × 10^{-3}, independent two-sample t test. In all plots, pale dots represent data from single animals. Error bars represent SEM across mice.

vLGN GABAergic neurons entered the open arm of the elevated plus maze less frequently and spent less time there after CNO injection (Figures 2C–2E; mean time in open arm, saline: 10.5% ± 1.46%, CNO: 4.47% ± 1.00%, p = 3.71 × 10^{-3}, independent two-sample t test). CNO injections in control animals without hM4Di expression had no effect on behavior in these assays (Figures S5B–S5D). Therefore, suppression of vLGN inhibitory activity increases anxiety-related risk-avoidance behavior and suppresses exploration of exposed environments. Next, we wanted to test how vLGN activity affects animals' responses to imminent threats.

vLGN bidirectionally controls escape from imminent visual threat

We observed a robust difference in vLGN axon activity at the time at which mice decided whether to escape from a looming stimulus or not (Figure 1J). This suggests that vLGN may regulate the behavioral response to this innately threatening stimulus by preventing escape responses when activity of vLGN inhibitory neurons is high, whereas a decrease in vLGN activity may be necessary to initiate a response to threat. To test that hypothesis, we manipulated vLGN activity during looming-evoked escape behavior (Figure 3). Control mice reliably escape to the shelter in response to a high-contrast looming stimulus (see Figures 3I and 3L; Video S1), whereas lower-contrast looming spots have lower threat saliency (Evans et al., 2018) and evoke escapes with decreased likelihood (Figures 3C, 3H, and 3I; Video S2). Upon suppression of hM4Di-expressing vLGN GABAergic neurons after systemic CNO injection (Figure 3B), mice became much more likely to escape even to low-contrast looming stimuli (Figure 3C; mean escape probability, saline: 37.3% ± 9.56%, CNO: 81.9% ± 10.4%, p = 0.0107, independent two-sample t test). Moreover, they spent more time in the shelter and showed more spontaneous escapes (Figures 3D and 3E), confirming a heightened anxiety-like state during suppression of vLGN activity. CNO injections in control animals without hM4Di expression had no effect on escape behavior (Figures S6A–S6C).

To test whether a transient decrease in vLGN activity is sufficient to increase escape probability, we next expressed the soma-targeted inhibitory opsin stGtACR2 (Mahn et al., 2018) in vLGN GABAergic neurons (Figure 3F). Indeed, bilateral optogenetic inhibition of vLGN neurons during the threat stimulus significantly increased escape probability to low-contrast looming stimuli (Figures 3G and 3H; median escape probability control: 22.7% [range, 9.09%–28.6%], laser: 52.1% [range, 44.4%–75%], p = 1.95 × 10^{-3}, Wilcoxon signed-rank test; Video S3), thus shifting the psychometric response curve (Figure 3I). Because suppressing GABAergic cells in the vLGN enhanced escape responses, we next tested whether activation of those neurons would have the opposite effect on behavior. When we bilaterally activated vLGN GABAergic neurons during presentation of the looming stimulus using ChR2 (Figures 3J and S6D), the defensive response to the stimulus was completely blocked. Mice never escaped to the shelter (Figures 3K and 3L; median escape probability control: 100% [range, 83.3%–100%], laser: 0% [range, 0%–0%], p = 7.81 × 10^{-3}; Wilcoxon signed-rank test; Video S4), and they did not show other apparent defensive reactions, such as freezing in response to high-contrast looming stimuli (Figures 3M and S6E). Activation of vLGN neurons also fully prevented escape when applied after the onset of the stimulus but before the initiation of an escape (Figure 3N). In contrast, vLGN activation was much less effective in altering the animals' behavior when applied after they had already initiated an escape (Figure 3N). Laser stimulation alone did not affect escape behavior when optogenetic constructs were not expressed in the vLGN, excluding potential light-induced artifacts (Figures S6F and S6G). Together, these results show that the vLGN exerts strong bidirectional control over instinctive escape behavior. As predicted from the signals in vLGN axons (Figure 1), high vLGN activity prevents escape, whereas low activity promotes escape responses.

Activating the vLGN reduces activity in mSC

Neuronal circuits in mSC integrate threat evidence and are crucial for the initiation of escape responses (Evans et al., 2018; Shang et al., 2018; Branco and Redgrave, 2020). Moreover, the vLGN sends prominent projections to mSC that convey threat-related signals (Figure 1). To determine how vLGN activity affects neurons in mSC, we optogenetically activated GABAergic vLGN neurons expressing ChR2 and recorded action potential firing of mSC neurons with Neuropixels probes in awake, head-fixed animals (Jun et al., 2017; Figures 4A and S7A). Activation of

CellPress
OPEN ACCESS

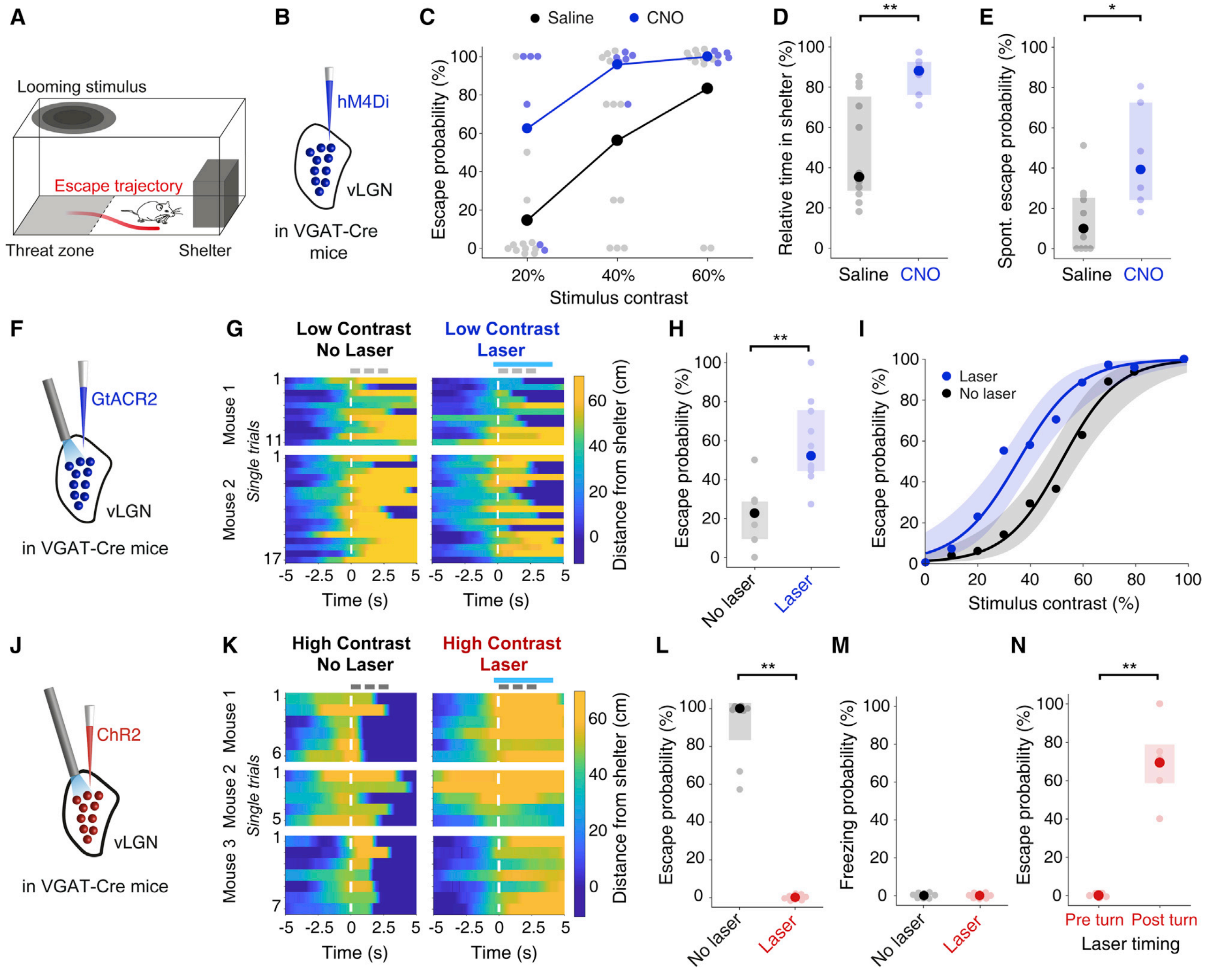

Figure 3. vLGN bidirectionally controls escape from imminent visual threat

(A) Experimental schematic.

(B) Experimental approach: bilateral expression of hM4Di in vLGN of VGAT-Cre mice for inhibition of GABAergic vLGN neurons.

(C) Mean escape probability in response to looming stimuli of different contrasts for systemic CNO- (blue, n = 6 mice) and saline-injected (black, n = 12 mice) animals. Pale dots represent data from single animals here and in all following plots.

(D) Median relative time spent in the shelter after exposure to the first looming stimulus for CNO- (blue, n = 6 mice) and saline-injected (black, n = 12 mice) animals. Error bars represent IQR across mice. $p = 3.23 \times 10^{-3}$, Wilcoxon rank-sum test.

(E) Median spontaneous escape probability after exposure to the first looming stimulus for CNO- (blue, n = 6 mice) or saline-injected (black, n = 12 mice) animals. Error bars represent IQR across mice. $p = 0.0102$, Wilcoxon rank-sum test.

(F) Schematic of the experimental approach: bilateral optogenetic inhibition of GABAergic vLGN neurons during looming stimulus presentation after expression of stGtACR2.

(G) Single trials of low-contrast (30%–40%) looming stimulus presentation in control trials (left, no laser) and with optogenetic inhibition of vLGN (right, laser), showing the mice's distance from the shelter (shelter position, −10 to 0 cm) over time, aligned to stimulus onset (white dashed line) in two example mice. Gray and blue lines on top indicate timing and duration of looming stimuli and laser stimulation, respectively. Trials are in chronological order.

(H) Median escape probability in response to low-contrast looming stimuli in control trials (black) and bilateral vLGN inhibition trials (blue); n = 10 mice. Error bars represent IQR across mice. $p = 1.95 \times 10^{-3}$, Wilcoxon signed-rank test.

(I) Mean escape probability as a function of looming-stimulus contrast in control trials (black) and bilateral vLGN inhibition trials (blue); n = 10 mice. Shading shows 95% confidence interval of the logistic regression of escape probability across mice.

(J) Schematic of the experimental approach: bilateral optogenetic stimulation of GABAergic vLGN neurons during looming-stimulus presentation after expression of ChR2.

(K) Similar to (G), but showing behavior during single trials of high-contrast looming stimulus (99%) presentation in control trials (left) and in trials with optogenetic ChR2 stimulation (right).

(L) Median escape probability in response to high-contrast looming stimuli in control trials (black) and bilateral vLGN stimulation trials (red); n = 8 mice. Error bars represent IQR across mice. $p = 7.81 \times 10^{-3}$, Wilcoxon signed-rank test.

(legend continued on next page)

GABAergic vLGN neurons suppressed responses in the mSC to looming stimuli of all contrasts (Figures 4B–4E). This effect was particularly strong in the intermediate and deep layers of the mSC (Figure 4F), which have been shown to be crucial for mediating looming-evoked escape responses (Evans et al., 2018). Many neurons in these layers responded to visual stimuli, including looming spots or drifting gratings, whereas other neurons either showed responses only to auditory stimuli or were not responsive to any of the sensory stimuli presented (Figures S7B and S7C). Interestingly, the vLGN did not influence those neurons equally, but showed specificity in its effect on the mSC: although vLGN activation suppressed activity of visually responsive neurons (median suppression of spike rate = 25.2%; 52.4% of units significantly suppressed by laser), neurons that did not respond to visual stimuli were, on average, not affected by vLGN stimulation (Figure 4G; median suppression of spike rate = 2.53%; 20.4% of units significantly suppressed by laser). The subset of neurons that specifically responded to visual, but not auditory, stimuli were most strongly suppressed by vLGN stimulation (median suppression of spike rate = 36.1%; 53.2% of units significantly suppressed by laser), whereas neurons responding only to auditory stimuli showed, on average, no effect (Figure 4H; median suppression of spike rate = −5.58%, 14.3% of units significantly suppressed by laser). The inhibitory influence of the vLGN on the intermediate and deep layers of the mSC is, thus, modality specific, suggesting that that pathway may specifically regulate defensive responses to visual, but not auditory threat.

Activating vLGN axons in mSC reduces escape from visual threat

To test whether vLGN neurons projecting to the mSC preferentially control visually evoked escape, we optogenetically activated GABAergic vLGN axons expressing ChR2 within the mSC (Figure 5A). Notably, laser stimulation was performed, with a single fiber placed over the midline between the SC hemispheres, covering only a small part of the SC (Figure S8A). Nevertheless, activating vLGN axons in the mSC strongly reduced the escape probability to looming stimuli (Figures 5A–5C; mean escape probability, intermediate contrast, control: 70.8% ± 6.57%, laser: 29.6% ± 4.23%, $p = 9.60 \times 10^{-4}$, paired t test) and consistently shifted the psychometric response curve, rendering mice less reactive to visual threat stimuli of different contrasts (Figure 5C).

To assess whether the influence of vLGN projections to the mSC on escape behavior is modality specific, we exposed the same mice to high-frequency sounds in the threat zone (Figure 5D), similar to those presented during single-unit recordings in the mSC (Figure 4H). Loud sounds very reliably trigger escape to a shelter in mice, similar to high-contrast looming stimuli (Evans et al., 2018). To achieve high sensitivity in the behavioral readout of vLGN manipulation, we adjusted sound levels to induce escape in roughly 50% of trials. Optogenetic activation of GABAergic vLGN axons in the mSC did not significantly alter the probability of sound-evoked escapes (Figure 5E; mean escape probability, control: 52.4% ± 6.09%, laser: 43.1% ± 7.62%, p = 0.223, paired t test). Therefore, increasing the activity of inhibitory vLGN inputs in the mSC strongly reduces the likelihood of escape from visual, but not auditory, threats, although the maximum running speed during escape from both visual and auditory threats was decreased during laser stimulation (Figure 5F). Laser stimulation in the mSC of control animals that did not express ChR2 in the vLGN had no effect on escape behavior (Figures S8B and S8C). These results indicate that the influence of vLGN GABAergic input on computations in the mSC is modality specific, exerting a stronger effect on visually evoked, rather than sound-evoked threat responses.

DISCUSSION

We identified a novel pathway for control of instinctive defensive behavior. vLGN activity reflects an animal's previous experience of threat, is modulated by the perceived level of danger in the environment, and causally controls both escape responses to imminent visual threats as well as general defensive behavior in open, exposed environments. Calcium photometry and optogenetic manipulations revealed that increased activity in GABAergic vLGN neurons projecting to the mSC prevents escape responses, whereas low activity in vLGN neurons promotes escape and other risk-avoidance behaviors. Activating vLGN projections in the mSC affects reactions to visual threats more strongly than reactions to auditory threats, likely by suppressing activity in visually, but not sound-responsive neurons in the mSC. Thus, the vLGN regulates the threshold for instinctive defensive actions, depending on the modality of imminent threat and the animal's anticipation of danger in the environment. Our results add to a growing body of evidence that prethalamic long-range inhibitory pathways exert a powerful influence on behavior (Chou et al., 2018; Zhao et al., 2019; Wang et al., 2019; Venkataraman et al., 2019; Huang et al., 2019, 2021; Hormigo et al., 2020; Ahmadlou et al., 2021).

Anatomically, the vLGN continues into the intergeniculate leaflet (IGL) on one side and zona incerta (ZI) on the other (see Figure 1A), and spillover of viral vectors into those areas cannot always be prevented. However, their projection pattern in the SC is different to that of the vLGN (Figures S9A and S9B), and expression of genetic constructs in the IGL or ZI cannot explain our results (Figures S9C–S9R; also see Method details: Surgical procedures). Nonetheless, there is no apparent border between the vLGN and the ZI, suggesting that the medial subregion of the vLGN and the very lateral end of the ZI might form a contiguous structure.

The vLGN is thought to be predominantly a visual area because it receives prominent input from both the retina and layer-5 neurons of visual cortical regions (Bourassa and

(M) Median freezing probability in response to high-contrast looming stimuli in no-laser control trials (black) and bilateral-laser stimulation trials (red). N = 8 animals.

(N) Mean escape probability in response to high-contrast looming stimuli (99%) in trials in which vLGN stimulation was initiated after stimulus onset, before the mouse turned toward the shelter (left), and after the mouse turned toward the shelter (right); n = 5 mice. Error bars represent SEM across mice. $p = 2.12 \times 10^{-3}$, dependent t test for paired samples.

CellPress
OPEN ACCESS

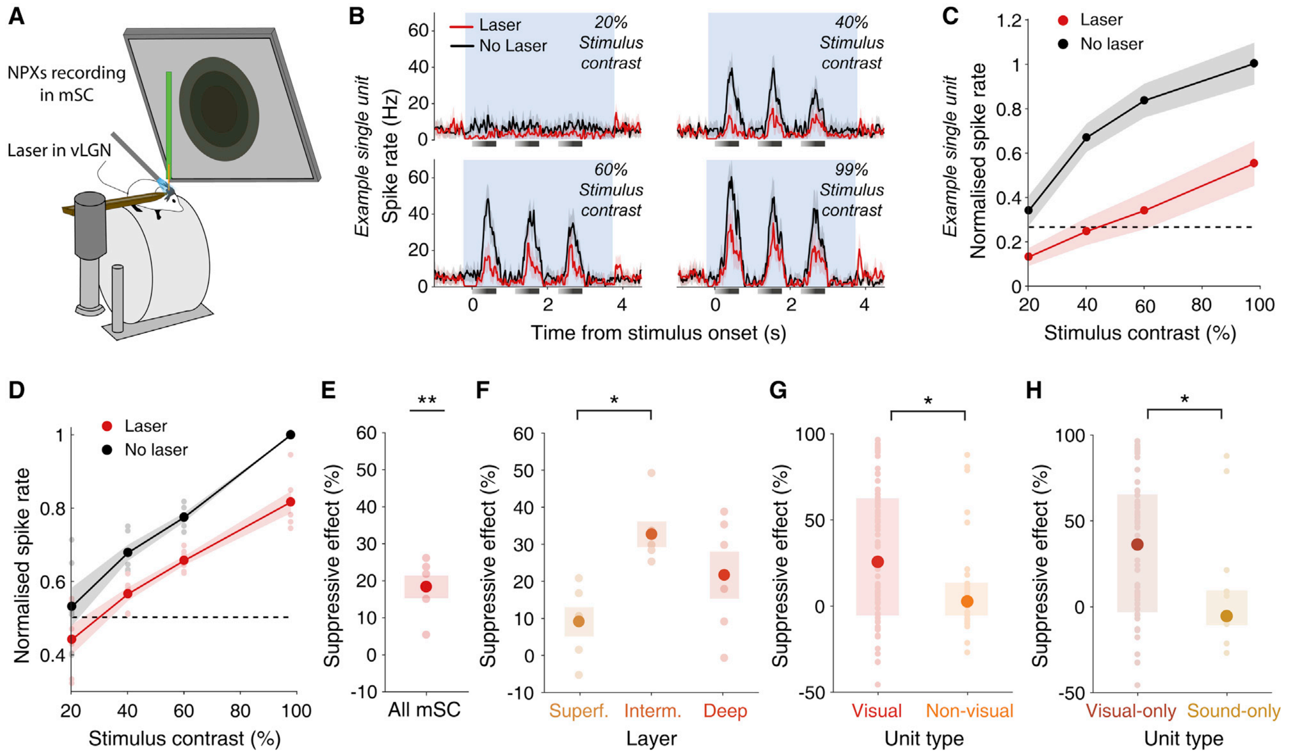

Figure 4. Activating vLGN reduces activity in mSC

(A) Schematic of the experimental setup for Neuropixels (NPXs) recordings in the mSC during optogenetic stimulation of ChR2-expressing GABAergic vLGN neurons in awake, head-fixed mice.

(B) Mean spike rates of an example single unit in the mSC in response to three consecutive looming stimuli of different contrasts (gray bars) during vLGN stimulation (red) and in control trials (black). Background shading shows the laser stimulation period. Shading shows the 95% confidence interval of the mean across trials.

(C) Mean spike rate of the single unit shown in (B) during a 3-s window from stimulus onset in control (black) and laser trials (red), normalized to the mean response to 99%-contrast looming stimuli in control trials. Shading shows the 95% confidence interval of the mean across trials. The dashed line represents the normalized mean pre-stimulus spike rate of the single unit.

(D) Mean population spike rate of all recorded units in the mSC during looming stimuli of different contrasts in control (black) and laser trials (red), normalized to the response to 99%-contrast looming stimuli; n = 6 mice. Pale dots represent data from single animals. Shading shows SEM across mice. Dashed line represents the normalized mean pre-stimulus activity.

(E) Mean suppressive effect of vLGN stimulation on the population spike rate of all recorded units in the mSC during a 99%-contrast looming stimulus, n = 6 mice. Error bars represent SEM across mice. Pale dots represent data from single animals; $p = 1.70 \times 10^{-3}$, one-sample t test.

(F) Mean suppressive effect of vLGN stimulation on the population-spike rate of recorded units in superficial (Superf., yellow), intermediate (Interm., orange), or deep (Deep, red) layers in the mSC during 99%-contrast looming stimulus presentation. Superficial-intermediate, p = 0.0126; intermediate-deep, p = 0.257; superficial-deep, p = 0.191. Tukey's honest significance test preceded by repeated-measures, one-way analysis of variance, p = 0.0161).

(G) Median suppressive effect of vLGN stimulation during spontaneous activity on single units responding to either looming or grating stimuli (visual, red, n = 84 units), and on single units not responding to any visual stimulus (non-visual, orange, n = 34 units) in intermediate and deep layers of the mSC. Pale dots represent data from single units. Error bars represent IQR across single units. Single units from 11 recordings in six animals. p = 0.0121, Wilcoxon rank-sum test.

(H) Median suppressive effect of vLGN stimulation during spontaneous activity on single units responding to either looming or grating stimuli, but not to sounds (visual-only, dark red, n = 62 units) and on single units not responding to any visual stimulus, but showing a significant response to sounds (sound-only, dark orange, n = 14 single units) in intermediate and deep layers of the mSC. Single units from 11 recordings in six animals. p = 0.0240, Wilcoxon rank-sum test.

Deschênes, 1995; Harrington, 1997; Monavarfeshani et al., 2017). A significant fraction of vLGN neurons has been reported to respond to visual stimuli and exhibits visual receptive fields (Spear et al., 1977; Sumitomo et al., 1979; Pienaar et al., 2018; Ciftcioglu et al., 2020). Consistent with those previous studies, we observed higher average vLGN activity with increasing luminance and lower activity with decreasing luminance (Figure S3C). Interestingly, although vLGN axons in the mSC showed the same trend, their average visual responses were much weaker (Figure S3C). Instead, the activity of vLGN inputs to the mSC reflected the animal's past encounters of threatening stimuli: vLGN axon activity during exploration of the arena decreased after mice were exposed to a threatening stimulus and was lowest in the area in which the threat was encountered. In contrast, vLGN axon activity was high in naive mice and when mice were habituated and no

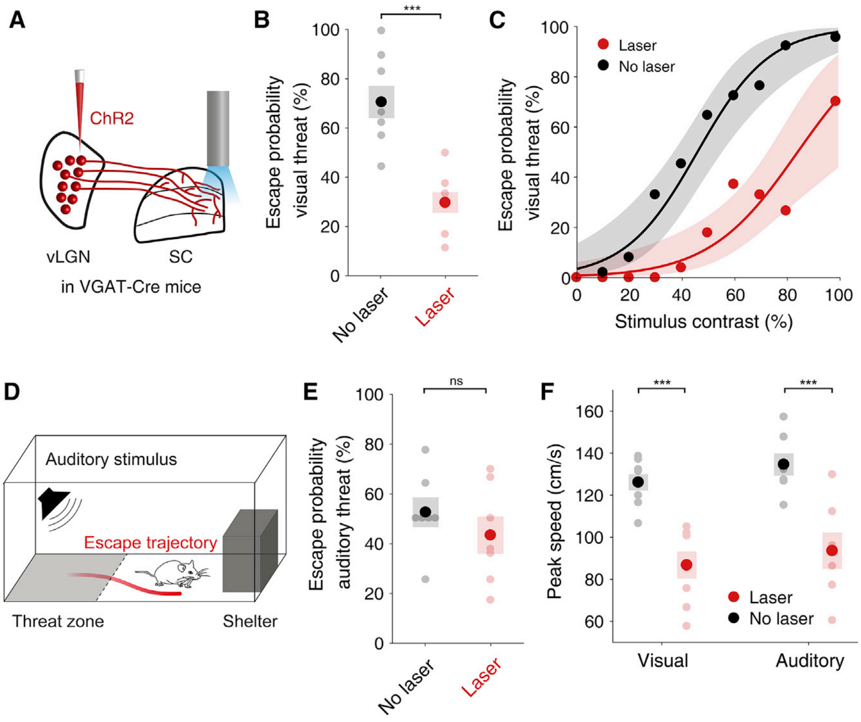

Figure 5. Activating vLGN axons in the mSC suppresses escape from visual threat

(A) Schematic of the experimental approach: optogenetic stimulation of ChR2-expressing GABAergic vLGN axons in the mSC during looming stimulus presentation.

(B) Mean escape probability in response to intermediate-contrast (50%–60%) looming stimuli in control trials (black) and trials with laser stimulation of vLGN axons in the mSC (red); n = 8 mice. Error bars represent SEM across mice. $p = 9.60 \times 10^{-4}$, dependent t test for paired samples.

(C) Mean escape probability as a function of looming stimulus contrast in control trials (black) and trials with laser stimulation of vLGN axons in the mSC (red); n = 8 mice. Error bars represent the 95% confidence interval of the logistic regression of escape probability across mice.

(D) Schematic of the experimental approach. Mice were presented with high-frequency sounds in the threat zone instead of looming stimuli.

(E) Mean escape probability in response to high-frequency sounds in control trials (black) and in trials with laser stimulation of vLGN axons in mSC (red); n = 7 mice. Error bars represent SEM across mice. p = 0.223, dependent t test for paired samples.

(F) Mean peak running speed during escape in control escape trials (black) and escape trials during stimulation of vLGN axons in mSC (red) in response to looming stimuli (left, n = 8 mice, $p = 4.28 \times 10^{-6}$, dependent t test for paired samples) and in response to high-frequency sounds (right, n = 7 mice, $p = 6.88 \times 10^{-4}$). In all plots, pale dots represent data from single animals.

longer escaped from looming stimuli, presumably because they had learned that these had no negative consequences. Moreover, activity was very high in the animal's adopted safe place, the shelter.

These signal characteristics are consistent with the notion that vLGN inputs to the mSC represent the animal's anticipation of threat and its assessment of risk at different locations in its environment. Although the vLGN does not receive direct input from the amygdala, traditionally associated with processing of acquired fear and anxiety (LeDoux, 2003; Tovote et al., 2015), it does receive projections from the ventromedial hypothalamus and the dorsal premammillary nucleus, linked to defensive behaviors and the implementation of defensive emotional states (Kunwar et al., 2015; Silva et al., 2016a, 2016b; Kennedy et al., 2020; Wang et al., 2021). Moreover, vLGN signals are likely to be influenced by prominent input from visual and other cortical areas (Bourassa and Deschênes, 1995; Harrington, 1997; Monavarfeshani et al., 2017), perhaps allowing representations of learned visual and spatial context to shape instinctive behavioral responses.

Irrespective of how the vLGN derives these signals, our results indicate that the effect of an animal's anticipation of threat on its behavior is, at least partly, mediated by vLGN circuits. Activity of vLGN GABAergic neurons causally affects animals' defensive behaviors in open, exposed spaces that they perceive as aversive: when vLGN activity is low—as is the case when anticipation of threat is high—animals show increased risk avoidance and less exploratory behavior. The vLGN also bidirectionally controls the reaction to imminent threats in such environments: low vLGN activity increases the probability of the mouse to escape to the shelter, whereas high activity in vLGN GABAergic neurons completely abolishes escape responses to threatening visual stimuli. Our results, therefore, suggest that a release from vLGN inhibition may be necessary to allow escape initiation and that vLGN circuits control the threshold for triggering defensive responses to imminent visual threats. Moreover, because vLGN activity increases as animals learn that looming stimuli do not pose danger, neural pathways through the vLGN likely contribute to mediating the behavioral habituation to threat stimuli.

The vLGN strongly projects to the mSC, which has been identified as a crucial hub for threat-evoked, defensive behaviors (Wei et al., 2015; Shang et al., 2019; Branco and Redgrave, 2020). Visual threat-evoked neural activity in deeper layers of the mSC has been shown to be necessary and sufficient for initiation of escape through connections to the dorsal PAG (Evans et al., 2018). We find that activation of vLGN GABAergic neurons prominently inhibits activity in intermediate and deep mSC layers. Moreover, experience of threat only modulates the activity in vLGN axons to the mSC, not the average activity within the vLGN itself. Although calcium signals of vLGN axons in the mSC could be modulated locally via pre-synaptic mechanisms (Alford and Schwartz, 2009), it is likely that the activity of vLGN neurons projecting to the mSC differs from that of the average vLGN population, and

CellPress
OPEN ACCESS

that this subgroup of vLGN neurons contributes to the vLGN's threat-experience-dependent effect on escape behavior: elevated activity in vLGN GABAergic neurons suppresses responses in the mSC, thereby preventing escape initiation, whereas low vLGN activity could increase escape probability through disinhibition of mSC circuits (Evans et al., 2018). The rapid increase of activity in vLGN axons in the mSC after escape onset may close the window for initiation of further actions that interfere with escape.

Activating vLGN axons in the mSC had a stronger influence on visually evoked, than on sound-evoked escape responses. Therefore, although the vLGN integrates different visual and non-visual signals, vLGN neurons projecting to the mSC appear to have a modality-specific effect on defensive reactions. We uncovered a likely circuit mechanism for this specificity in behavioral influence: activating vLGN most strongly suppressed intermediate and deep-layer mSC neurons that exclusively responded to visual stimuli, whereas sound-responsive mSC neurons were, on average, not influenced by vLGN manipulation. These results suggest that vLGN's effect on mSC circuits mediates the modality-specific influence of the vLGN on escape behavior. By affecting predominantly visually responsive neurons in the mSC motor-related layers, the vLGN can specifically suppress behavioral responses to visual stimuli. Interestingly, our findings indicate that the mSC contains modality-specific circuits for sensory-motor transformations in response to threat signals that can be differentially regulated. Pathways in the mSC for different modalities likely converge in the PAG, which has been shown to encode motor aspects of the escape and to control escape vigor (Evans et al., 2018). The optic fiber for optogenetic stimulation was positioned deep enough for the laser light to potentially reach vLGN axons in the PAG, which may explain the modality-independent effect of optogenetic manipulation on escape vigor.

In addition, vLGN projections target several other areas that have been linked to defensive behavior, such as the lateral posterior nucleus and the midline areas in the thalamus (Wei et al., 2015; Salay et al., 2018; Shang et al., 2018), the habenula (Huang et al., 2019), and the pontine reticular nucleus (Yeomans and Frankland, 1995). These pathways likely also contribute to the vLGN's effect on defensive actions, for instance the more general influence of the vLGN on risk-avoidance behavior that we observed in the open field and elevated plus maze. Given that the vLGN contains multiple inhibitory cell classes (Sabbagh et al., 2020), it is intriguing to speculate that those classes form specific pathways for regulating different processes that define defensive behavior, including the processing of visual threat, defensive-action selection and execution, and setting the anxiety-related internal state or the motivational drive.

In summary, we find that vLGN is a key node in a distributed network of brain areas that may contribute to different aspects of defensive behavior in mammals (LeDoux, 2003; Biagioni et al., 2013; Kunwar et al., 2015; Wei et al., 2015; Tovote et al., 2016; Silva et al., 2016a; Lecca et al., 2017; Salay et al., 2018; Li et al., 2018; Chou et al., 2018; Shang et al., 2018; Wang et al., 2019, 2021; Zhou et al., 2019; Branco and Redgrave, 2020; Hormigo et al., 2020). Given its extensive connectivity with cortical and subcortical structures, we suggest that the vLGN integrates visual information with non-sensory signals that track an animal's risk assessment, based on its knowledge of the environment. In turn, that allows flexible control of instinctive responses to visual threats and general defensive behavior in open environments in which threat may be encountered, via potent inhibitory projections that regulate the threshold for escape and other risk-avoiding actions. More generally, inhibitory hubs of the prethalamus, such as the vLGN or the ZI, are well poised to link activity among circuits for fast, instinctive responses in the midbrain and those for more deliberate processing in the forebrain to allow adaptive control of reactive behaviors, depending on the animal's experience or assessment of the environment.

STAR★METHODS

Detailed methods are provided in the online version of this paper and include the following:

- KEY RESOURCES TABLE
- RESOURCE AVAILABILITY
  - Lead contact
  - Materials availability
  - Data and code availability
- EXPERIMENTAL MODEL AND SUBJECT DETAILS
- METHOD DETAILS
  - Viruses
  - Surgical procedures
  - Histology
  - Electrophysiological slice recordings
  - Experimental set-up of behavioral procedures
  - Behavioral protocols
  - Visual stimuli
  - Auditory stimuli
  - Optogenetic manipulation
  - Fiber photometry experiments
  - Extracellular electrophysiological recordings
- QUANTIFICATION AND STATISTICAL ANALYSIS
  - Electrophysiological slice recordings
  - Pre-processing of behavioral data
  - Behavior extraction
  - Behavior analysis
  - Calcium imaging
  - Extracellular electrophysiological recordings
  - Statistics

SUPPLEMENTAL INFORMATION

Supplemental information can be found online at https://doi.org/10.1016/j.neuron.2021.09.003.

ACKNOWLEDGMENTS

We thank Tom Mrsic-Flogel, Petr Znamenskiy, Antonin Blot, Sara Mederos, and Philip Shamash for helpful discussions and comments on the manuscript. We thank Fred Marbach for help with fiber photometry, Dario Campagner and Dominic Evans for help with building the behavioral setup, Ioana Gasler for help with surgeries, Rob Campbell for help with serial two-photon imaging, Thomas Akam for providing the pyPhotometry system, Kostas Betsios for

providing Mantis, Andrei Khilkevich for help with extracellular Neuropixels recordings, and Gemma Estrada Girona from the Sainsbury Wellcome Centre viral core for providing viruses. This work was supported by the Sainsbury Wellcome Centre core grant from the Gatsby Charitable Foundation and the Wellcome Foundation (090843/F/09/Z), a Wellcome investigator award (S.B.H., 219561/Z/19/Z), and a Sir Henry Wellcome postdoctoral fellowship (A.M.-S., 213692/Z/18/Z).

AUTHOR CONTRIBUTIONS

A.F. and S.B.H. conceived the study and designed the experiments, with help from T.B., A.V.S., and A.M.-S. A.F. built the experimental setups, performed the experiments, and analyzed the data, with help from A.M.K., N.V., and A.V.S. A.M.-S. performed the immunostaining and analyzed histology data. Y.L.T. performed *in vitro* recordings. A.F. and S.B.H. wrote the paper, with inputs from all authors.

DECLARATION OF INTERESTS

The authors declare no competing interests.

Received: April 21, 2020
Revised: May 22, 2021
Accepted: September 1, 2021
Published: October 5, 2021

REFERENCES

Ahmadlou, M., Houba, J.H.W., van Vierbergen, J.F.M., Giannouli, M., Gimenez, G.-A., van Weeghel, C., Darbanfouladi, M., Shirazi, M.Y., Dziubek, J., Kacem, M., de Winter, F., and Heimel, J.A. (2021). A cell type–specific cortico-subcortical brain circuit for investigatory and novelty-seeking behavior. Science *372*, eabe9681. https://doi.org/10.1126/science.abe9681.

Akam, T., and Walton, M.E. (2019). pyPhotometry: open source Python based hardware and software for fiber photometry data acquisition. Sci. Rep. *9*, 3521.

Alford, S., and Schwartz, E. (2009). Presynaptic inhibition. In Encyclopedia of Neuroscience, L.R. Squire, ed. (Academic Press), pp. 1001–1006.

Armbruster, B.N., Li, X., Pausch, M.H., Herlitze, S., and Roth, B.L. (2007). Evolving the lock to fit the key to create a family of G protein-coupled receptors potently activated by an inert ligand. PNAS *104*, 5163–5168.

Biagioni, A.F., de Freitas, R.L., da Silva, J.A., de Oliveira, R.C., de Oliveira, R., Alves, V.M., and Coimbra, N.C. (2013). Serotonergic neural links from the dorsal raphe nucleus modulate defensive behaviours organised by the dorsomedial hypothalamus and the elaboration of fear-induced antinociception via locus coeruleus pathways. Neuropharmacology *67*, 379–394.

Blanchard, R.J., Blanchard, D.C., Rodgers, J., and Weiss, S.M. (1990). The characterization and modelling of antipredator defensive behavior. Neurosci. Biobehav. Rev. *14*, 463–472.

Bourassa, J., and Deschênes, M. (1995). Corticothalamic projections from the primary visual cortex in rats: a single fiber study using biocytin as an anterograde tracer. Neuroscience *66*, 253–263.

Brainard, D.H. (1997). The psychophysics toolbox. Spat. Vis. *10*, 433–436.

Branco, T., and Redgrave, P. (2020). The neural basis of escape behavior in vertebrates. Annu. Rev. Neurosci. *43*, 417–439.

Chen, T.-W., Wardill, T.J., Sun, Y., Pulver, S.R., Renninger, S.L., Baohan, A., Schreiter, E.R., Kerr, R.A., Orger, M.B., Jayaraman, V., et al. (2013). Ultrasensitive fluorescent proteins for imaging neuronal activity. Nature *499*, 295–300.

Chou, X.L., Wang, X., Zhang, Z.G., Shen, L., Zingg, B., Huang, J., Zhong, W., Mesik, L., Zhang, L.I., and Tao, H.W. (2018). Inhibitory gain modulation of defense behaviors by zona incerta. Nat. Commun. *9*, 1151.

Ciftcioglu, U.M., Suresh, V., Ding, K.R., Sommer, F.T., and Hirsch, J.A. (2020). Visual information processing in the ventral division of the mouse lateral geniculate nucleus of the thalamus. J. Neurosci. *40*, 5019–5032.

Crawley, J.N. (1985). Exploratory behavior models of anxiety in mice. Neurosci. Biobehav. Rev. *9*, 37–44.

Cui, G., Jun, S.B., Jin, X., Pham, M.D., Vogel, S.S., Lovinger, D.M., and Costa, R.M. (2013). Concurrent activation of striatal direct and indirect pathways during action initiation. Nature *494*, 238–242.

De Franceschi, G., Vivattanasarn, T., Saleem, A.B., and Solomon, S.G. (2016). Vision guides selection of freeze or flight defense strategies in mice. Curr. Biol. *26*, 2150–2154.

Dunn, T.W., Gebhardt, C., Naumann, E.A., Riegler, C., Ahrens, M.B., Engert, F., and Del Bene, F. (2016). Neural circuits underlying visually evoked escapes in larval zebrafish. Neuron *89*, 613–628.

Evans, D.A., Stempel, A.V., Vale, R., Ruehle, S., Lefler, Y., and Branco, T. (2018). A synaptic threshold mechanism for computing escape decisions. Nature *558*, 590–594.

Evans, D.A., Stempel, A.V., Vale, R., and Branco, T. (2019). Cognitive control of escape behaviour. Trends Cogn. Sci. *23*, 334–348.

Fotowat, H., and Gabbiani, F. (2011). Collision detection as a model for sensory-motor integration. Annu. Rev. Neurosci. *34*, 1–19.

Harrington, M.E. (1997). The ventral lateral geniculate nucleus and the intergeniculate leaflet: interrelated structures in the visual and circadian systems. Neurosci. Biobehav. Rev. *21*, 705–727.

Hormigo, S., Zhou, J., and Castro-Alamancos, M.A. (2020). Zona incerta GABAergic output controls a signaled locomotor action in the midbrain tegmentum. eNeuro *7*, ENEURO.0390-192020.

Huang, L., Xi, Y., Peng, Y., Yang, Y., Huang, X., Fu, Y., Tao, Q., Xiao, J., Yuan, T., An, K., et al. (2019). A Visual circuit related to habenula underlies the antidepressive effects of light therapy. Neuron *102*, 128–142.e8.

Huang, X., Huang, P., Huang, L., Hu, Z., Liu, X., Shen, J., Xi, Y., Yang, Y., Fu, Y., Tao, Q., et al. (2021). A Visual circuit related to the nucleus reuniens for the spatial-memory-promoting effects of light treatment. Neuron *109*, 347–362.e7.

Jun, J.J., Steinmetz, N.A., Siegle, J.H., Denman, D.J., Bauza, M., Barbarits, B., Lee, A.K., Anastassiou, C.A., Andrei, A., Aydın, Ç., et al. (2017). Fully integrated silicon probes for high-density recording of neural activity. Nature *551*, 232–236.

Kennedy, A., Kunwar, P.S., Li, L.Y., Stagkourakis, S., Wagenaar, D.A., and Anderson, D.J. (2020). Stimulus-specific hypothalamic encoding of a persistent defensive state. Nature *586*, 730–734.

Kleiner, M., Brainard, D., Pelli, D., Ingling, A., Murray, R., and Broussard, C. (2007). What's new in psychtoolbox-3. Perception *36*, 1–16.

Krashes, M.J., Koda, S., Ye, C., Rogan, S.C., Adams, A.C., Cusher, D.S., Maratos-Flier, E., Roth, B.L., and Lowell, B.B. (2011). Rapid, reversible activation of AgRP neurons drives feeding behavior in mice. J. Clin. Invest. *121*, 1424–1428.

Kunwar, P.S., Zelikowsky, M., Remedios, R., Cai, H., Yilmaz, M., Meister, M., and Anderson, D.J. (2015). Ventromedial hypothalamic neurons control a defensive emotion state. eLife *4*, e06633.

Langel, J., Ikeno, T., Yan, L., Nunez, A.A., and Smale, L. (2018). Distributions of GABAergic and glutamatergic neurons in the brains of a diurnal and nocturnal rodent. Brain Res. *1700*, 152–159.

Lecca, S., Meye, F.J., Trusel, M., Tchenio, A., Harris, J., Schwarz, M.K., Burdakov, D., Georges, F., and Mameli, M. (2017). Aversive stimuli drive hypothalamus-to-habenula excitation to promote escape behavior. eLife *6*, e30697.

LeDoux, J. (2003). The emotional brain, fear, and the amygdala. Cell. Mol. Neurobiol. *23*, 727–738.

Lefler, Y., Campagner, D., and Branco, T. (2020). The role of the periaqueductal gray in escape behavior. Curr. Opin. Neurobiol. *60*, 115–121.

CellPress
OPEN ACCESS

Lerner, T.N., Shilyansky, C., Davidson, T.J., Evans, K.E., Beier, K.T., Zalocusky, K.A., Crow, A.K., Malenka, R.C., Luo, L., Tomer, R., and Deisseroth, K. (2015). Intact-brain analyses reveal distinct information carried by SNc dopamine subcircuits. Cell *162*, 635–647.

Li, L., Feng, X., Zhou, Z., Zhang, H., Shi, Q., Lei, Z., Shen, P., Yang, Q., Zhao, B., Chen, S., et al. (2018). Stress accelerates defensive responses to looming in mice and involves a locus coeruleus-superior colliculus projection. Curr. Biol. *28*, 859–871.e5.

Livingston, C.A., and Fedder, S.R. (2003). Visual-ocular motor activity in the macaque pregeniculate complex. J. Neurophysiol. *90*, 226–244.

Madisen, L., Zwingman, T.A., Sunkin, S.M., Oh, S.W., Zariwala, H.A., Gu, H., Ng, L.L., Palmiter, R.D., Hawrylycz, M.J., Jones, A.R., et al. (2010). A robust and high-throughput Cre reporting and characterization system for the whole mouse brain. Nat. Neurosci. *13*, 133–140.

Magnin, M., Jeannerod, M., and Putkonen, P. (1974). Vestibular and saccadic influences on dorsal and ventral nuclei of the lateral geniculate body. Exp. Brain Res. *21*, 1–18.

Mahn, M., Gibor, L., Patil, P., Cohen-Kashi Malina, K., Oring, S., Printz, Y., Levy, R., Lampl, I., and Yizhar, O. (2018). High-efficiency optogenetic silencing with soma-targeted anion-conducting channelrhodopsins. Nat. Commun. *9*, 4125.

Mathis, A., Mamidanna, P., Cury, K.M., Abe, T., Murthy, V.N., Mathis, M.W., and Bethge, M. (2018). DeepLabCut: markerless pose estimation of user-defined body parts with deep learning. Nat. Neurosci. *21*, 1281–1289.

Mayerich, D., Abbott, L., and McCormick, B. (2008). Knife-edge scanning microscopy for imaging and reconstruction of three-dimensional anatomical structures of the mouse brain. J. Microsc. *231*, 134–143.

Mink, J.W. (1996). The basal ganglia: focused selection and inhibition of competing motor programs. Prog. Neurobiol. *50*, 381–425.

Monavarfeshani, A., Sabbagh, U., and Fox, M.A. (2017). Not a one-trick pony: diverse connectivity and functions of the rodent lateral geniculate complex. Vis. Neurosci. *34*, E012.

Nakagawa, Y. (2019). Development of the thalamus: from early patterning to regulation of cortical functions. Wiley Interdiscip. Rev. Dev. Biol. *8*, e345.

Nelson, A.B., and Kreitzer, A.C. (2014). Reassessing models of basal ganglia function and dysfunction. Annu. Rev. Neurosci. *37*, 117–135.

Oh, S.W., Harris, J.A., Ng, L., Winslow, B., Cain, N., Mihalas, S., Wang, Q., Lau, C., Kuan, L., Henry, A.M., et al. (2014). A mesoscale connectome of the mouse brain. Nature *508*, 207–214.

Pachitariu, M., Steinmetz, N., Kadir, S., Carandini, M., and Harris, K.D. (2016). Kilosort: realtime spike-sorting for extracellular electrophysiology with hundreds of channels. BioRxiv. https://doi.org/10.1101/061481.

Peek, M.Y., and Card, G.M. (2016). Comparative approaches to escape. Curr. Opin. Neurobiol. *41*, 167–173.

Pelli, D.G. (1997). The VideoToolbox software for visual psychophysics: transforming numbers into movies. Spat. Vis. *10*, 437–442.

Pellow, S., Chopin, P., File, S.E., and Briley, M. (1985). Validation of open:-closed arm entries in an elevated plus-maze as a measure of anxiety in the rat. J. Neurosci. Methods *14*, 149–167.

Petreanu, L., Huber, D., Sobczyk, A., and Svoboda, K. (2007). Channelrhodopsin-2-assisted circuit mapping of long-range callosal projections. Nat. Neurosci. *10*, 663–668.

Pienaar, A., Walmsley, L., Hayter, E., Howarth, M., and Brown, T.M. (2018). Commissural communication allows mouse intergeniculate leaflet and ventral lateral geniculate neurons to encode interocular differences in irradiance. J. Physiol. *596*, 5461–5481.

Pologruto, T.A., Sabatini, B.L., and Svoboda, K. (2003). ScanImage: flexible software for operating laser scanning microscopes. Biomed. Eng. Online *2*, 13.

Ragan, T., Kadiri, L.R., Venkataraju, K.U., Bahlmann, K., Sutin, J., Taranda, J., Arganda-Carreras, I., Kim, Y., Seung, H.S., and Osten, P. (2012). Serial two-photon tomography for automated *ex vivo* mouse brain imaging. Nat. Methods *9*, 255–258.

Rossant, C., Kadir, S.N., Goodman, D.F.M., Schulman, J., Hunter, M.L.D., Saleem, A.B., Grosmark, A., Belluscio, M., Denfield, G.H., Ecker, A.S., et al. (2016). Spike sorting for large, dense electrode arrays. Nat. Neurosci. *19*, 634–641.

Roth, B.L. (2016). DREADDs for neuroscientists. Neuron *89*, 683–694.

Sabbagh, U., Govindaiah, G., Somaiya, R.D., Ha, R.V., Wei, J.C., Guido, W., and Fox, M.A. (2020). Diverse GABAergic neurons organize into subtype-specific sublaminae in the ventral lateral geniculate nucleus. J. Neurochem. Published online June 4, 2020. https://doi.org/10.1111/jnc.15101.

Salay, L.D., Ishiko, N., and Huberman, A.D. (2018). A midline thalamic circuit determines reactions to visual threat. Nature *557*, 183–189.

Schiff, W., Caviness, J.A., and Gibson, J.J. (1962). Persistent fear responses in rhesus monkeys to the optical stimulus of "looming". Science *136*, 982–983.

Shang, C., Chen, Z., Liu, A., Li, Y., Zhang, J., Qu, B., Yan, F., Zhang, Y., Liu, W., Liu, Z., et al. (2018). Divergent midbrain circuits orchestrate escape and freezing responses to looming stimuli in mice. Nat. Commun. *9*, 1232.

Shang, C., Liu, A., Li, D., Xie, Z., Chen, Z., Huang, M., Li, Y., Wang, Y., Shen, W.L., and Cao, P. (2019). A subcortical excitatory circuit for sensory-triggered predatory hunting in mice. Nat. Neurosci. *22*, 909–920.

Silva, B.A., Gross, C.T., and Gräff, J. (2016a). The neural circuits of innate fear: detection, integration, action, and memorization. Learn. Mem. *23*, 544–555.

Silva, B.A., Mattucci, C., Krzywkowski, P., Cuozzo, R., Carbonari, L., and Gross, C.T. (2016b). The ventromedial hypothalamus mediates predator fear memory. Eur. J. Neurosci. *43*, 1431–1439.

Spear, P.D., Smith, D.C., and Williams, L.L. (1977). Visual receptive-field properties of single neurons in cat's ventral lateral geniculate nucleus. J. Neurophysiol. *40*, 390–409.

Sumitomo, I., Sugitani, M., Fukuda, Y., and Iwama, K. (1979). Properties of cells responding to visual stimuli in the rat ventral lateral geniculate nucleus. Exp. Neurol. *66*, 721–736.

Sun, L., Tang, Y., Yan, K., Yu, J., Zou, Y., Xu, W., Xiao, K., Zhang, Z., Li, W., Wu, B., et al. (2019). Differences in neurotropism and neurotoxicity among retrograde viral tracers. Mol. Neurodegener. *14*, 8.

Temizer, I., Donovan, J.C., Baier, H., and Semmelhack, J.L. (2015). A visual pathway for looming-evoked escape in larval zebrafish. Curr. Biol. *25*, 1823–1834.

Tervo, D.G.R., Hwang, B.-Y., Viswanathan, S., Gaj, T., Lavzin, M., Ritola, K.D., Lindo, S., Michael, S., Kuleshova, E., Ojala, D., et al. (2016). A designer AAV variant permits efficient retrograde access to projection neurons. Neuron *92*, 372–382.

Tovote, P., Fadok, J.P., and Lüthi, A. (2015). Neuronal circuits for fear and anxiety. Nat. Rev. Neurosci. *16*, 317–331.

Tovote, P., Esposito, M.S., Botta, P., Chaudun, F., Fadok, J.P., Markovic, M., Wolff, S.B.E., Ramakrishnan, C., Fenno, L., Deisseroth, K., et al. (2016). Midbrain circuits for defensive behaviour. Nature *534*, 206–212.

Venkataraman, A., Brody, N., Reddi, P., Guo, J., Gordon Rainnie, D., and Dias, B.G. (2019). Modulation of fear generalization by the zona incerta. Proc. Natl. Acad. Sci. USA *116*, 9072–9077.

Vong, L., Ye, C., Yang, Z., Choi, B., Chua, S., Jr., and Lowell, B.B. (2011). Leptin action on GABAergic neurons prevents obesity and reduces inhibitory tone to POMC neurons. Neuron *71*, 142–154.

Wang, X., Chou, X., Peng, B., Shen, L., Huang, J.J., Zhang, L.I., and Tao, H.W. (2019). A cross-modality enhancement of defensive flight via parvalbumin neurons in zona incerta. eLife *8*, e42728.

Wang, W., Schuette, P.J., Nagai, J., Tobias, B.C., Cuccovia V Reis, F.M., Ji, S., de Lima, M.A.X., La-Vu, M.Q., Maesta-Pereira, S., Chakerian, M., et al. (2021). Coordination of escape and spatial navigation circuits orchestrates versatile flight from threats. Neuron *109*, 1848–1860.e8.

Wei, P., Liu, N., Zhang, Z., Liu, X., Tang, Y., He, X., Wu, B., Zhou, Z., Liu, Y., Li, J., et al. (2015). Processing of visually evoked innate fear by a non-canonical thalamic pathway. Nat. Commun. *6*, 6756.

Yeomans, J.S., and Frankland, P.W. (1995). The acoustic startle reflex: neurons and connections. Brain Res. Brain Res. Rev. *21*, 301–314.

Yilmaz, M., and Meister, M. (2013). Rapid innate defensive responses of mice to looming visual stimuli. Curr. Biol. *23*, 2011–2015.

Zhao, Z.D., Chen, Z., Xiang, X., Hu, M., Xie, H., Jia, X., Cai, F., Cui, Y., Chen, Z., Qian, L., et al. (2019). Zona incerta GABAergic neurons integrate prey-related sensory signals and induce an appetitive drive to promote hunting. Nat. Neurosci. *22*, 921–932.

Zhou, Z., Liu, X., Chen, S., Zhang, Z., Liu, Y., Montardy, Q., Tang, Y., Wei, P., Liu, N., Li, L., et al. (2019). A VTA GABAergic neural circuit mediates visually evoked innate defensive responses. Neuron *103*, 473–488.e6.

CellPress
OPEN ACCESS

STAR★METHODS

KEY RESOURCES TABLE

REAGENT or RESOURCE	SOURCE	IDENTIFIER
**Antibodies**		
Anti-NeuN Antibody, clone A60	Sigma-Aldrich	RRID: AB_2298772
Donkey anti-Mouse IgG Secondary Antibody	Thermo-Fisher	RRID: AB_2762830
**Bacterial and virus strains**		
AAV1-EF1a-doublefloxed-hChR2(H134R)-EYFP-WPRE-HGHpA	unpublished	RRID: Addgene_20298
AAV1-hSyn1-SIO-stGtACR2-FusionRed	Mahn et al., 2018	RRID: Addgene_105677
AAV1-Syn-Flex-GCaMP6f-WPRE-SV40	Chen et al., 2013	RRID: Addgene_100833
AAV1-hSyn-DIO-hM4D(Gi)-mCherry	Krashes et al., 2011	RRID: Addgene_44362
AAV1-EF1a-FLEX-EGFP	SWC Viral Vector Core	N/A
AAV1-EF1a-FLEX-mCherry	SWC Viral Vector Core	N/A
AAVretro-hSyn-Cre	SWC Viral Vector Core	N/A
**Chemicals, peptides, and recombinant proteins**		
DiI	Thermo-Fisher	Cat# D3911
CNO	Sigma Aldrich	Cat# SML2304
Picrotoxin	Bio-Techne	Cat# 1128
Kynurenic acid	Sigma Aldrich	Cat# K3375
**Experimental models: Organisms/strains**		
Slc32a1tm2(cre)Lowl	The Jackson Laboratory	RRID: IMSR_JAX:028862
C57BL/6J	Charles River	RRID: IMSR_CRL:27
Ai14D	The Jackson Laboratory	RRID: IMSR_JAX:007914
**Software and algorithms**		
MATLAB R2017a	The MathWorks Inc.	https://uk.mathworks.com/products/matlab.html; RRID: SCR_001622
Psychophysics Toolbox Version 3	Brainard, 1997; Pelli, 1997; Kleiner et al., 2007	http://psychtoolbox.org; RRID: SCR_002881
ScanImage	Pologruto et al., 2003	Vidrio Technologies, LLC; RRID: SCR_014307
Kilosort2	Pachitariu et al., 2016	https://github.com/MouseLand/Kilosort; RRID: SCR_016422
Phy	Rossant et al., 2016	https://github.com/cortex-lab/phy
DeepLabCut	Mathis et al., 2018	https://github.com/DeepLabCut/DeepLabCut
SpikeGLX	Janelia Research Campus	https://github.com/billkarsh/SpikeGLX
PyPhotometry	Akam and Walton, 2019	https://pyphotometry.readthedocs.io/en/latest/
Mantis	TANDM Solutions Limited	https://www.mantis64.com/

RESOURCE AVAILABILITY

Lead contact

Further information and requests for resources and reagents should be directed to and will be fulfilled by the lead contact, Sonja B. Hofer (s.hofer@ucl.ac.uk).

Materials availability

This study did not generate new unique reagents.

Data and code availability

The datasets supporting the current study have not been deposited in a public repository because of their large size but are available from the lead contact on request.

EXPERIMENTAL MODEL AND SUBJECT DETAILS

All experiments were performed in accordance with UK Home Office regulations (Animal Welfare Act of 2006) following local ethical approval. 8-16 week old male and female VGAT-ires-Cre mice (Vong et al., 2011, Jackson Laboratory, stock 028862) and VGAT::td-Tomato mice (cre-dependent tdTomato expression in cells with vesicular GABA transporter (VGAT), Madisen et al., 2010, Ai14D, Jackson Laboratory, stock 007914) were housed with free access to food and water on a 12:12 h light:dark cycle and tested during the dark phase. For electrophysiological recordings in acute midbrain slices, male and female VGAT::tdTomato mice were injected at an age of five weeks and recorded at seven weeks. For chemogenetic control experiments, 8-12 weeks old male and female wild-type (WT) C57BL/6J mice (Charles River) were used. We detected no influence of sex on the results, and data from male and female mice were pooled.

METHOD DETAILS

Viruses

Viruses used in this study are listed here and referred to by abbreviations in the main text:
AAV1-EF1a-doublefloxed-hChR2(H134R)-EYFP-WPRE-HGHpA (ChR2, Addgene: 20298-AAV1, provided byKarl Deisseroth, 3.5×10^{13} viral genomes (vg) $\times$ ml^{-1}, diluted to 7×10^{12} vg $\times$ ml^{-1} in saline) was used for optogenetic stimulation and anatomical tracing.
AAV1-hSyn1-SIO-stGtACR2-FusionRed (stGtACR2, Addgene: 105677-AAV1, provided by Ofer Yizhar (Mahn et al., 2018), 2×10^{13} vg $\times$ ml^{-1}, diluted to 2×10^{12} vg $\times$ ml^{-1} in saline) was used for optogenetic inhibition.
AAV1-Syn-Flex-GCaMP6f-WPRE-SV40 (GCaMP6f, Addgene: 100833-AAV1, provided by Douglas Kim and the GENIE Project (Chen et al., 2013) 2.81×10^{12} vg ml^{-1}, undiluted) was used for calcium imaging.
AAV1-EF1a-FLEX-EGFP (GFP, 1×10^{14} vg ml^{-1}, diluted to 2×10^{13} vg $\times$ ml^{-1} in saline) was used for optogenetic control experiments.
AAV1-hSyn-DIO-hM4D(Gi)-mCherry (hM4Di, Addgene: 44362-AAV1, provided by Bryan Roth (Krashes et al., 2011), 1.8×10^{13} vg $\times$ ml^{-1}, diluted to 9×10^{12} vg $\times$ ml^{-1} in saline) was used for chemogenetic inhibition.
AAVretro-hSyn-Cre (1×10^{14} vg ml^{-1}, undiluted) was used to express Cre-Recombinase in cells projecting to mSC (Figure S10).
AAV1-EF1a-FLEX-mCherry (1×10^{14} vg ml^{-1}, diluted to 2×10^{13} vg $\times$ ml^{-1} in saline) was used to label vLGN cells expressing Cre after the injection of AAVretro-hSyn-Cre in mSC (Figure S10).

Surgical procedures

Prior to surgery, the analgesic Carprofen (5 mg $\times$ kg^{-1}) was administered subcutaneously. General anesthesia was induced (4%) and maintained (1% - 2.5%) with isoflurane (in oxygen, 1 l $\times$ min^{-1}). Viral vectors were delivered using pulled glass pipettes (4.2 μL glass capillaries with ID 0.53 mm, Drummond Scientific) in an injection system coupled to a programmable nanoliter injector (Nanoject III, Drummond Scientific) at approximately 4 nL $\times$ min^{-1}. Pipettes were sharpened using a microgrinder (EG-45, Narishige). Surgeries were performed on a stereotaxic frame (Model 940, Kopf Instruments). Implants were affixed using light-cured dental cement (RelyX Unicem 2, 3M) and the incision was glued (Vetbond). Viruses were injected bilaterally in the vLGN of VGAT-Cre mice (40-60 nL per side, from skull at Bregma: ML: ± 2.52 mm, AP: −2.3 mm, DV: −3.45 mm, no angle). A subset of injections also spilled into the intergeniculate leaflet (IGL), a sheet of cells between vLGN and dLGN (see Figure 1A) or the very lateral part of the zona incerta (ZI). To verify if functional results described in this study were similar for animals with and without construct expression in IGL or in ZI, datasets were manually divided in two groups according to the amount of virus contamination in IGL or ZI, respectively (see Figures S9G–S9R). Functional results did not differ when comparing animals with the most IGL or ZI contamination to those with the least IGL or ZI contamination. Furthermore, IGL only projects to the superficial gray layer in the SC (see Figure S9B), while ZI strongly projects only to the lateral part of the SC (see Figure S9A; Oh et al., 2014). Labeled axons were restricted to mSC in our experiments and in many animals with strong behavioral effects of vLGN manipulation, very little axon labeling was visible in the IGL projection layer in mSC (see e.g., Figure S8A). Neither the lateral SC, nor the superficial layer of the SC were targeted with photometry recordings or optogenetic axon stimulation experiments as fibers were located in deeper layers (Figures S3A and S8A). Moreover, calcium activity in IGL (from an animal with GCaMP expression nearly exclusively in IGL) is strikingly different from activity observed in vLGN and vLGN axons in mSC (Figures S9C–S9F, compared with Figures S3G and S3I). We attempted to express viral constructs for manipulating mSC-projecting vLGN neurons using retrograde tracers. However, in our hands, retrograde viruses such as retroAAV (Tervo et al., 2016) or canine adenovirus (CAV) worked very well for cortical projection neurons (Figure S10A), but were ineffective for the vLGN-SC pathway. retroAAV injected into SC labeled only a very small fraction of SC-projecting vLGN neurons (Figure S10B), insufficient for significant manipulation of neuronal activity. This is consistent with previous literature showing cell-type specific tropisms and ineffective labeling of subcortical pathways in retroAAV (Sun et al., 2019).

Two 200 μm optic fibers (FC_200/245-0.37_4.5mm_SMR_FLT, Doric Lenses) were either implanted bilaterally over vLGN without penetrating the optic tract (from skull at Bregma, ML: ± 2.6 mm, AP: −2.3 mm, DV: −3.18 mm, 20 degrees lateral to medial) or a single 400 μm optic fiber (MFC_400/475-0.53_4.5mm_SMR_FLT, Doric Lenses) was placed in between SC hemispheres (from skull at Bregma, ML: 0 mm, AP: −4.1 mm, DV: −1.7 mm, 30 degrees posterior to anterior) for optogenetic manipulations, or just lateral of the midline (from skull at Bregma, ML: 0.2 - 0.5 mm, AP: −4.1 mm, DV: −1.7 mm, 30 degrees posterior to anterior) for photometry recordings. For photometry recordings of vLGN cell bodies, a single 400 μm optic fiber (MFC_400/475-0.53_4.5mm_SMR_FLT, Doric Lenses) was implanted over vLGN without penetrating the optic tract (from skull at Bregma, ML: ± 2.6 mm, AP: −2.3 mm, DV: −3.18 mm, 20 degrees lateral to medial).

For acute extracellular electrophysiological recordings with Neuropixels probes in awake, head-fixed mice, a 200 μm optic fiber (CFMLC22L05, Thorlabs) was implanted over vLGN without penetrating the optic tract (from skull at Bregma, ML: ± 2.6 mm, AP: −2.3 mm, DV: −3.18 mm, 20 degrees lateral to medial) just after the injection of AAV-flex-ChR2. Then, a custom-made headplate was attached to the skull using Super Bond dental cement (Super-Bond, C&B). The surface of the skull over the mSC was then cleaned, the location of future recording locations (from skull at Bregma, ML: 0.2 mm, AP: −4.1 mm) was marked, and a small plastic well was cemented (RelyX Unicem 2, 3M) to the exposed skull and attached to the headplate. The well was then filled with Kwik-Sil sealant (World Precision Instruments) and closed with a plastic cap.

Optogenetic manipulation and photometry recording experiments were performed 12 to 21 days post-surgery, and *in vivo* electrophysiological recordings 10 to 14 days post-surgery. Chemogenetic inactivation experiments were performed 4 to 6 weeks after surgery.

Histology

At the end of experiments mice were euthanized with an intraperitoneal pentobarbital (IP, 80 mg × kg^{-1}) injection and transcardially perfused (0.01 M phosphate buffered saline (PBS), followed by 4% paraformaldehyde in PBS).

For determining projection targets of vLGN, and for histological confirmation of injection sites and fiber placement, brains were embedded in 5% agarose (A9539, Sigma) and imaged using a custom-built serial-section two-photon microscope (Mayerich et al., 2008; Ragan et al., 2012). Coronal slices were cut at a thickness of 50 μm using a vibratome (Leica VT1000), and optical sections were acquired every 12.5 μm. Scanning and image acquisition were controlled by ScanImage v5.5 (Vidrio Technologies, USA, Pologruto et al., 2003) using a custom software wrapper for defining the imaging parameters (https://zenodo.org/record/3631609).

To quantify the proportion of GABAergic neurons in vLGN, agarose-embedded VGAT::tdTomato brains were sliced at a thickness of 50 μm using a vibratome (7000smz-2, Campden Instruments Ltd UK) and kept in 0.01M PBS for 30 minutes. For immunohistochemical staining, sections were first blocked in a solution containing 10% Donkey Serum (Millipore, UK), 5% Bovine Serum Albumine (BSA, Cambridge Bioscience) and 0.3% Triton X (VWR International Ltd) in 0.01M PBS for two hours at room temperature, on a shaker. The blocking solution was replaced by a primary antibody solution containing 1:100 Mouse anti-NeuN antibody (monoclonal clone A60, MAB377 Sigma), 1% BSA and 0.3% Triton in 0.01M PBS. Sections were incubated in this solution for 16 hours while placed on a shaker at 4°C. They were then washed three times for 15 minutes with 0.01M PBS, before being incubated on a shaker for two hours at room temperature in a secondary antibody solution containing 1:500 Donkey anti-Mouse Alexa Fluor 647 (A32787, ThermoFisher Scientific), 5% Donkey Serum, 1% BSA and 0.3% Triton. After secondary incubation, slices were washed an additional four times for 15 minutes and mounted with ProLong Glass Antifade Mountant (P36980, ThermoFisher Scientific). Images of 1024 × 1024 pixels were acquired using a confocal microscope (TCS SP8 Laser Scanning Confocal, Leica) with a 25x water-immersion objective. The proportion of GABAergic neurons in vLGN was determined manually on single-plane images. Due to dense tdTomato-labeled neuropil in vLGN, we adopted conservative criteria for quantification. VGAT-negative neurons were defined as showing NeuN staining accompanied by a clear lack of tdTomato fluorescence on the tdTomato channel. VGAT-tdTomato-positive neurons were required to show clear, in-focus NeuN staining as well as a fluorescent soma, well-demarcated from the surrounding neuropil in the tdTomato channel.

Electrophysiological slice recordings

Preparation of acute midbrain slices

Five-week-old male and female VGAT::tdTomato mice were injected with AAV-flex-ChR2 in vLGN. After two weeks, mice were sacrificed by decapitation following anesthesia with isoflurane. Brains were quickly removed and immediately immersed in ice-cold slicing solution containing (in mM): 87 NaCl, 2.5 KCl, 26 $NaHCO_3$, 1.25 NaH_2PO_4, 10 glucose, 50 sucrose, 0.5 $CaCl_2$, and 3 $MgCl_2$, with an osmolarity of ~280 mOsm, and constantly bubbled with carbogen (95% O_2 and 5% CO_2) for a final pH of 7.3. Acute coronal slices of 250 μm thickness were prepared at the level of the SC (−0.8 to 0.2 mm from lambda) and the vLGN injection site using a vibratome (Leica VT1200 S). Slices were collected and transferred to a recovery chamber containing slicing solution and submerged at near-physiological temperature (35°C) for 30 minutes, constantly bubbled with carbogen (95% O_2 and 5% CO_2). Slices were subsequently transferred to a different recovery chamber and submerged in artificial cerebrospinal fluid (aCSF) solution containing (in mM): 125 NaCl, 2.5 KCl, 26 $NaHCO_3$, 1 NaH_2PO_4, 10 glucose, 2 $CaCl_2$, and 1 $MgCl_2$, with an osmolarity of ~295 mOsm and constantly bubbled with carbogen (95% O_2 and 5% CO_2) for a final pH of 7.3. Slices were allowed to further recover at room temperature (19 - 23°C) for at least 30 more minutes prior to electrophysiological recordings.

Recording electrodes

Pipettes were pulled from standard-walled filament-containing borosilicate glass capillaries (Harvard Apparatus, 1.5 mm OD, 0.85 mm ID) using a vertical micropipette puller (PC-10 or PC-100, Narishige) to a final resistance of 4 - 6 MΩ. Pipettes were backfilled with potassium methanesulfonate based solution containing (in mM): 130 $KMeSO_3$, 10 KCl, 10 HEPES, 4 NaCl, 4 Mg-ATP, 0.5 Na_2-GTP, 5 Na-Phosphocreatine, 1 EGTA, biocytin (1 mg × mL^{-1}), with an osmolarity of 294 mOsm, filtered (0.22 μm, Millex) and adjusted to pH 7.4 with KOH. Pipettes were inserted into the pipette holder of a patch-clamp headstage (EPC 800, HEKA), controlled by an electrical micromanipulator (PatchStar, Scientifica). A silver wire coated with silver chloride (AgCl) was present inside the pipette and was in contact with the intracellular solution, and an Ag-AgCl pellet electrode (Warner Instruments, E206, 2.0 mm D) was used as a bath electrode.

Data acquisition

Whole-cell recordings were performed with an EPC 800 amplifier (HEKA). Data were sampled at 25 kHz, low-pass Bessel filtered at 5 kHz, digitised with 16-bit resolution using a PCIe-6353 board (National Instruments), and recorded in LabVIEW using custom software. For recordings, slices were transferred to a submerged chamber and perfused with aCSF constantly bubbled with carbogen (95% O_2 and 5% CO_2). The solution was perfused at a flow rate of 2 - 3 mL × min^{-1} with a peristaltic pump (PPS2, MultiChannel Systems) and temperature was kept at 32 - 34°C. Expression of ChR2 in vLGN and presence of ChR2-positive terminals in SC were confirmed prior to recordings based on fluorescence from YFP expression using LED illumination (pE-100, CoolLED) at a wavelength of 490 nm.

The SC was located using a 4x objective (Olympus) and cells were visualized with oblique illumination on an upright SliceScope Pro 1000 (Scientifica) using a 60x water-immersion objective (Olympus). Target cells were identified based on fluorescence from tdTomato expression using LED illumination (pE-100, CoolLED) at a wavelength of 565 nm. ChR2 was activated with wide-field 490-nm LED illumination (pe-100, CoolLED, 1-ms pulse length, five pulses at 20 Hz, maximum light intensity = 6.2 mW). Responses were recorded in voltage-clamp, with cells held around, above and below the approximate expected chloride reversal potential. The holding potentials used were between −40 mV and −90 mV so that evoked currents could be observed to flip from outward to inward. Input resistance (*R*in) was monitored continuously throughout the experiment. Upon termination of the recording, the anatomical location of the neuron within the slice was recorded using a 4x objective (Olympus) for future analysis. For the bath-applied drug recordings, kynurenic acid (2 mM, Sigma Aldrich) or picrotoxin (50 uM, Bio-Techne) were added to the recording aCSF and allowed to perfuse into the recording chamber for at least five minutes before the start of the recordings.

Experimental set-up of behavioral procedures

Experiments assessing escape behavior in response to looming stimuli were performed in a custom-made transparent acrylic arena (Figure 1G; L: 80 cm × W: 26 cm × H: 40 cm). The opaque floor was white to improve automated tracking. A custom-made, red-tinted acrylic shelter (L: 14 cm × W: 14 cm × H: 14 cm) was placed on one end of the arena, while threat stimuli were presented on the other end, in a 30-cm long threat zone. A small empty Petri dish was anchored in the threat zone to promote exploration. The open field test and recordings of visual responses in freely moving animals were performed in a custom-made white acrylic arena (Figure 2A; L: 40 cm × W: 40 cm × H: 30 cm). The opaque floor was white to improve automated tracking. Risk-avoidance behavior was also assessed in a custom-made gray acrylic elevated plus maze (Figure 2C; EPM). It consisted of two opposing open arms (without walls, L: 30 cm, W: 5 cm) and two opposing closed arms (with walls, L: 30 cm, W: 5 cm, wall H: 5 cm). All four arms extended from a center square (5 cm by 5 cm) to form a plus shape. The maze was positioned on an acrylic pedestal (H: 20 cm).

All arenas were placed in a large light-proofed and sound-deadening box (L: 120 cm × W: 100 cm × H: 120 cm, LS Fabrications). An ultra-short throw DLP projector (HF85JA, LG) was mounted on an custom-made aluminum scaffold (Bosch Rexforth) within the box and back-projected via a mirror onto a suspended horizontal screen (60 cm above arena floor, 100 cm × 80 cm; ‘100 micron drafting film’, Elmstock) illuminated by three infrared LED illuminators (Jcheng Security). For all behavioral assays, the screen was kept at a constant background luminance level of 9 cd × m^{-2}.

Behavior was recorded with a near-IR GigE camera located above the arena (acA1300-60 gmNIR, Basler) at 50 frames per second through a varifocal lens (H2Z0414C-MP, Computar) and 780nm long-pass filter (Thorlabs). Each camera frame was triggered and synchronized by the LabVIEW-based (National Instruments) Mantis software (mantis64.com), through a multifunction I/O acquisition board (USB-6343, National Instruments) with other input and output channels. The analog 50 Hz output trigger was saved as analog input for post hoc alignment.

Behavioral protocols

For escape experiments, mice were placed in the escape arena and given time to explore the new environment (10 minutes in optogenetic and chemogenetic manipulation experiments, 30 minutes in calcium photometry experiments) before the first stimulus was presented. Stimulation was manually triggered when the mouse reached the threat zone. The stimulus was only triggered when the mouse was facing and walking toward the threat zone and was not near the walls of the arena. Trials in which the mouse looked down or faced away from the threat zone were excluded. Stimuli were only presented with a probability of 25 to 50% upon entry into the threat zone. For all experiments, the inter-stimulus interval was at least two minutes. A typical experiment lasted 30 to 150 minutes.

For optogenetic manipulation experiments, looming stimuli of different contrasts were presented in a randomized order. Laser and non-laser trials of the same stimulus contrast were always presented as paired trials, in a randomized but consecutive order.

CellPress
OPEN ACCESS

Optogenetic manipulation experiments during sound-evoked escapes were organized similarly. Sound levels were adjusted in order to induce escape in roughly 50% of trials, and laser and non-laser trials of the same sound level were always presented as paired trials, in a randomized but consecutive order.

For photometry experiments, looming stimuli were again only presented upon entry into the threat zone with 25 – 50% probability. Sessions began with the presentation of 3 - 6 high-contrast looming stimuli (99%) to sensitize the animal before presenting several intermediate-contrast looming stimuli (50% - 60%). To habituate the animals, stimuli of various contrasts were used. First, animals were presented with low-contrast stimuli (20% - 40%) until they reliably stopped escaping. The stimulus contrast was then progressively increased until animals stopped escaping to stimuli of most contrasts.

For chemogenetic manipulation experiments, a fixed sequence of 10 stimuli was used (40%, 20%, 60%, 40%, 20%, 40%, 20%, 60%, 40%, 20%), allowing the comparison of escape probability across mice. Mice were placed in the escape arena 30 minutes after an IP injection of either saline or Clozapine N-Oxide (CNO). The experiment was stopped after the presentation of the 10th stimulus or 50 minutes after the presentation of the first stimulus, whichever happened first. Risk-avoidance behavior in the open field was assessed by placing an animal into the corner of the open field arena 40 minutes after an IP injection of either saline or CNO (5 mg $\times$ kg^{-1}, SML2304, Sigma Aldrich). Mice were then allowed to freely explore the arena for five minutes.

Risk-avoidance behavior in the elevated plus maze was assessed by placing the animal into a closed arm of the maze 40 minutes after an IP injection of either saline or CNO (5 mg $\times$ kg^{-1}). Mice were then allowed to freely explore the maze for 15 minutes.

Visual stimuli

Visual stimuli were created and presented through custom-made scripts in MATLAB (Mathworks), using the Psychophysics Toolbox extensions (Brainard, 1997; Pelli, 1997; Kleiner et al., 2007). Visual stimuli were triggered through the Mantis software (mantis64.com) and projected onto the screen. A switchable gain, Si-amplified photosensor (PDA100A2, Thorlabs) was used to record the stimuli presented on the screen for post hoc alignment. Visual looming stimuli consisted of a sequence of three dark expanding circles centered over the threat zone. The radius of the spot expanded linearly at 55 deg s^{-1} from 1 deg at onset to 20.8 deg in 360 ms. The spot was then kept at constant size for 250 ms followed by a 500 ms inter-stimulus interval. The contrast (c) of the stimulus was varied by maintaining the background luminance (I_B) constant at 9 cd $\times$ m^{-2} and changing the luminance of the spot (I) according to a negative Weber contrast law: $c = -(I - I_B)/I_B$. This ensured contrasts were always defined by positive values. Stimulus contrasts ranged from 10% to 99%.

Visual responses in freely moving animals were assessed by pseudo-randomly displaying full-field stimuli of five logarithmically spaced luminance levels (1 cd $\times$ m^{-2}, 3 cd $\times$ m^{-2}, 9 cd $\times$ m^{-2}, 27 cd $\times$ m^{-2}, 81 cd $\times$ m^{-2}) on the screen above the mouse. Each luminance level was displayed for 10 s and repeated 40 times per session. A background luminance level of 9 cd $\times$ m^{-2} was displayed for 10 s in between trials.

Auditory stimuli

Auditory stimuli were created and presented through the Mantis software (mantis64.com) and consisted of 15 kHz pure tones lasting three seconds. Sounds were amplified (PLA500, Pulse) and delivered through an ultrasound speaker (L60, Pettersson) centered at the end of the arena at a height of 50 cm. Sound pressure levels ranged from 65 to 90 dB of Sound Pressure Level (dB SPL). The analog output driving the amplifier was also recorded as analog input for post hoc alignment. Auditory stimuli were only presented upon entry into the threat zone with 25 to 50% probability. For photometry experiments with auditory threat stimuli (Figure S4G), sessions began with the presentation of 3 - 6 90 dB SPL stimuli to sensitize the animal. To habituate the animals, the sound pressure level was first decreased to 65 dB SPL and then progressively increased.

Optogenetic manipulation

Laser stimulation protocols were created and presented through the Mantis software (mantis64.com). For stimulation of ChR2-expressing neurons the laser was pulsed for four seconds at 20 Hz using 5-ms pulses. Laser stimulation of stGtACR2-expressing neurons consisted of a single four-second-long continuous square pulse. Analog signals drove a 473 nm laser (OBIS 473nm LX 75mW LASER SYSTEM, Coherent) coupled to a 200 μm fiber patch cable through an achromatic fiberport (Thorlabs). Light intensity was divided at the level of the rotary joint (FRJ_1x2i_FC-2FC_0.22, Doric Lenses) in two equal halves. Two identical 200 μm fiber patch cables (MFP_200/220/900-0.37_1m_FC-SMC, Doric Lenses) were connected to the two outputs of the rotary joint and connected to the implanted cannulae through a magnetic connector.

For optogenetic stimulation of vLGN axons in SC, only one patch cord was used to connect to the single cannula in mSC. Peak power was measured at the tip of the patch fibers before each experiment. The analog output driving the laser was also recorded as analog input for posthoc alignment. During laser trials, the four-second laser stimulation started 250 ms before the onset of the visual or auditory stimulus.

For experiments testing if vLGN stimulation differently affected escape initiation and escape execution (Figure 3N), the four-second laser stimulation was initiated after the onset of the high-contrast looming stimulus. For each trial, the laser onset was manually adjusted in a range from 80 ms to 1.5 s after looming stimulus onset. The time of laser onset was recorded for post hoc alignment and analysis.

For optic fibers over vLGN, the peak laser power on each side was 10 mW. For laser stimulation of vLGN axons in SC, the peak power at the tip of the single fiber was 20 mW. To control for light-induced artifacts on escape behavior, laser stimulation was performed both in animals with GFP instead of an optogenetic construct expression in GABAergic vLGN neurons, and in animals without fluorescent construct expression in vLGN. Results were similar and therefore pooled (Figures S6F, S6G, S8B, and S8C).

Fiber photometry experiments

Optics

A 473nm LED (CLED_473, Doric Lenses) and a 405nm LED (CLED_405, Doric Lenses) were used to excite GCaMP at its calcium-dependent and isosbestic wavelength, respectively (Lerner et al., 2015). The light was focused into a single output fiber through a six-port minicube (iFMC6_AE(405)_E1(460-490)_F1(500-540)_E2(555-570)_F2(580-680)_S, Doric Lenses) connecting a pigtailed rotary joint (FRJ_1x1_PT_400/430/LWMJ-0.57_1m_FCM_0.15m_FCM). A 400 μm fiber patch cable connected the rotary joint to the implanted cannula through its slim magnetic connector (MFP_400/460/1100-0.48_0.8m_FC-SMC). Peak power amplitude at the tip of the patch cord was between 500 μW and 550 μW for all experiments. An integrated fluorescence detector head (part of iFMC6, Doric) converted the detected fluorescence signals into an analog output signal.

Data acquisition

Data was continuously acquired and synchronized through a USB-driven PyPhotometry board (Akam and Walton, 2019). The python (https://www.python.org) based open source software PyPhotometry was used to drive the 473 nm and 405 nm LEDs at roughly 100 mA each in strobing mode (Akam and Walton, 2019). The software sequentially recorded and temporally aligned the fluorescence evoked by alternating stimulation with 473 nm and 405 nm light to obtain separate time-varying traces of calcium-dependent and isosbestic GCaMP fluorescence signals. The board was also used to record the analog photometry signals at 120 Hz and the digital 50 Hz pulses driving the camera as input for post hoc alignment.

Extracellular electrophysiological recordings

Experimental set-up

Prior to the recording session, mice were habituated to head-fixation for several minutes over two to three days. On the day of the recording, mice were briefly anesthetized with isoflurane (1% - 2.5% in oxygen, 1 l × min^{-1}). The analgesic carprofen (5 mg × kg^{-1}) was subcutaneously injected and the previously applied Kwik-Sil sealant was removed. A small (1 - 1.5 mm diameter) craniotomy was made over the mSC (from skull at Bregma, ML: 0.2 - 0.5 mm, AP: −4.1 mm) without damaging the confluence of sinuses. The well was then re-filled with Kwik-Sil sealant (World Precision Instruments) and closed with its plastic cap. Mice were then head-fixed over a custom-made styrofoam cylinder after having recovered from surgery for 1 - 2 hours. The sealant was removed and the well around the craniotomy filled with cortex buffer (in mM, 125 NaCl, 5 KCl, 10 Glucose monohydrate, 10 HEPES, 2 $MgSO_4$ heptahydrate, 2 $CaCl_2$ adjusted to pH 7.4 with NaOH). A silver wire was placed in the bath for referencing. Extracellular spikes were recorded using a single-shank Neuropixels silicon probe (Phase 3A, option 3, 384 channels) connected via a dedicated cable, head-station and base-station to a Xilinx Kintex 7 FPGA board, which was accessed via Ethernet. Prior to insertion, the probe was coated with DiI (1 mM in isopropanol, Invitrogen) for post hoc histological alignment. The probe was slowly inserted into the mSC to a depth of 3 mm using a micromanipulator (Sensapex) and left in place for at least 30 minutes before the start of the recording session. Data were acquired using spikeGLX (https://github.com/billkarsh/SpikeGLX, Janelia Research Campus), high-pass filtered (300 Hz), and sampled at 30 kHz. A LCD monitor (U2715H, Dell, 60 Hz refresh rate) was placed above and in front of the mouse at an angle of 45 degrees at a distance of 20 cm from the left eye. Monitor position was optimized to maximize visual responses to stimuli displayed in the center of the monitor. Two stereo speakers (Z120, Logitech) were placed in front of the mouse, at a distance of 30 cm. In five out of six mice, two separate recordings at different locations within mSC were performed, a single recording was performed in one animal.

Optogenetic manipulation

Laser stimulation protocols were created and presented through custom-made scripts in MATLAB (Mathworks), using the Psychophysics Toolbox extensions (Brainard, 1997; Pelli, 1997; Kleiner et al., 2007). The laser was pulsed for four seconds at 20 Hz using 5-ms pulses, triggered by a Pulse Pal pulse train generator (Open Ephys) driving a 473 nm laser (OBIS 473nm LX 75mW LASER SYSTEM, Coherent) coupled to a 200 μm fiber patch cable through an achromatic fiberport (Thorlabs). The patch cable was connected to the implanted optic fiber over vLGN. The peak laser power at the tip of the fiber was 10 mW.

Stimulation protocols

All visual and auditory stimuli were created and presented through custom-made scripts in MATLAB (Mathworks), using the Psychophysics Toolbox extensions (Brainard, 1997; Pelli, 1997; Kleiner et al., 2007).

Looming stimuli were created as previously described. Looming stimuli of 20%, 40%, 60%, and 99% contrast were presented. Looming stimuli were pseudorandomly displayed in blocks of 8 stimuli (four contrast levels with and without laser stimulation), repeated 25 times. The interstimulus interval between two consecutive trains of looms was 30 s. The four-second laser stimulation was started 250 ms before the onset of the first loom.

Pure tones of 15 kHz sound frequency and two different sound pressure levels (70 dB and 80 dB) were presented for three seconds with five-second inter-stimulus interval. Each stimulus was repeated 20 times.

CellPress
OPEN ACCESS

Square-wave gratings of 4 Hz temporal frequency and 0.05 cycles per degree spatial frequency were presented at four orientations, drifting in eight directions (0 to 360 degrees in 45-degree increments). Each grating was presented for two seconds with a two-second inter-trial interval. Each stimulus was repeated 40 times.

Forty blank laser stimulation trials were randomly interleaved during the grating presentation protocol. In those trials, the laser was on for 1.5 s while neither visual nor auditory stimuli were presented.

QUANTIFICATION AND STATISTICAL ANALYSIS

Electrophysiological slice recordings

A cell was considered connected if it responded consistently across trials with a short and reliable latency after light onset. Synaptic conductance was computed using custom-made scripts in python 3.7 by estimating, with a linear fit, the peak magnitude of evoked currents against the holding potential: $I = g(V_m - E_{reversal})$ and obtaining the gradient of the fit for each cell. For cells where g and $E_{reversal}$ could not reliably be determined in this way, the mean reversal potential of all other cells was taken as $E_{reversal}$ and used to calculate g at each holding potential of the cell, the average of which was used as the estimate of synaptic conductance. The relationship between cell layer and inhibitory synaptic conductance was determined by linear regression using the *fitlm* function in MATLAB.

Pre-processing of behavioral data

Raw videos of mouse behavior were extracted using a dedicated converter (mantis64.com) and saved as AVI files. The analog input channels (sound, photodiode, laser, and camera trigger) were extracted in MATLAB (MathWorks). All analog input channels were recorded at 25000 samples per second, while the camera was triggered at 50 Hz. Using custom-made scripts in MATLAB, time points of visual and auditory stimuli onsets, as well as the laser onsets, were extracted and matched to the corresponding video camera frame.

Behavior extraction

To extract behavior we used a recently developed deep neural network approach (DeepLabCut; Mathis et al., 2018). A network was trained with 100 manually annotated frames of an initial dataset, and then re-trained with 25 new frames each from two additional datasets from different mice. The resulting labeled videos were manually inspected for quality control. This network was used for all behavioral classifications, and the XY-position of body-center, head, nose, and ears within the arena was extracted. XY-positions were loaded and processed in MATLAB. The position of the animal was defined as the body-center position. The head-direction of the animal was computed as the vector between the nose and the head (0 degrees: mouse facing toward the "threat zone"; ± 180 degrees: mouse facing toward the shelter). Instantaneous speed was computed as the Euclidean distance between two positions at consecutive time points. Angular velocity was computed as the discrete derivative at two consecutive time points (taking into account circular boundary conditions). Prior to computing the speed and angular velocity, the position and head direction vectors were smoothed by a 100 ms running average filter to avoid amplifying noise when computing derivatives. The ears were tracked to increase the stability of the network with more co-dependent markers.

Behavior analysis

For all experiments, data analysis was performed in custom-written routines in MATLAB (Mathworks). In the open field test, the 40 cm by 40 cm arena was divided into a 5 by 5 grid for analysis. The center of the open field was defined as the inner 3 by 3 grid, representing 36% of the surface area. Using the automatically extracted markers, the amount of time spent within the center was quantified and divided by the total length of recording (five minutes).

For the elevated plus maze, analysis was performed manually. An entry was defined as the time point at which an animal entered completely (with all four limbs) into an open or closed arm from the center regardless of whether it had been in an open or closed arm immediately prior. The relative number of entries into an open arm was defined as the ratio between the number of entries into an open arm and the number of entries in any arm. Similarly, the relative time in an open arm was defined as the time the animal's entire body was in an open arm divided by the total time of recording (15 minutes). For all experiments, data analysis was performed in custom-written routines in MATLAB (Mathworks). A successful escape was defined as reaching the shelter within five seconds of stimulus onset. To qualify as either an evoked or spontaneous escape, the mouse needed to be at least 10 cm away from the shelter, to be facing toward the threat zone (head direction at an angle less than 60 degrees from the front), and to reach the shelter in less than 2.5 s after escape onset. Escape onset was defined as the last time point before the onset of body rotation leading to an escape, defined as an angular velocity > 100 deg/s For a given stimulus contrast and laser condition, the evoked escape probability was defined as the ratio between successful escapes and the total number of stimulus presentations. The spontaneous escape probability was defined as the probability of a fast return to the shelter (within 2.5 s after escape onset) without encountering a looming stimulus each time the animal approached the threat zone from the direction of the shelter. Non-escape returns (Figure S4H) were defined as returns to the shelter in cases where no stimulus was presented and animals did not spontaneously escape. Additionally, the maximum speed in the two seconds prior to reaching the shelter could not exceed $40 \text{ cm} \times s^{-1}$.

In all plots, the mean or median escape probability across mice is shown, unless stated otherwise. Psychometric contrast curves (Figure 3I and 5C) of escape probability were computed using a logistic regression model across mice using the *fitglm* function in MATLAB.

The time spent in the shelter was computed as the time difference between the entry into and the exit from the shelter. To obtain the relative time spent in shelter, the sum of all periods within the shelter were divided by the total time.

The peak running speed during escape was computed as the maximum speed in a 3.75 s window from stimulus onset to laser offset in trials with successful escapes. All trials were manually inspected for stimulus-evoked freezing, defined as complete absence of motion for at least two seconds during the stimulus presentation. Freezing was never observed in response to high-contrast looming stimuli and very rarely (7 out of 266 trials) in response to intermediate- or low-contrast stimuli. The total displacement after looming stimulus presentation was calculated as the total distance traveled by the mouse in non-escape trials starting 500 ms after stimulus onset until the end of stimulus presentation.

In experiments testing if vLGN stimulation differently affected escape initiation and escape execution (Figure 3N), laser stimulation was considered to have started before escape initiation if the animal turned by less than 30 degrees between the looming stimulus onset and the laser onset. Laser stimulation was considered to have started after escape initiation if the animal had turned by more than 60 degrees between the looming stimulus onset and the laser onset. Trials in which the animal was less than 10 cm from the shelter at the time of laser stimulation were removed from analysis.

Fast body rotations not part of an escape (Figures S3G, S9C, and S9E) were body rotations of at least 250 deg × s^{-1} for at least 100 ms. Fast running bouts (Figures S3I, S9D, and S9F) were running episodes with a maximum speed of at least 35 cm × s^{-1}. For these analyses escape and non-escape trials were differentiated by whether the animal reached the shelter in less than 2.5 s after rotation onset.

For photometry experiments, an animal was considered naive before, and experienced after, being presented with a looming stimulus for the first time. After several initial high-contrast (99%) and intermediate-contrast (50% - 60%) looming stimuli, the habituation protocol was started and the animals were no longer classified as experienced. An animal was considered habituated when it did not escape to looming stimuli of intermediate contrast (50% - 60%) during three consecutive trials. For photometry experiments with auditory stimuli (Figure S4G), a similar approach was used. After several initial loud (90 dB SPL) threat stimuli, the habituation protocol was started and the animals were no longer classified as experienced. An animal was considered habituated when it did not escape to auditory stimuli of 85 dB SPL during three consecutive trials.

Calcium imaging

Raw fluorescence traces were extracted and imported into MATLAB using software generously provided by Thomas Akam (https://pyphotometry.readthedocs.io/en/latest). Raw signals of calcium-dependent (GCaMP excited at with 473 nm light) and isosbestic fluorescence (GCaMP excited with 405 nm light) were filtered with a slow 10-minute running-median filter and subtracted from the original traces to correct for slow changes such as photobleaching. The isosbestic signal should not contain any calcium-dependent signals, but is sensitive to transient changes in fluorescence not due to changes in the calcium concentration, for instance signals due to motion artifacts such as bending of or tension within the patch-cord. Such signals are shared between the two channels. To correct for such non-calcium dependent signals and artifacts, the isosbestic fluorescence trace was linearly fitted to the calcium-dependent GCaMP fluorescent signal and subtracted, providing a measure of relative change in fluorescence (ΔF/F). To be able to compare calcium activity across sessions and mice, z-scored ΔF/F was computed by subtracting the mean value of the motion-corrected calcium-dependent GCaMP signal of the session and dividing the resulting trace by the standard deviation. Fluorescence traces, acquired at 120 Hz, were then re-sampled at 50 Hz, and aligned to camera frames via the digital input signal. Displayed fluorescent traces are down-sampled to 25 Hz.

To compute ON responses during recordings of visual responses in freely moving animals (Figures 1F and S3C), the average difference between the z-scored ΔF/F in the second after and the second before stimulus onset was computed. Similarly, OFF responses were computed comparing the z-scored ΔF/F in the second after stimulus offset to the z-scored ΔF/F in the second before stimulus offset. As the stimulus luminances were logarithmically spaced (1 cd × m^{-2}, 3 cd × m^{-2}, 9 cd × m^{-2}, 27 cd × m^{-2}, 81 cd × m^{-2}), the x axis in Figures 1F and S3C shows logarithmic luminance change: Luminance change $= \log_3\left[\text{luminance}\left(cd \times m^{-2}\right) / \left(9\, cd \times m^{-2}\right)\right]$.

To determine if the increase in z-Scored ΔF/F after looming stimulus onset was evoked by the visual stimulus or related to the mouse's escape, we separately analyzed trials in which mice initiated escape later than 0.5 s after stimulus onset (Figure S3E). The z-scored ΔF/F in the arena in naive, experienced, and habituated mice (Figures 1K, 1L, S4G, and S4I) was averaged during epochs ('trials') in which the mouse approached the threat zone. Per trial, the 30 s within reaching the threat zone were considered. Activity was either averaged between 0 and 20 cm distance from the threat zone (Figures 1K and S4G) or split into 5 cm bins (Figures 1L and S4I) including the activity within the shelter before leaving (distance ≥ 40 cm). The relationship between the average calcium activity 0 - 2 s before reaching the threat zone in experienced animals, the average running speed, and average head direction (Figures S3J and S3K) were determined by linear regression using the *fitlm* function in MATLAB.

Extracellular electrophysiological recordings

Spikes were sorted with Kilosort2 (https://github.com/cortex-lab/Kilosort) and Phy (Rossant et al., 2016) using procedures as previously described (Pachitariu et al., 2016). Each unit was attributed to the channel on which the extracellular waveform had the highest amplitude. The recording depth and layer of recorded units in mSC were estimated according to the distance of the recording channel from the SC surface along the DiI track. Spike rates were computed using a causal average filter over 50 ms. Traces were

CellPress
OPEN ACCESS

shown at a sampling rate of 50 Hz. Single units with an average spontaneous firing rate of less than 5 Hz were removed in the analysis of the suppressive effect of vLGN stimulation during spontaneous activity (Figures 4G, 4H, S7B, and S7C) to ensure robust laser effect estimates.

For looming responses (Figures 4C and 4D), the average spike rates in the first three seconds from stimulus onset were normalized to the response to 99% contrast stimuli without laser stimulation. To estimate the effect of vLGN stimulation on mean mSC activity (Figures 4D and 4E) or activity in a specific mSC layer (Figure 4F), spikes from all unit types (well-isolated single units and multi units) were combined. If multiple recordings were performed in one animal (5 out of 6 mice), values were first computed independently for each recording before being averaged to obtain a single value per mouse. For mSC population spike rates, the effect of laser (Figures 4E and 4F) was defined as the average relative reduction in spiking in the first three seconds from stimulus onset during the 99% contrast stimulus with vLGN stimulation compared to the 99% contrast stimulus without laser stimulation. A positive value indicates that vLGN stimulation decreased the average spike rate.

A single unit was considered to be responsive to looms if its average response to 99% contrast stimuli without laser stimulation exceeded a given threshold within the first 500 ms after stimulus onset for at least 200 ms. This threshold was defined as two standard deviations above the mean spike rate in the 500 ms before stimulus onset. Similarly, a unit was considered to be sound-responsive if its response to 15 kHz sounds was more than two standard deviations above the baseline spike rate in the 500 ms before stimulus onset. A unit was considered to be visually-responsive to grating stimuli if its average response during grating presentation deviated by more than two standard deviations from the baseline spike rate. Similarly, a unit was considered to be significantly affected by laser, if its response to vLGN stimulation on blank trials without stimulus presentation deviated by more than two standard deviations from the baseline spike rate in the 500 ms before laser onset.

'Visual units' (Figures 4G and S7B) are all single units showing a significant response to either looming or grating stimuli regardless of their responses to sounds. These were compared to single units showing no significant response to any of the visual stimuli (non-visual units). In Figure 4H, single units showing a significant response to either looms or gratings, but not to sounds (visual-only units; also Figure S7C) were compared to single units with significantly increased activity in response to sounds without significant visual response (sound-only units). For single units, the effect of laser (Figures 4G and 4H) was calculated as the average relative change in spontaneous spike rate during 1.5 s laser stimulation compared to the 500 ms before laser onset. Only units in intermediate and deep layers were considered for this analysis. A positive value indicates that vLGN stimulation led to a decrease in the average spike rate.

Statistics

All statistical tests were performed in MATLAB (Mathworks). For each experiment, data were first tested for normality by means of a Shapiro–Wilk test. For non-paired datasets or for repeated-measures with more than two groups, each group was separately tested for normality. For paired datasets, the paired difference was tested for normality. If the null hypothesis was rejected ($p < 0.05$), non-parametric tests were used. Accordingly, for plots showing non-parametric data, the median and the interquartile range were used for display purposes. If the null hypothesis that data are normally distributed could not be rejected, parametric tests were used. Accordingly, for plots showing parametric data, the mean and the standard error of the mean were used for display purposes. For paired data, the dependent t test for paired samples and the Wilcoxon signed-rank test were used for parametric and non-parametric data, respectively. For two independent groups of samples, the independent two-sample t test and the Wilcoxon rank-sum test were used for parametric and non-parametric data, respectively. For repeated-measure designs, repeated-measures one-way analysis of variance followed by Tukey's honest significance test and Kruskal–Wallis one-way analysis of variance followed by Dunn's multiple comparison test were used for parametric and non-parametric data, respectively. Where applicable, the mean value across repeated trials within individual animals was computed prior to statistical testing across animals. All statistical tests were two-tailed and described in the figure legends. The value of n and what type of data it represented was specified for each figure in the corresponding figure legend. Significance levels were indicated by stars as follows: ns: $p \geq 0.05$, *: $p < 0.05$, **: $p < 0.01$, ***: $p < 10^{-3}$.

Neuron, Volume *109*

Supplemental information

Flexible inhibitory control of visually evoked defensive behavior by the ventral lateral geniculate nucleus

Alex Fratzl, Alice M. Koltchev, Nicole Vissers, Yu Lin Tan, Andre Marques-Smith, A. Vanessa Stempel, Tiago Branco, and Sonja B. Hofer

Supplemental Figure S1

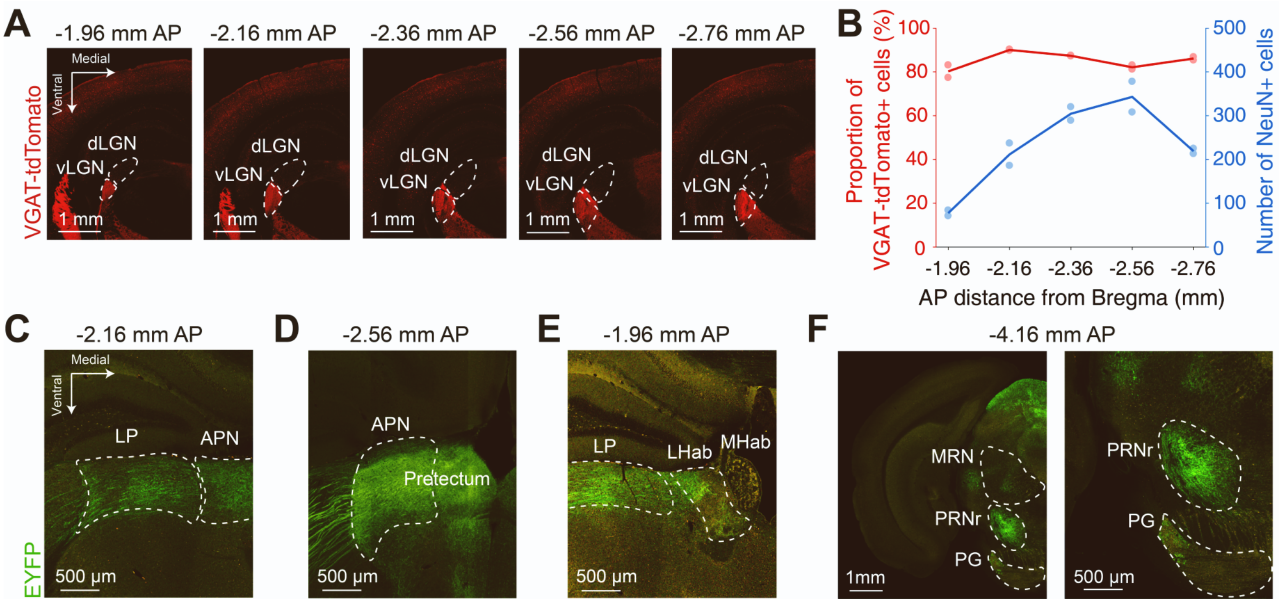

Supplemental Figure S1. vLGN VGAT expression and projection targets. Related to Figure 1.

(A) Images of tdTomato expression in VGAT-positive neurons at different positions along the anterior-posterior (AP) axis in coronal slices. **(B)** VGAT-tdTomato+ cells (red) as a proportion of all NeuN+ cells in the vLGN, and the total amount of NeuN+ cells (blue) in the vLGN at various positions along the AP axis (n = 2 mice, 20 sections). **(C-F)** EYFP-labelled GABAergic vLGN axons in different target areas in coronal slices. APN: anterior pretectal nucleus, LP: lateral posterior nucleus of the thalamus, LHab: lateral habenula, MHab: medial habenula, MRN: midbrain reticular nucleus, PG: pontine grey, PRNr: pontine reticular nucleus. In all panels, distance is in mm from Bregma.

Supplemental Figure S2

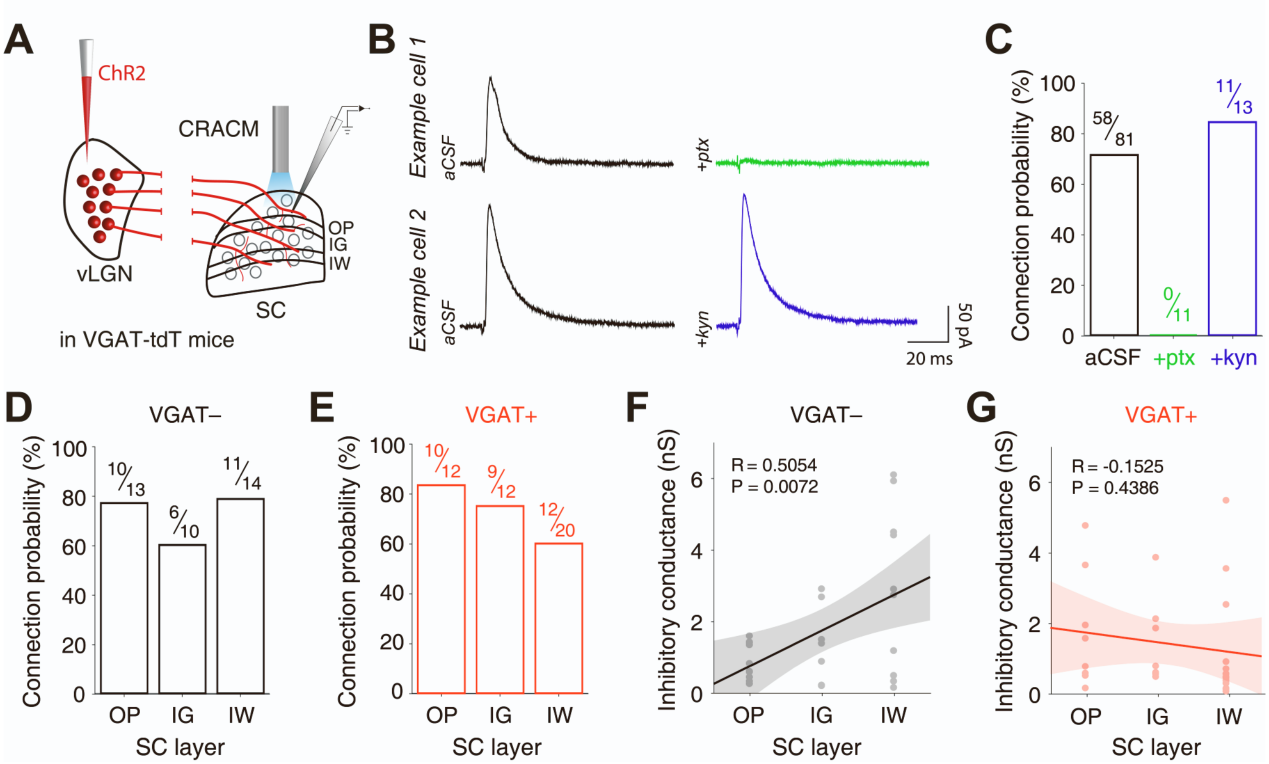

Supplemental Figure S2. Synaptic connectivity of vLGN axons in SC. Related to Figure 1.

(A) Schematic of the experimental approach: VGAT-tdTomato mice were injected in vLGN with ChR2. After two weeks, ChR2-assisted circuit mapping (CRACM) was performed in acute slices, with whole-cell recordings in different mSC layers (OP: optic layer, IG: intermediate grey, and IW: intermediate white). **(B)** Example single-cell recordings performed under control conditions (black), and after bath-application of picrotoxin (ptx, green), or after bath-application of kynurenic acid (kyn, blue) in acute brain slices. **(C)** Connection probability between vLGN axons and cells in the mSC, established in control conditions (n = 81 cells), in bath-applied picrotoxin (n = 11 cells) or in bath-applied kynurenic acid (n = 13 cells) in acute brain slices. **(D,E)** Connection probability between vLGN axons and VGAT-negative **(D)** and VGAT-positive **(E)** neurons in the mSC, split by anatomical SC layers. **(F,G)** Inhibitory synaptic conductance (nS) in VGAT-negative **(F)** and VGAT-positive **(G)** neurons within OP, IG, and IW layers of the mSC. R: Pearson correlation coefficient, P: p-value for slope different from 0 derived from a linear regression model. Shading shows 95% confidence interval of the linear fit.

Supplemental Figure S3

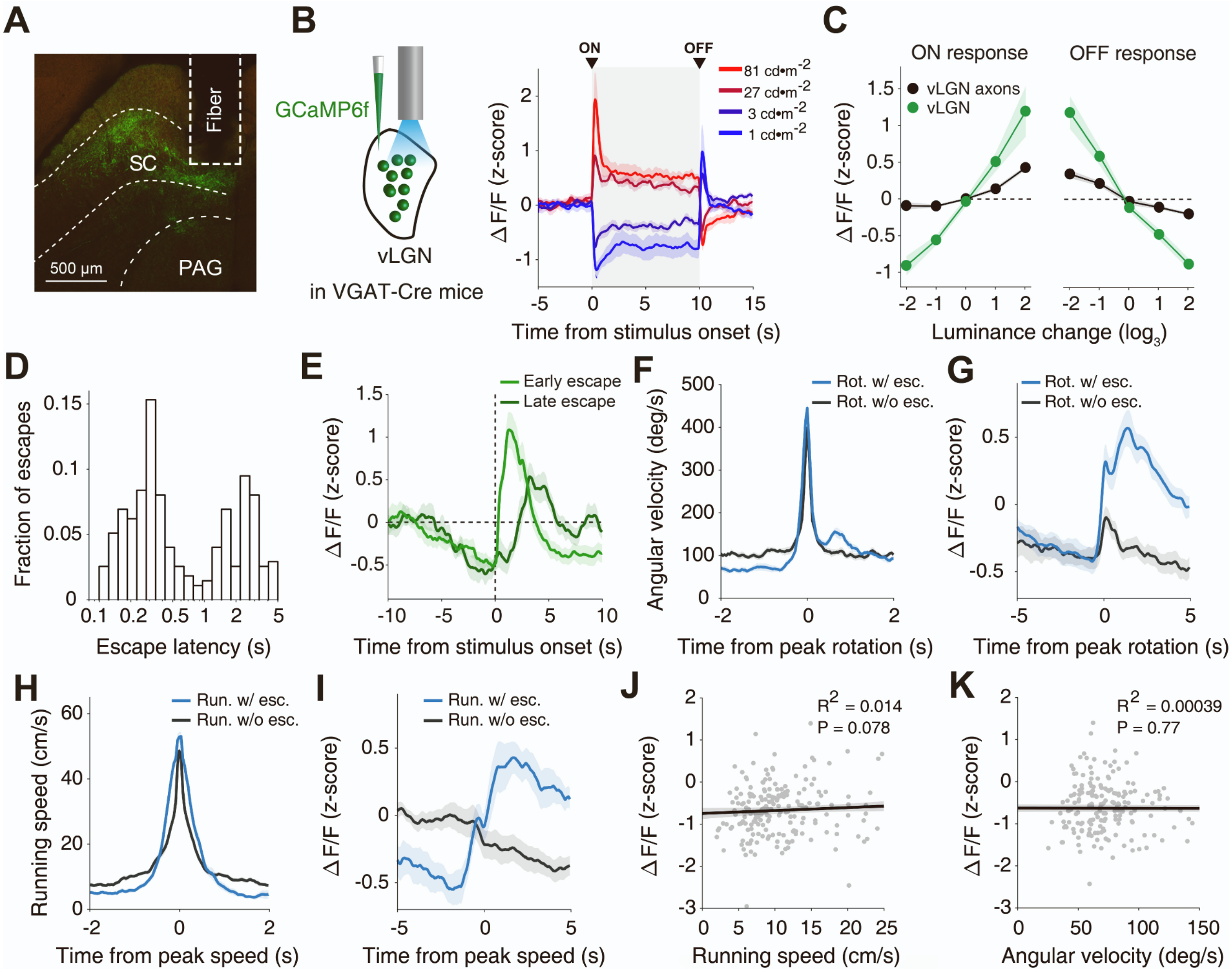

Supplemental Figure S3. Sensory and motor-related neuronal activity in vLGN and vLGN axons in mSC. Related to Figure 1.

(A) Fiber location and expression of GCaMP6f in GABAergic vLGN axons in mSC after injection of AAV-flex-GCaMP6f in VGAT-Cre mice. **(B)** Left: experimental paradigm for fiber photometry recordings of calcium signals in vLGN cell bodies after expression of GCaMP6f in vLGN GABAergic neurons. Right: mean vLGN calcium activity measured with optic fiber over vLGN in response to increases (27 cd×m^{-2}, magenta; 81 cd×m^{-2}, red) and decreases (3 cd×m^{-2}, indigo; 1 cd×m^{-2}, blue) in luminance from baseline levels (9 cd×m^{-2}). Stimulus duration is indicated by grey shading. Error bar shading represents SEM across mice; n = 6 mice. **(C)** Mean change in calcium activity due to the onset (ON response, left, difference in activity in the first second after stimulus onset compared to baseline) and the offset of the change in luminance (OFF response, right, difference in activity in the first second after stimulus offset and the last second of the stimulus) in vLGN (green, n = 6 mice) and in vLGN axons in mSC (black, n = 6 mice). Luminance change values are the base 3 logarithm of the ratio between stimulus and baseline luminance. Shading shows SEM across mice. **(D)** Distribution of time between stimulus onset and escape onset (escape latency) for escapes from looming stimuli; n = 274 trials, 9 mice. **(E)** Mean calcium activity of vLGN axons in mSC during threat-

evoked escapes aligned to stimulus onset, either with escape latency less than 0.5 seconds (early escape, light green, n = 9 mice), or more than 0.5 seconds (late escape, dark green, n = 7 mice). Shading shows SEM across mice. **(F)** Mean angular velocity in fast body rotations during escapes (blue) and in body rotations not part of an escape but with matched velocity (black) aligned to peak angular velocity. Shading shows SEM across mice; n = 9 mice. **(G)** Mean calcium activity of vLGN axons in mSC during the fast body rotations depicted in H that are part (blue) or not part (black) of an escape aligned to peak angular velocity. Shading shows SEM across mice; n = 9 mice. **(H)** Mean running speed during escape (blue) and during escape-unrelated bouts of fast running (black) aligned to peak running speed. Shading shows SEM across mice; n = 9 mice. **(I)** Mean calcium activity of vLGN axons in mSC during bouts of fast running speeds depicted in J, part (blue) or not part (black) of an escape aligned to peak running speed. Shading shows SEM across mice; n = 9 mice. **(J)** Relationship between the mean calcium activity and mean running speed of mice in the last two seconds of threat zone approach in experienced animals; n = 227 trials, 9 mice. R^2: coefficient of determination, P: p-value for slope different from 0 derived from a linear regression model. Shading around the line shows 95% confidence interval of the linear fit. **(K)** Relationship between mean calcium activity and mean angular velocity of mice in the last two seconds of threat zone approach in experienced animals; n = 227 trials, 9 mice. R^2: coefficient of determination, P: p-value for slope different from 0 derived from a linear regression model. Shading around the line shows 95% confidence interval of the linear fit.

Supplemental Figure S4

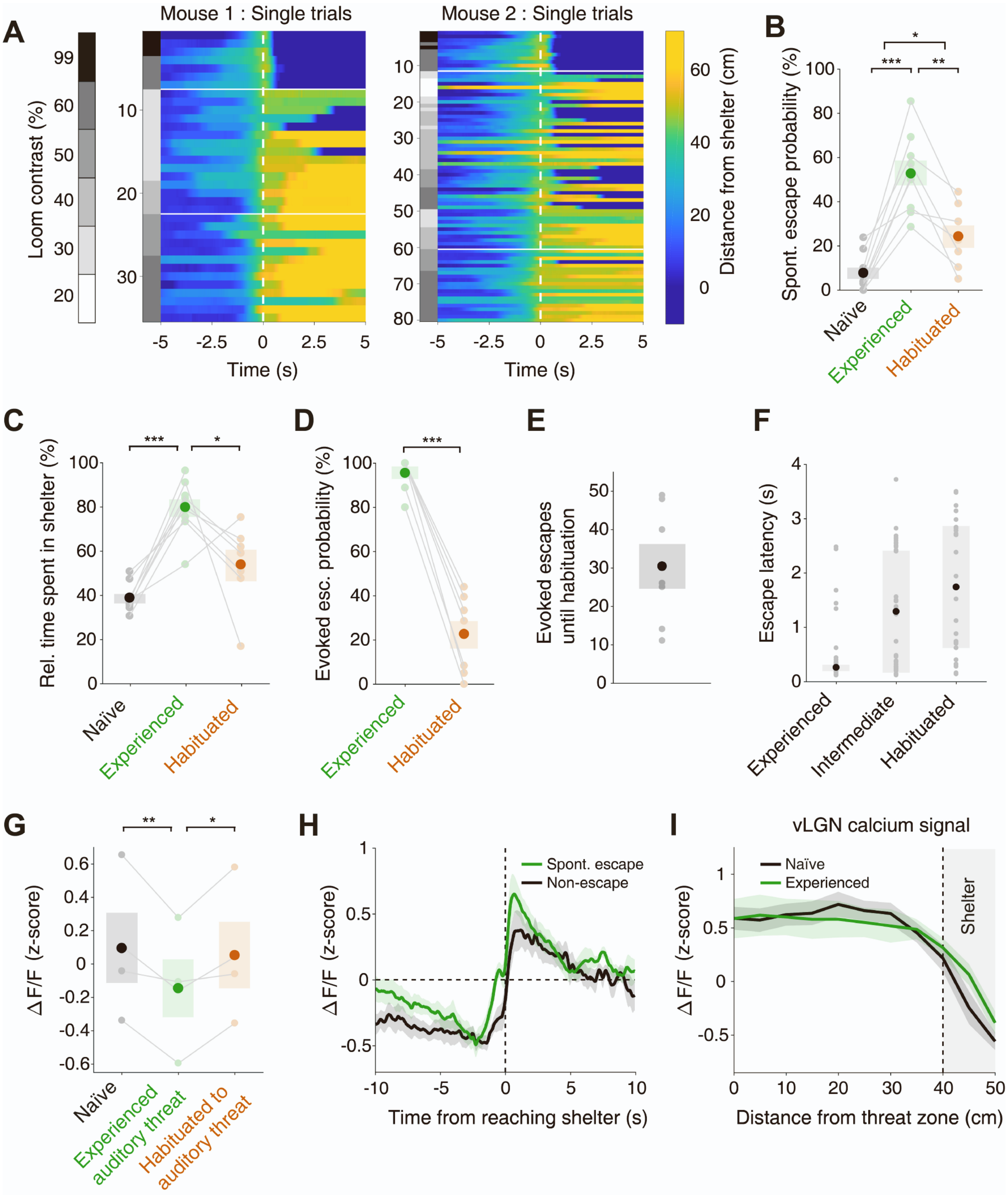

Supplemental Figure S4. Behavior and calcium signals during repeated exposure to threat stimuli. Related to Figure 1.

(A) Consecutive single trials of looming stimulus presentations of two example animals during photometry experiments, showing the mice's distance from the shelter (shelter position -10 to 0 cm) over time, aligned to stimulus onset (white dashed vertical line). Trials are shown in rows in chronological order from top to bottom. Stimulus contrast is indicated on the left of each trial. Top horizontal white line indicates the start of the habituation protocol; bottom horizontal white line indicates the timepoint after which the animal was considered habituated (when three consecutive trials of 50 - 60% contrast looming stimuli did not trigger an escape). **(B)** Mean spontaneous escape probability out of all returns to the shelter not triggered by a looming stimulus, in the period before presentation of the first looming stimulus (naïve; black, n = 9 mice), after presentation of the first looming stimulus (experienced; green, n = 9 mice), and after habituation (orange, n = 7 mice). Pale dots represent data from single animals. Error bars represent SEM across mice. Naïve - experienced: $p = 2.47\times10^{-6}$, experienced - habituated: $p = 1.13\times10^{-3}$, naïve - habituated: $p = 0.0269$. (Tukey's honest significance test preceded by repeated measures one-way analysis of variance, $p = 3.58\times10^{-6}$). **(C)** Mean relative time spent in the shelter in naïve (black, n = 9 mice), experienced (green, n = 9 mice), and habituated mice (orange, n = 7 mice). Pale dots represent data from single animals. Error bars represent SEM across mice. Naïve - experienced: $p = 8.63\times10^{-5}$, experienced - habituated: $p = 0.0145$, naïve - habituated: $p = 0.106$. (Tukey's honest significance test preceded by repeated measures one-way analysis of variance, $p = 1.22\times10^{-4}$). **(D)** Mean escape probability in response to intermediate-contrast looming stimuli (50% - 60%) in experienced (green) and habituated (orange) mice; n = 7 mice. Pale dots represent data from single animals. Error bars represent SEM across mice. $p = 7.05\times10^{-6}$, dependent t-test for paired samples. **(E)** Mean number of looming-stimulus evoked escapes before animals were considered habituated; n = 7 mice. Pale dots represent data from single animals. Error bars represent SEM across mice. **(F)** Median escape latency to intermediate-contrast (50% - 60%) looming stimuli in experienced animals (after the first looming stimulus, before the start of the habituation protocol, n = 47 escapes), during the habituation protocol (intermediate, n = 40 escapes) and after habituation (habituated, n = 30 escapes). Pale dots represent data from single escapes. Error bars represent IQR across escapes. **(G)** Calcium signals of vLGN axons in mSC in mice presented with auditory threat stimuli (loud 15 kHz tones, see Methods). Mean calcium activity in mice approaching the threat-zone before presentation of the first auditory threat stimulus (black, naïve), after presentation of the first auditory threat stimulus (green, experienced) and after habituation (orange). Pale dots represent data from single animals; n = 4 mice. Error bars represent the SEM across mice. Naïve – experienced: $p = 6.76\times10^{-3}$, experienced – habituated: $p = 0.017$, naïve – habituated: $p = 0.683$. (Tukey's honest significance test preceded by repeated measures one-way analysis of variance, $p = 6.07\times10^{-3}$). **(H)** Mean calcium activity in vLGN axons aligned to the moment at which mice reach the shelter following a spontaneous escape (green; initiated with a turn towards the shelter and reaching the shelter within 2.5 s) and after a slow, non-escape return to the shelter (black, speed < 40 $cm\times s^{-1}$ in the 2 s before reaching shelter). **(I)** Mean calcium activity in vLGN (measured with a fiber over vLGN) binned by distance during threat zone approach in naïve mice (black, n = 7 mice) and after presentation of the first looming stimulus (green, experienced, n = 7 mice). Shading shows SEM across mice. Dashed line represents the shelter location.

Supplemental Figure S5

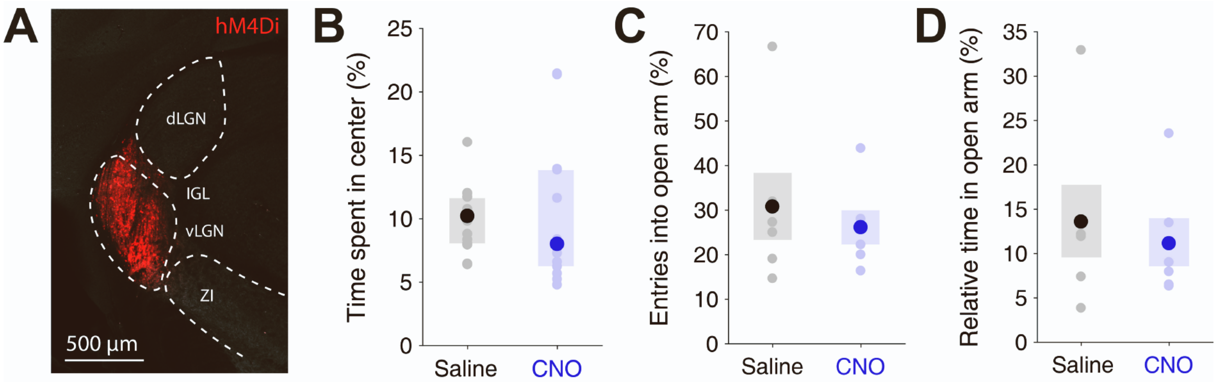

Supplemental Figure S5. hM4Di construct expression and control experiments testing effects of CNO. Related to Figure 2.

(A) Expression of hM4Di in vLGN GABAergic neurons in VGAT-Cre mice. **(B)** Median relative time spent in the center during 5 minutes in the open field test in systemic CNO- (blue, n = 14 mice) and saline-injected (black, n = 14 mice) wild-type control animals. Pale dots represent data from single animals. Error bars represent IQR across mice. p = 0.370, Wilcoxon rank-sum test. **(C)** Mean entries into open arms as a percentage of total arm entries during 15 minutes on the elevated plus maze in systemic CNO- (blue, n = 6 mice) and saline-injected (black, n = 6 mice) wild-type control animals. Pale dots represent data from single animals. Error bars represent SEM across mice. p = 0.599, independent two-sample t-test. **(D)** Mean relative time spent in open arms during 15 minutes on the elevated plus maze in CNO- (blue, n = 6 mice) and saline-injected (black, n = 6 mice) wild-type control animals. Pale dots represent data from single animals. Error bars represent SEM across mice. p = 0.632, independent two-sample t-test.

Supplemental Figure S6

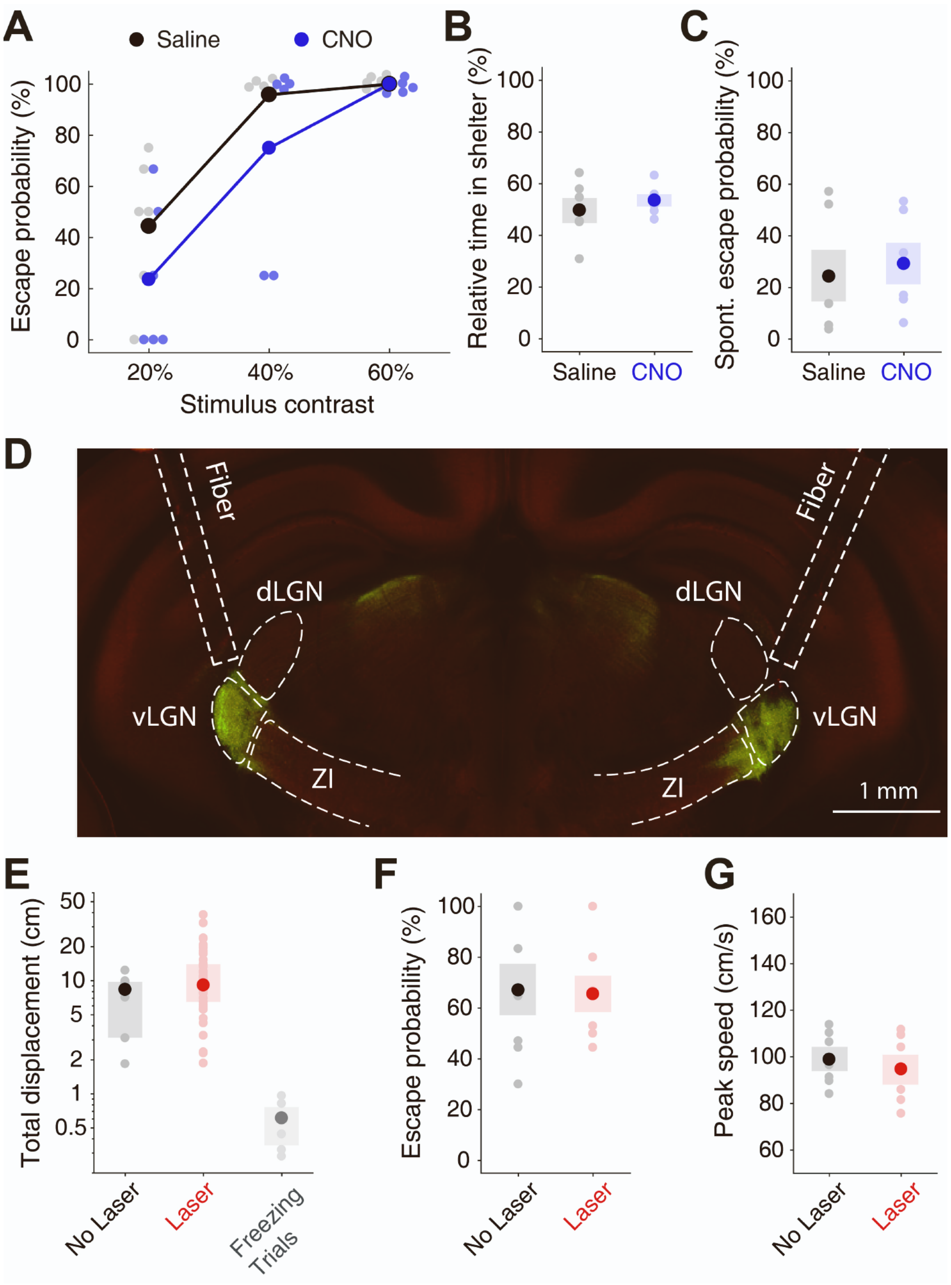

Supplemental Figure S6. Control experiments testing effects of CNO and laser. Related to Figure 3.

(A) Mean escape probability in response to looming stimuli of different contrasts for CNO- (blue, n = 6 mice) or saline-injected (black, n = 6 mice) wild-type control animals. Pale dots represent data from single animals. **(B)** Mean relative time spent in the shelter after exposure to the first looming stimulus for CNO- (blue, n = 6 mice) or saline-injected (black, n = 6 mice) wild-type control animals. Pale dots represent data from single animals. Error bars represent SEM across mice. p = 0.345, independent two-sample t-test. **(C)** Mean spontaneous escape probability after exposure to the first looming stimulus for CNO- (blue, n = 6 mice) or saline-injected (black, n = 6 mice) wild-type control animals.

Pale dots represent data from single animals. Error bars represent SEM across mice. p = 0.639, independent two-sample t-test. **(D)** Example image of bilateral ChR2 expression in vLGN GABAergic neurons after injection of AAV-flex-ChR2 in vGAT-Cre mice with indicated position of optic fibers. The fiber track on the left hemisphere is more evident in a more anterior slice. **(E)** Median change in the mouse's position during presentation of high-contrast looming stimuli in non-escape trials without optogenetic stimulation (black, n = 6 out of 47 trials), in non-escape trials with laser stimulation of ChR2-expressing vLGN neurons (red, n = 47 out of 47 trials), and in the few trials out of all optogenetic experiments in which freezing was observed in response to low- and intermediate-contrast looming stimuli (grey, n = 7 out of 266 trials). Pale dots represent data from single trials. Error bars represent IQR across trials. **(F)** Mean escape probability in response to looming stimuli without (black) and with laser stimulation (red) in all control mice with either expression of GFP in vLGN GABAergic neurons (n = 3 mice) or without fluorescent construct expression in vLGN (n = 4 mice). Pale dots represent data from single animals. Error bars represent SEM across mice. p = 0.806, dependent t-test for paired samples. **(G)** Mean peak running speed during escapes to looming stimuli without (black) and with laser stimulation (red) in control mice without expression of ChR2 in vLGN neurons; n = 7 mice. Pale dots represent data from single animals. Error bars represent SEM across mice. p = 0.430, dependent t-test for paired samples.

Supplemental Figure S7

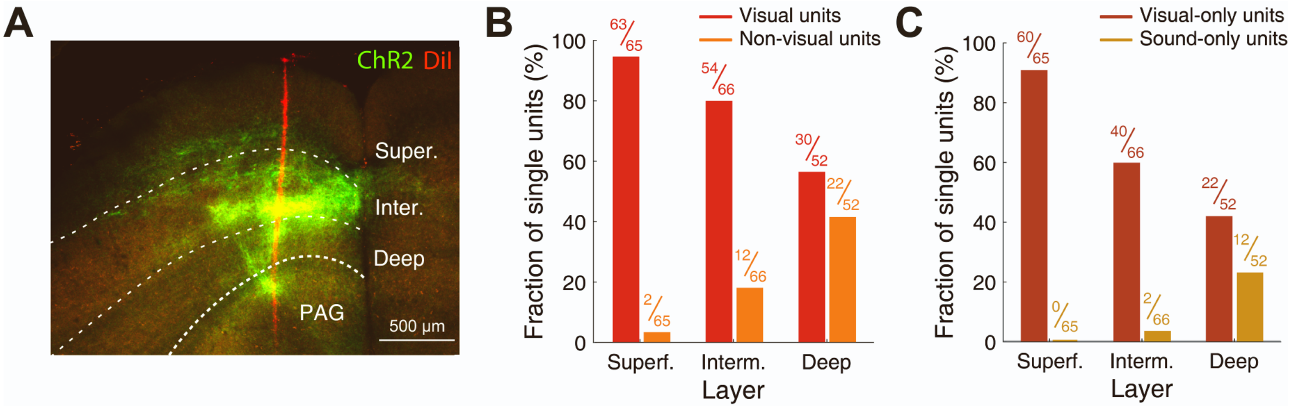

Supplemental Figure S7. In vivo response properties of mSC neurons in different layers. Related to Figure 4.

(A) Example image showing ChR2-labelled vLGN axons (green) in mSC and the placement of the Neuropixels probe (red DiI track) for extracellular electrophysiological recordings. mSC was divided into anatomical layers for analysis as indicated by dashed white lines; Super.: superficial layers (zonal, superficial grey and optic layers), Inter.: intermediate layers (intermediated grey and intermediate white layers); Deep: deep layers (deep grey and deep white layers). **(B)** Layer-wise distribution of single units responding to either looming or grating stimuli (visual, red), and of single units not responding to any visual stimulus (non-visual, orange). Single units from 11 recordings in 6 animals. **(C)** Layer-wise distribution of single units responding to either looming or grating stimuli, but not to sounds (visual-only, dark red), and of single units not responding to any visual stimulus but showing a significant response to sounds (sound-only, dark orange). Single units from 11 recordings in 6 animals.

Supplemental Figure S8

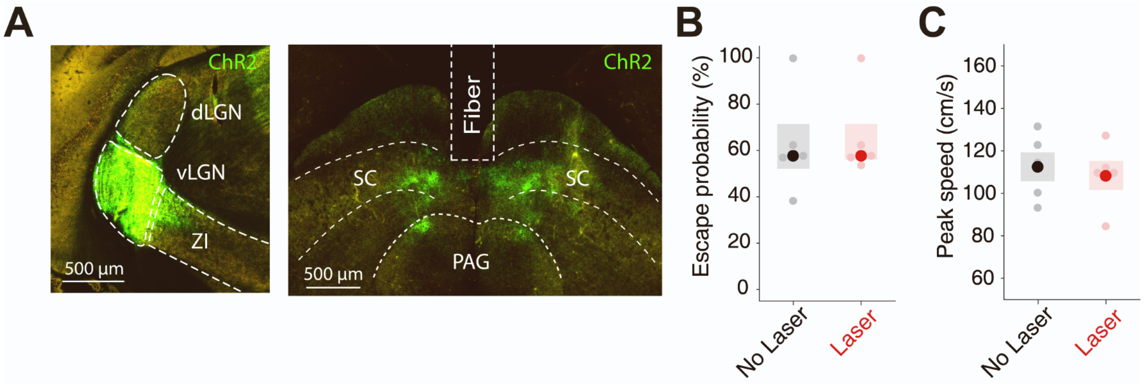

Supplemental Figure S8. ChR2 expression and control experiments to test for laser effect. Related to Figure 5.

(A) Example images of ChR2 expression in vLGN GABAergic neurons (left) and ChR2-labelled vLGN axons in SC with indicated optic fiber position (right). ChR2 was expressed bilaterally, one fiber was placed over SC at the midline. **(B)** Median escape probability in response to high-contrast looming stimuli without (black) and with laser stimulation in mSC (red) in control mice with either expression of GFP in vLGN neurons or without fluorescent construct expression in vLGN; n = 5 mice. Pale dots represent data from single animals. Error bars represent IQR across mice. p = 1.00, Wilcoxon signed-rank test. **(C)** Mean peak running speed during escapes to high-contrast looming stimuli with (black) and without laser stimulation in mSC (red) in control mice without expression of ChR2 in vLGN neurons, similar to B; n = 5 mice. Pale dots represent data from single animals. Error bars represent SEM across mice. p = 0.353, dependent t-test for paired samples.

Supplemental Figure S9

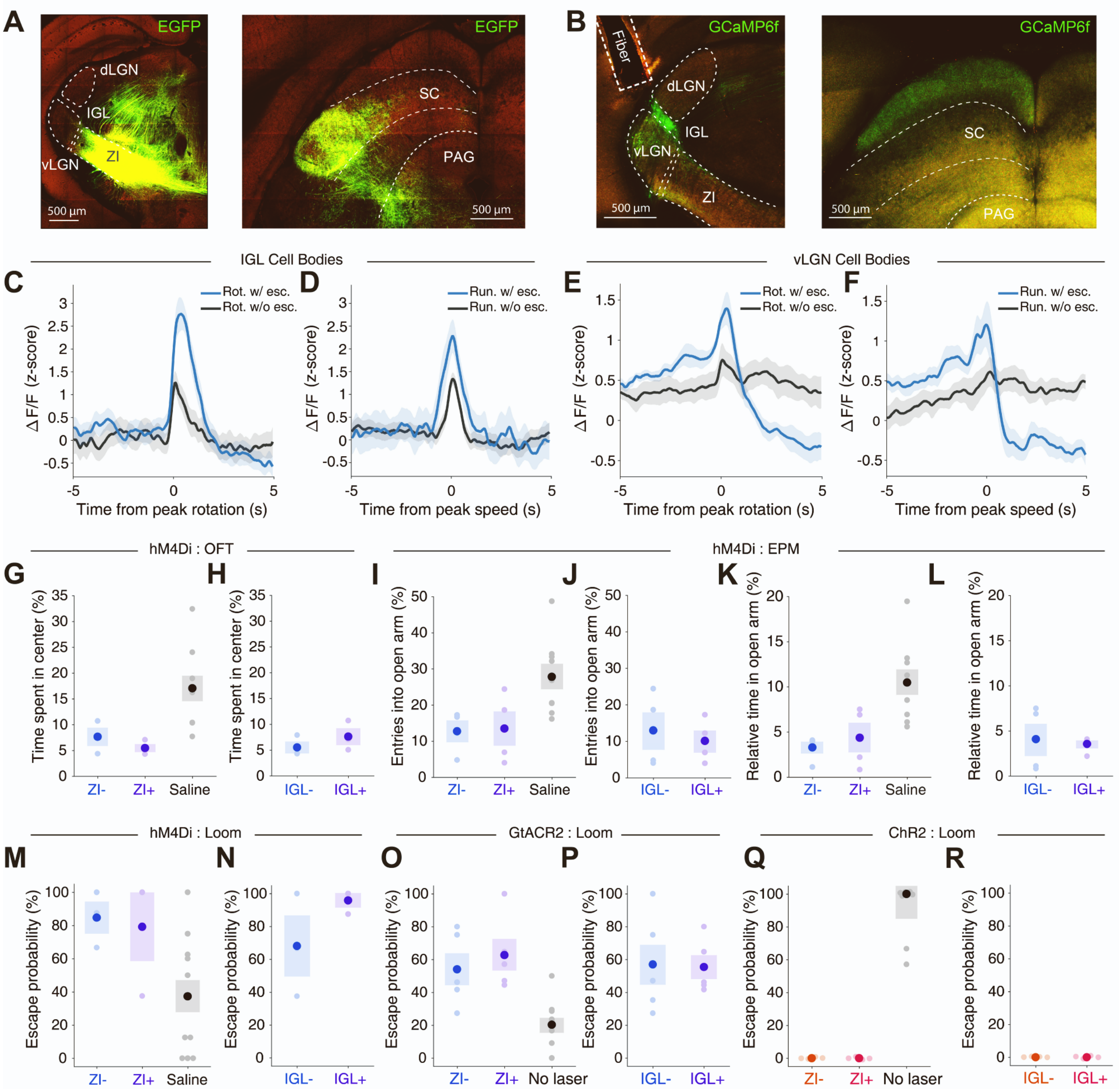

Supplemental Figure S9. Control analyses testing the involvement of IGL and ZI. Related to STAR Methods.

(A) Example images of EGFP expression in zona incerta (ZI) neurons in a WT animal from the Allen Mouse Brain Connectivity Atlas (left, Experiment 113095845) and corresponding ZI axons in lateral SC (right). **(B)** Example images of GCaMP6f expression in GABAergic intergeniculate leaflet (IGL) neurons with track indicating fiber position for photometry recordings (left) and corresponding GABAergic IGL axons in superficial layers in SC (right). **(C,D)** Mean calcium activity in IGL GABAergic neurons (GCaMP expression restricted to IGL, as depicted in **B**; fiber over IGL) during fast body rotations, aligned to peak angular velocity **(C)**, and during bouts of fast running speeds,

aligned to peak running speed **(D)**, in escape (blue) and non-escape (black) trials. Shading shows 95% confidence interval of the mean across trials; n = 1 mouse. **(E,F)** Mean calcium activity in vLGN GABAergic neurons (fiber over vLGN) during fast body rotations, aligned to peak angular velocity **(E)**, and bouts of fast running speeds, aligned to peak running speed **(F)**, in escape (blue) and non-escape (black) trials. Shading shows SEM across mice; n = 7 mice. **(G)** Mean relative time spent in the center during 5 minutes in the open field test of animals injected with CNO with the least hM4Di expression in Zona Incerta (ZI-, light blue, n = 3 mice), in animals injected with CNO with the most hM4Di expression in ZI (ZI+, purple, n = 3 mice, data divided in two halves here and in the following figure panels), and in saline-injected animals (black, n = 9 mice). Pale dots represent data from single animals. Error bars represent SEM across mice. ZI- vs ZI+ : p = 0.338, independent two-sample t-test. **(H)** Same as **G** but in animals injected with CNO with the least hM4Di expression in the intergeniculate leaflet (IGL-, light blue, n = 3 mice), and in animals injected with CNO with the most hM4Di expression in IGL (IGL+, purple, n = 3 mice). p = 0.363, independent two-sample t-test. **(I)** Mean entries into open arms as a percentage of total arm entries during 15 minutes on the elevated plus maze in animals injected with CNO with the least hM4Di expression in ZI (ZI-, light blue, n = 4 mice), in animals injected with CNO with the most hM4Di expression in ZI (ZI+, purple, n = 4 mice), and in saline-injected animals (black, n = 9 mice). Pale dots represent data from single animals. Error bars represent SEM across mice. ZI- - ZI+ : p = 0.898, independent two-sample t-test. **(J)** Same as **I** but in animals injected with CNO with the least hM4Di expression in IGL (IGL-, light blue, n = 4 mice), and in animals injected with CNO with the most hM4Di expression in IGL (IGL+, purple, n = 4 mice). p = 0.643, independent two-sample t-test. **(K)** Mean relative time spent in open arms during 15 minutes on the elevated plus maze in animals injected with CNO with the least hM4Di expression in ZI (ZI-, light blue, n = 4 mice), in animals injected with CNO with the most hM4Di expression in ZI (ZI+, purple, n = 4 mice), and in saline-injected animals (black, n = 9 mice). Pale dots represent data from single animals. Error bars represent SEM across mice. ZI- - ZI+ : p = 0.578, independent two-sample t-test. **(L)** Same as **K** but in animals injected with CNO with the least hM4Di expression in IGL (IGL-, light blue, n = 4 mice), and in animals injected with CNO with the most hM4Di expression in IGL (IGL+, purple, n = 4 mice). p = 0.786, independent two-sample t-test. **(M)** Mean escape probability in response to low contrast stimuli (20% and 40%) in animals injected with CNO with the least hM4Di expression in ZI (ZI-, light blue, n = 3 mice), in animals injected with CNO with the most hM4Di expression in ZI (ZI+, purple, n = 3 mice), and in saline-injected animals (black, n = 12 mice). Pale dots represent data from single animals. Error bars represent SEM across mice. ZI- - ZI+ : p = 0.866, independent two-sample t-test. **(N)** Same as **M,** but in animals injected with CNO with the least hM4Di expression in IGL (IGL-, light blue, n = 3 mice), and in animals injected with CNO with the most hM4Di expression in IGL (IGL+, purple, n = 3 mice). p = 0.331, independent two-sample t-test. **(O)** Mean escape probability in response to low-contrast looming stimuli (30% - 40%) in bilateral vLGN inhibition trials in animals with the least GtACR2 expression in ZI (ZI-, light blue, n = 5 mice), in animals with the most GtACR2 expression in ZI (ZI+, purple, n = 5 mice), and in control trials (black, n = 10 mice). Pale dots represent data from single animals. Error bars represent SEM across mice. ZI- - ZI+ : p = 0.56, independent two-sample t-test. **(P)** Same as **O**, but in animals with the least GtACR2 expression in IGL (IGL-, light blue, n = 5 mice) and in animals with the most GtACR2 expression in IGL (IGL+, purple, n = 5 mice). p = 0.912, independent two-sample t-test. **(Q)** Median escape probability in response to high-contrast looming stimuli (99%) in bilateral

vLGN stimulation trials in animals with the least ChR2 expression in ZI (ZI-, orange, n = 4 mice), in animals with the most ChR2 expression in ZI (ZI+, pink, n = 4 mice) and in control trials (black, n = 8 mice). Pale dots represent data from single animals. Error bars represent IQR across mice. ZI- - ZI+ : p = 1.00, Wilcoxon rank-sum test. **(R)** Same as **Q**, but in animals with the least ChR2 expression in IGL (IGL-, orange, n = 4 mice) and in animals with the most ChR2 expression in IGL (IGL+, pink, n = 4 mice). p = 1.00, Wilcoxon rank-sum test.

Supplemental Figure S10

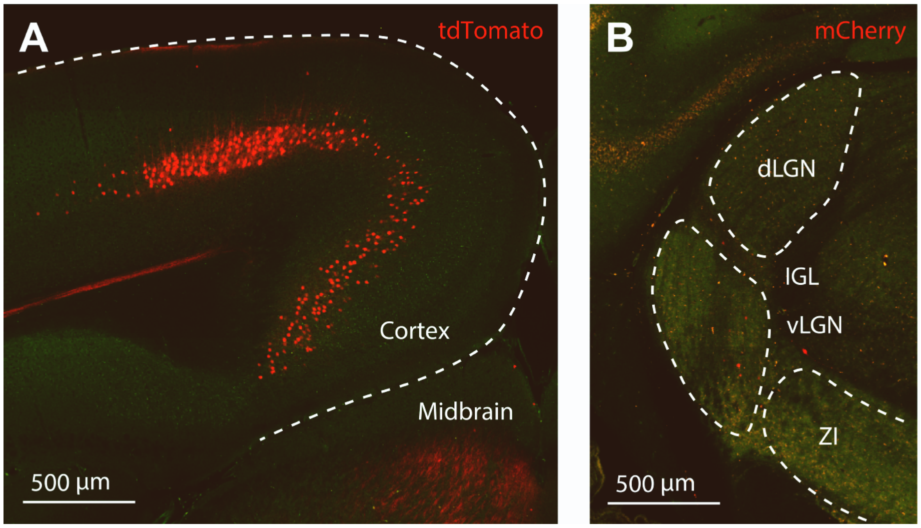

Supplemental Figure S10. Non-efficient retrograde transport of retroAAV in vLGN projections to mSC. Related to STAR Methods.

(A) Cre-dependent tdTomato expression in cortical cells after the injection of AAVretro-hSyn-Cre in the medial superior colliculus in a tdTomato-reporter mouse (Ai14D). **(B)** Very sparse Cre-dependent expression of mCherry in vLGN after the injection of AAVretro-hSyn-Cre in the medial superior colliculus and a large injection of AAV1-EF1a-FLEX-mCherry in the vLGN of a WT mouse (C57BL/6J).
